# Biomolecular engineering for nanobio/bionanotechnology

**DOI:** 10.1186/s40580-017-0103-4

**Published:** 2017-04-24

**Authors:** Teruyuki Nagamune

**Affiliations:** Department of Chemistry and Biotechnology, Graduate School of Engineering, The University of Tokyo, Tokyo, Japan

**Keywords:** Engineered biological molecules, Therapy, Diagnosis, Biosensing, Bioanalysis, Biocatalyst, Nucleic acid engineering, Gene engineering, Protein engineering, Conjugation technologies

## Abstract

Biomolecular engineering can be used to purposefully manipulate biomolecules, such as peptides, proteins, nucleic acids and lipids, within the framework of the relations among their structures, functions and properties, as well as their applicability to such areas as developing novel biomaterials, biosensing, bioimaging, and clinical diagnostics and therapeutics. Nanotechnology can also be used to design and tune the sizes, shapes, properties and functionality of nanomaterials. As such, there are considerable overlaps between nanotechnology and biomolecular engineering, in that both are concerned with the structure and behavior of materials on the nanometer scale or smaller. Therefore, in combination with nanotechnology, biomolecular engineering is expected to open up new fields of nanobio/bionanotechnology and to contribute to the development of novel nanobiomaterials, nanobiodevices and nanobiosystems. This review highlights recent studies using engineered biological molecules (e.g., oligonucleotides, peptides, proteins, enzymes, polysaccharides, lipids, biological cofactors and ligands) combined with functional nanomaterials in nanobio/bionanotechnology applications, including therapeutics, diagnostics, biosensing, bioanalysis and biocatalysts. Furthermore, this review focuses on five areas of recent advances in biomolecular engineering: (a) nucleic acid engineering, (b) gene engineering, (c) protein engineering, (d) chemical and enzymatic conjugation technologies, and (e) linker engineering. Precisely engineered nanobiomaterials, nanobiodevices and nanobiosystems are anticipated to emerge as next-generation platforms for bioelectronics, biosensors, biocatalysts, molecular imaging modalities, biological actuators, and biomedical applications.

## Introduction

Nanotechnology is the creation and utilization of materials, devices, and systems through controlling matter on the nanometer scale, and it is the key technology of the twenty-first century. The ability to exploit the structures, functions and processes of biological molecules, complexes and nanosystems to produce novel functional nanostructured biological materials has created the rapidly growing fields of nanobiotechnology and bionanotechnology, which are fusion research fields of nanotechnology and biotechnology [1]. Although these words are often used interchangeably, in this review, they are utilized in terminologically different ways, as follows.

Nanobiotechnology is used in relation to the ways in which nanotechnology is used to create materials, devices and systems for studying biological systems and developing new biological assay, diagnostic, therapeutic, information storage and computing systems, among others. These systems use nanotechnology to advance the goals of biological fields. Some nanobiotechnologies scale from the top down, such as from microfluidics to nanofluidic biochips (e.g., lab-on-a-chip for continuous-flow separation and the detection of such macromolecules as DNA and proteins [2], point-of-care biosensors for detecting biomarkers and clinical diagnosis [3–7], and solid-state nanopore sensors for DNA sequencing [8]). Other nanobiotechnologies scale from the bottom up for the fabrication of nanoscale hybrid materials, such as complexes consisting of nanoparticles (NPs) (e.g., magnetic NPs, AuNPs and AgNPs, silica NPs, quantum dots (QDs), polymeric micelles, liposomes, dendrimers, and fullerenes) and biological molecules, which are highly useful for biosensing, bioimaging, diagnostic and therapeutic applications in healthcare [9–15].

On the other hand, bionanotechnology refers to the ways in which biotechnology is used to improve existing or create new nanotechnologies through the study of how biological systems work and the applications of biological molecules and systems to nanotechnology. DNA and RNA nanotechnologies, the utilization of the base-pairing and molecular self-assembly properties of nucleic acids to create useful materials, such as DNA origami, DNA nanomachines, DNA scaffolds for electronics, photonics and protein arrays, and DNA and RNA aptamers, ribozymes and riboswitches, are important examples of bionanotechnology [16, 17]. Another important area of research involves taking advantage of the self-assembly properties of peptides, proteins and lipids to generate well-defined 3D structures, functional protein complexes, nanofilms and other nanostructures, such as micelles, reverse micelles and liposomes, which could be used as novel approaches for the large-scale production of programmable nanomaterials [18–20]. The application of carbohydrate polymers combined with nanotechnology in tissue engineering and medicine are also potential research fields for the development of novel biomaterials for biosensing, bioimaging, diagnostic and drug-delivery systems [21].

With either nanobiotechnology or bionanotechnology, biological molecules are indispensable building blocks for fabricating functional nanomaterials, nanodevices and nanosystems. However, from the viewpoint of applying biological materials to nanotechnology, biological materials found in nature always have sufficient functions and properties. Recent advances in biomolecular engineering, such as genetic engineering, DNA and RNA engineering, protein engineering, site-specific chemical and enzymatic conjugation technologies, self-assembly technology and massive high-throughput screening (HTS) methods, have enabled us to improve, stabilize, integrate and alter the functions and properties of biological materials. Thus, it is possible to create engineered biological materials with functions and properties that are optimized for various uses in the fields of bioelectronics, biosensors, biocatalysis, molecular imaging, biological actuators, drug delivery systems, biomaterials for tissue engineering and regenerative medicine.

In this review, recent studies applying engineered biological materials to nanobio/bionanotechnology are discussed, and various biomolecular engineering technologies are highlighted.

## Application of engineered biological molecules to nanobio/bionanotechnology

**Fig. 1:**
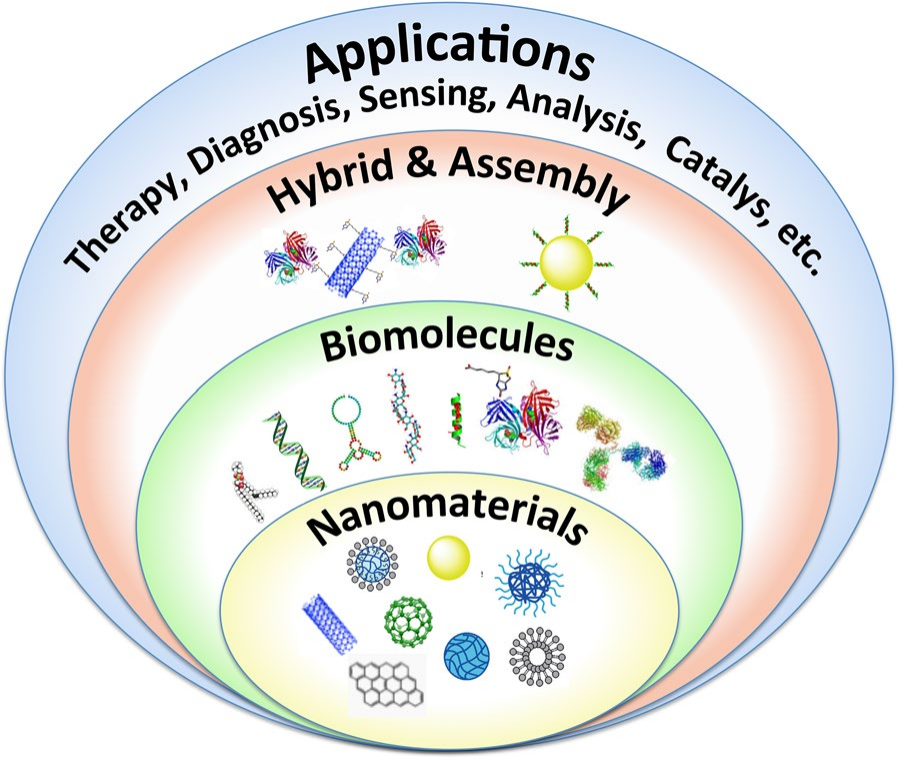
A summary of nanobiomaterials and their applications

Nanobio/bionanotechnology has created new opportunities for advances in diverse fields, including life science, medicine, electronics, engineering, and biotechnology. Nanoscale materials [e.g., NPs, nanowires, nanofibers, and nanotubes (NTs)] combined with various engineered biological molecules (e.g., proteins, enzymes, oligonucleotides, polysaccharides, lipids, biological cofactors and ligands) have been explored in many biological applications (e.g., therapy, diagnosis, bioimaging, biosensing, bioanalysis, biocatalysis, cell and organ chips, bioelectronic devices, and biological separation) (Fig. 1). Their novel and unique properties and functions, such as high volume-to-surface ratio, improved solubility, quantum size, macroscopic quantum tunnel and multifunctionality, result in nanobiomaterials that are drastically different from their corresponding bulk materials.

The current review is focused on advances in the development of nanobiomaterials for applications in therapy, diagnosis, biosensing, bioanalysis and biocatalysis because nanobiomaterials for cell and organ chips [22–25], bioelectronic devices [26, 27] and biological separation [28] have recently been reviewed in this journal.

### Nanobiomaterials for therapy and diagnosis

Smart therapeutic and diagnostic or bioimaging NPs carrying cargo materials, such as drugs, DNAs, RNAs, proteins, and imaging reagents, have been widely developed [11, 13, 29–33]. To achieve intracellular NP and drug delivery, many strategies for overcoming various biological barriers are needed, including the following: (i) preventing removal from the circulation by cells of the reticuloendothelial system; (ii) targeting specific cells; (iii) internalization into cells; (iv) escaping from endosomes; (v) trafficking to specific organelles; and (vi) controlling the release of payloads (e.g., drugs, DNAs or RNAs).

#### Preventing removal from the circulation

NPs made of hydrophobic synthetic polymers, metals or inorganic materials are usually not blood compatible. Their injection into the body can provoke a coagulation response and activate the complement cascade; subsequently, they can be recognized by phagocytes and macrophages, rendering them useless or harmful. The surface modification of NPs with hydrophilic synthetic or biological polymers, such as polyethylene glycol (PEG) [34], heparin [35] or dextran [36], forms a steric brush that imparts resistance to protein adsorption. This type of surface modification shows increased intrinsic anticoagulant and anti-complement properties, as well as other biological activities; in addition, it extends the circulation half-life and reduces the immunogenicity of NPs in the human body. The conformation of polymer chains on the surface also influences the pharmacokinetics and biodistribution of NPs.

#### Targeting specific cells

The surface modification of NPs with biological ligands, such as folate, arginine-glycine-aspartate (RGD) peptides, aptamers, transferrin, antibodies or small antibody fragments, facilitates NP targeting, imaging and internalization into specific cells, e.g., cancer cells, and tumor tissues.

Folate is a well-known small molecule frequently used as a cancer cell-targeting ligand that binds to folate receptors with high affinity. The chemical conjugation of folate onto the surface of NPs can significantly promote their targeted delivery into cancer cells that overexpress folate receptors [37].

Proliferating tumors are known to generate new blood vessels. This process is an important feature of tumor development characterized by the unique overexpression of the integrins α_ν_β_3_ and α_ν_β_5_ by nascent endothelial cells during angiogenesis in various tumors, but not by ordinary endothelial cells. Peptides possessing the RGD sequence bind the integrins α_ν_β_3_ and α_ν_β_5_ with high affinity. Cyclic RGD peptides show higher affinity and stability than do linear RGD peptides, which allows their use for developing integrin-selective, targeting NPs [38].

Aptamers are short, single-stranded RNA or DNA oligonucleotides (15–40 bases) that can bind to target molecules with high affinity and specificity due to the ability of the molecules to fold into unique conformations with three-dimensional (3D) structures. A large number of aptamers have been screened against aberrantly activated proteins in cancer cells, such as vascular endothelial growth factor, platelet-derived growth factor, and nuclear factor kappa-light-chain-enhancer of activated B cells. Specific aptamers for targets can be selected from a large number of random sequences (libraries of 10^15^ random oligonucleotides) via the systematic evolution of ligands by exponential enrichment (SELEX) [39]. Aptamers generally have less immunogenicity, which can lead to improved biodistribution in the human body. NP surfaces can easily be conjugated with aptamers, and the conjugates show efficient cancer cell targeting and internalization [40]. Small molecules, peptides and aptamers are preferred for targeting and imaging ligands because they can be simply conjugated to NPs via facile chemical conjugation methods.

Transferrin (Tf) is a monomeric glycoprotein that can transport iron atoms into cells. Upon the binding of Tf to the Tf receptor (TfR), the Tf/TfR complex is internalized by cells through receptor-mediated endocytosis. TfR has been explored as a target for delivering anti-cancer drugs into cancer cells due to its overexpression by malignant tumor cells. TfR can be targeted by direct interaction with Tf displayed on the surface of NPs [41].

**Fig. 2:**
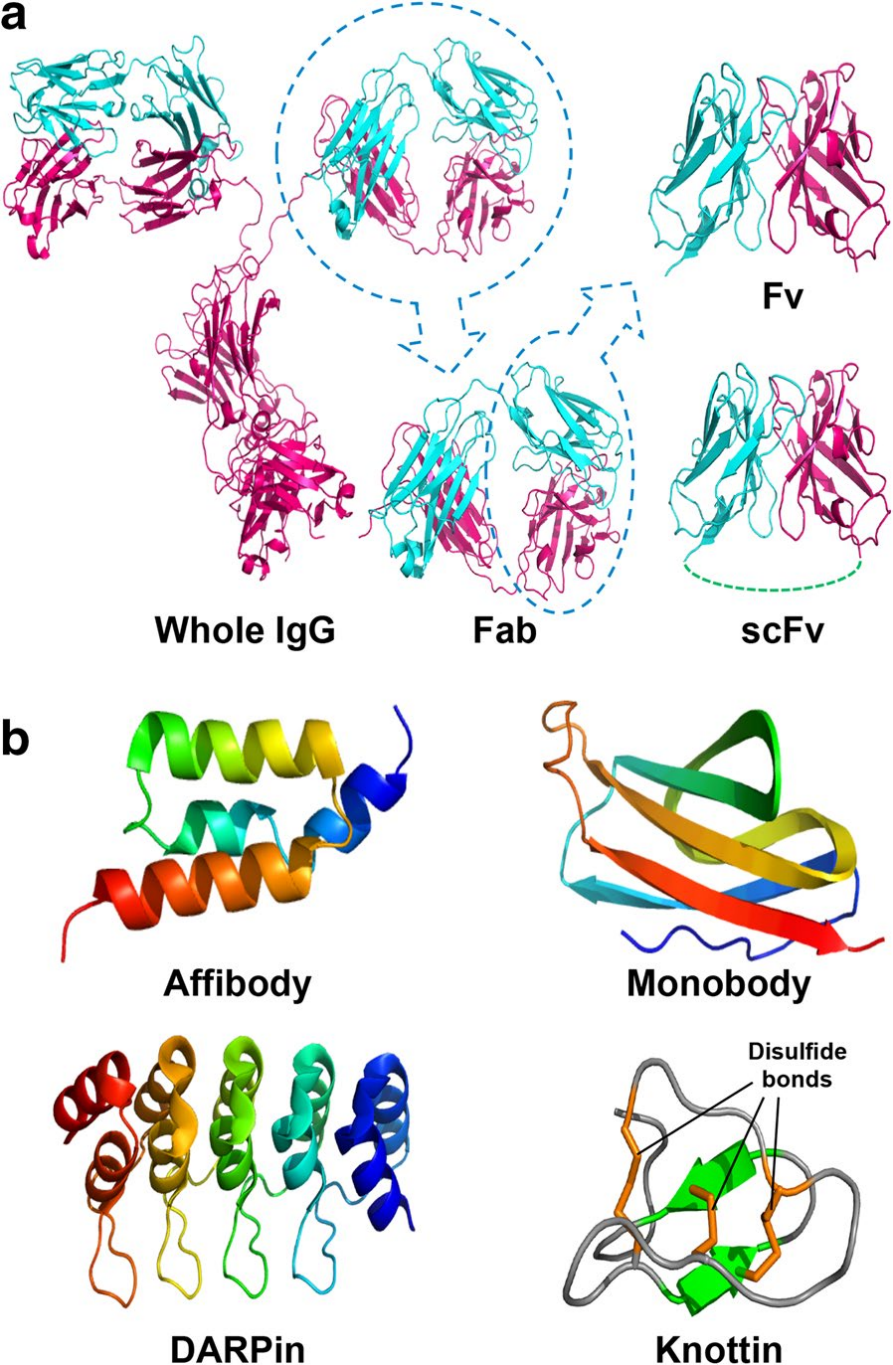
Targeting molecules. **a** IgG and its small fragments, **b** small molecular-binding scaffolds

Monoclonal IgG antibodies (mAbs) have been the preferred targeting molecules for receptors, membrane proteins and glyco-antigens on the surface of cancer cells. Because many breast cancer cells overexpress human epidermal growth factor receptor-2 (HER-2), NPs coated with anti-HER-2 antibodies can target breast cancer cells with high specificity. Similarly, epidermal growth factor receptor (EGFR) can be targeted by anti-EGFR antibodies. Despite the immense efforts directed toward their development, mAb-conjugated NPs still encounter many challenges and limitations, such as the difficulty or cost of manufacturing, immunogenicity, and penetration into tumor tissues, as mAbs are very large (150–170 kDa, 15–20 nm in diameter) and complex molecules. Alternatively, after proper engineering, small antibody fragments [e.g., antigen-binding fragment (Fab: ∼55 kDa) and variable fragment (Fv: ∼27 kDa)] can be used as they can retain the targeting affinity and specificity of the original whole antibody (Fig. 2a). For example, the single-chain variable fragment (scFv: ∼28 kDa) that consists of variable heavy- and light-chain domains connected with a flexible peptide linker can be used to target cells with high binding affinity and specificity.

Additionally, many alternative molecular scaffolds to mAbs have been investigated and developed in recent years, largely by the pursuit of much smaller (<20 kDa) targeting molecules with their putatively superior transport properties (Fig. 2b) [42]. These scaffolds include affibodies (∼8 kDa) with three-helix bundles structure derived from the Z domain of protein A, DARPin with three or more repeated small domains (∼6 kDa) consisting of two α-helices separated by a β-turn derived from ankyrin repeat proteins, and monobody with seven β-sheets forming a β-sandwich and three exposed loops from the 10th human fibronectin extracellular type III domain (∼10 kDa). These scaffolds are lacking disulfide bonds that make it possible to produce functional scaffolds regardless of the redox potential of the cellular environment, including the reducing environment of the cytoplasm and nucleus. Another scaffold is knottins (∼3.5 kDa) comprising a family of exceptionally small and highly stable proteins found in many species with structural homology involving a triple-disulfide stabilized knot motif. The randomization of loops or surfaces in conjunction with phage, ribosome or cell surface display technologies is used to engineer these molecular scaffolds and select binders to target molecules from many random libraries.

#### Internalization into cells

The surface modification of NPs with cell-penetrating peptides (CPPs) [43], such as the Tat peptide, penetrain, pVEC, transportan, MPG, Pep-1 and polyarginines, could facilitate the internalization of NPs into cells through either direct entry into the cytosol or endosomal pathways. The Tat peptide, penetrain and pVEC are short peptides (∼20-mers) derived from the basic domain of the HIV-1 trans-activator of transcription (Tat) protein, the third helix of the Antennapedia homeodomain and cadherin, respectively. Transportan, MPG and Pep-1 are chimeric peptides (∼30-mers) that are formed by the fusion of two natural sequences derived from galanin/mastoparan, HIV-gp41/SV40 T-antigen and HIV-reverse transcriptase/SV40 T-antigen, respectively. These CPPs mostly bear a net positive charge and consist of amino acid (AA) sequences with repeated basic AA units and hydrophobic or aromatic AAs. The repeated basic AA units might contribute to not only the binding of CPPs to the negatively charged cell surface but also the endosomal escape of CPPs via conformational change under the acidic pH conditions of late endosomes.

#### Endosomal escape

The endosomal-escape ability of NPs is indispensable for the delivery of NPs into the cytosol and to organelles within the cell. Peptide-based endosomal-escape agents have been developed, and these are derived from the small-peptide domains of several viral, bacterial and human sources [44]. For example, the HA2 subunit of the *Haemophilus influenzae* hemagglutinin (HA) protein of the influenza virus with a short chain of an N-terminal anionic peptide has shown fusogenic activity. At a low pH, the protonation of the glutamate (Glu) and the aspartate (Asp) causes a conformational change of this peptide from a random coil into an amphiphilic α-helical structure. This change allows the amphiphilic α-helical peptide to bind to the endosomal membrane, causing membrane disruption. A pH-sensitive peptide GALA with repeating glutamate-alanine-leucine-alanine (Glu-Ala-Leu-Ala) units could disturb the lipid bilayer by the same mechanism and facilitate the endosomal escape of GALA-modified NPs at acidic pH values. Arginin (Arg)-rich peptides and cationic peptides, also derived from viral proteins, could mimic the endosomal-disruptive properties of viral particles [45]. Several chemical polymers, such as polyethylenimine- and imidazole-containing polymers, with endosomal-disruptive properties have been reported. These polymers have a buffering capacity ranging from pH 5.0–7.2 and can promote endosome osmotic swelling and disruption via the proton sponge effect [46]. Recently, a conformation-switchable synthetic lipid consisting of two alkyl chains on a di(methoxyphenyl)-pyridine (pH-switchable unit) and a polar head group at the para position to the pyridine N atom was reported; upon protonation, hydrogen bonding induced a relative orientation change of the two alkyl chains, which disturbed the lipid packing of the membranes and conferred endosomal-escape properties [47].

#### Trafficking to specific organelles

In eukaryotic cells, proteins are specifically sorted during or after translation and delivered from the cytosol to target organelles, such as the nucleus, endoplasmic reticulum, peroxisomes and mitochondria. These proteins contain organelle-targeting peptide signals often found at the N-terminal extension consisting of a short, positively charged stretch of basic AAs and a long α-helical stretch of hydrophobic AAs [48, 49], and a database of protein localization signals has been constructed based on experimental protein localization [50]. Gene delivery systems for the gene therapy of chromosomal and mitochondrial DNA have been developed by chemically conjugating nuclear and mitochondrial targeting signal peptides to NPs consisting of therapeutic DNAs [51].

#### Controlling payload release

In many cases, NPs in the endosomes or the cytoplasm must collapse to allow the release of their payloads. Several strategies using stimulus-responsive moieties built into NPs have been utilized to improve the efficiency of controlled release [31]. These include pH-sensitive and thermal-sensitive polymers, which control interactions between payloads and NPs [52], and external stimulus-sensitive crosslinkers, which conjugate payloads with NPs [53], such as pH-labile linkers, photosensitive- and enzyme-cleavable linkers, and disulfide crosslinkers that are sensitive to a reducing intracellular environment.

The difference in pH values existing between healthy tissues (pH 7.4) and the extracellular environment of solid tumors (pH 6.5–6.8), as well as between the cytosol (pH 7.4) and endosomes (pH 5–6), has been extensively utilized to trigger the release of drugs into a specific organ or intracellular compartment. Polymers with functional groups that can alter the structure and hydrophobicity of NPs as a result of protonation or deprotonation in response to pH variation can be utilized in pH-sensitive polymeric NPs. Notable examples of pH-sensitive polymers include poly(acryl amide) (PAAm), poly(acrylic acid) (PAA), poly(methacrylic acid) (PMAA), poly(methyl acrylate) (PMA), poly(diethylaminoethyl methacrylate) (PDEAEMA), poly(diallyl dimethylammonium chloride) (PDDA) and poly(dimethyl aminoethyl methacrylate) (PDMAEMA).

Temperature-sensitive polymers and hydrogels exhibit a volume phase transition at a certain temperature, which causes a dramatic change in the hydration state. This phase transition reflects competing hydrogen-bonding properties, where intra- and intermolecular hydrogen bonding of the polymer molecules are favorable compared to the solubilization of the polymers by water. Examples of thermo-sensitive polymers are poly(*N*-isopropyl acrylamide) (PNIPAAm), poly(*N*,*N*-diethyl acrylamide) (PDEAAm), poly(methyl vinylether) (PMVE), poly(*N*-vinyl caprolactam) (PVCL), and poly(ethylene oxide)-poly(propylene oxide)-poly(ethylene oxide) (PEO-PPO-PEO).

In the case of polymer–drug conjugates, pH-sensitive linkages, such as oxime (pH < 5), hydrazone (pH < 5), hydrazide (pH < 5) and acetal (pH < 4–5), have been used to directly attach drug molecules to polymers. The use of light as a stimulus to trigger drug release has been actively explored owing to its high spatiotemporal resolution. Photosensitivity is often introduced to NPs through functional groups that can change their conformations and structures (e.g., azobenzene, pyrene, nitrobenzene and spirobenzopyran groups) or break their chemical bonds (e.g., arylcarbonylmethyl, nitroaryl, arylmethyl and coumarin-4-ylmethyl groups) upon irradiation [54, 55].

Enzymes perform a vast array of important functions inside our body. For example, hydrolytic enzymes overexpressed in cancer cells and tumor tissue can break certain bonds (e.g., ester, amide, glucuronide and phosphodiester bonds) within biopolymers, causing polymer structure disassembly or destruction. Notable examples of these enzymes are esterase, matrix metalloproteinase, β-glucuronidase and alkaline phosphatase. These enzymatic reactions can be utilized to trigger drug release [56].

#### Recent advances in targeted drug delivery and bioimaging

A major challenge of targeted drug delivery and bioimaging in therapeutics and diagnostics is the fabrication of NPs modified with various functional biomolecules for overcoming the above-mentioned biological barriers with a triggered cargo release system. Pluronic polymer-based micelles, to which folic acid (FA), redox-sensitive thiol groups and the anti-cancer drug doxorubicin (DOX) are chemically conjugated with pH-sensitive linkers, could be successfully delivered into multidrug-resistant (MDR) tumors in mice and exerted high cytotoxicity in the DOX-resistant MDR tumors by bypassing MDR efflux [57]. The carboxylate graphene oxide (GO)-based nanocarrier was multifunctionalized by poly(ethylene glycol) (PEG) terminated with an amino group and an FA group (FA–PEG–NH_2_) via the amidation reaction. The GO-based nanocarrier could adsorb large amounts of DOX on the GO surface via π–π stacking interactions at a neutral pH but release it at an acidic pH. The DOX-loaded FA–PEG-modified GO-based nanocarrier not only showed stable dispersibility and targetability to cancer cells with high FA receptor expression levels but also exhibited the low pH-activated controlled release of DOX in the endosomes of cells [58].

Nanohydrogels composed of filamentous bacteriophages and AuNPs, which were self-assembled via electrostatic interactions between the phage-capsid proteins and imidazole-modified AuNPs, have been developed and utilized for noninvasive imaging and targeted drug delivery in preclinical mouse models of breast and prostate cancer. The phage-based nanohydrogels could be multifunctionalized by fusing peptides, e.g., tumor-targeting ligands and CPPs, to phage-capsid proteins and by incorporating temperature-sensitive liposomes or mesoporous silica NPs containing imaging reagents and drugs. Because AuNPs packed densely within the nanohydrogel, their surface plasmon resonance shifted to the near-infrared (NIR) range, thereby allowing the NIR laser-mediated spatiotemporal photothermal release of cargo from temperature-sensitive liposomes [59]. Multifunctionalized AuNPs are generally constructed by the covalent assembly of an Au core with thiolated ligands. Novel multifunctionalized AuNPs have been assembled in one step by the nucleic acid hybridization of thiolated oligodeoxynucleotide-modified AuNPs with a library of functional molecule-conjugated complementary peptide nucleic acids (PNAs). The PNAs were functionalized by conjugation with 1,4,7,10-tetraazacyclododecane-1,4,7,10-tetraacetic acid for chelating ^64^Cu for positron emission tomography imaging, PEG for conferring stealth properties, and Cy5 for fluorescent imaging. These NPs demonstrated good stability in vivo by showing biodistribution behavior in mice [60].

**Fig. 3:**
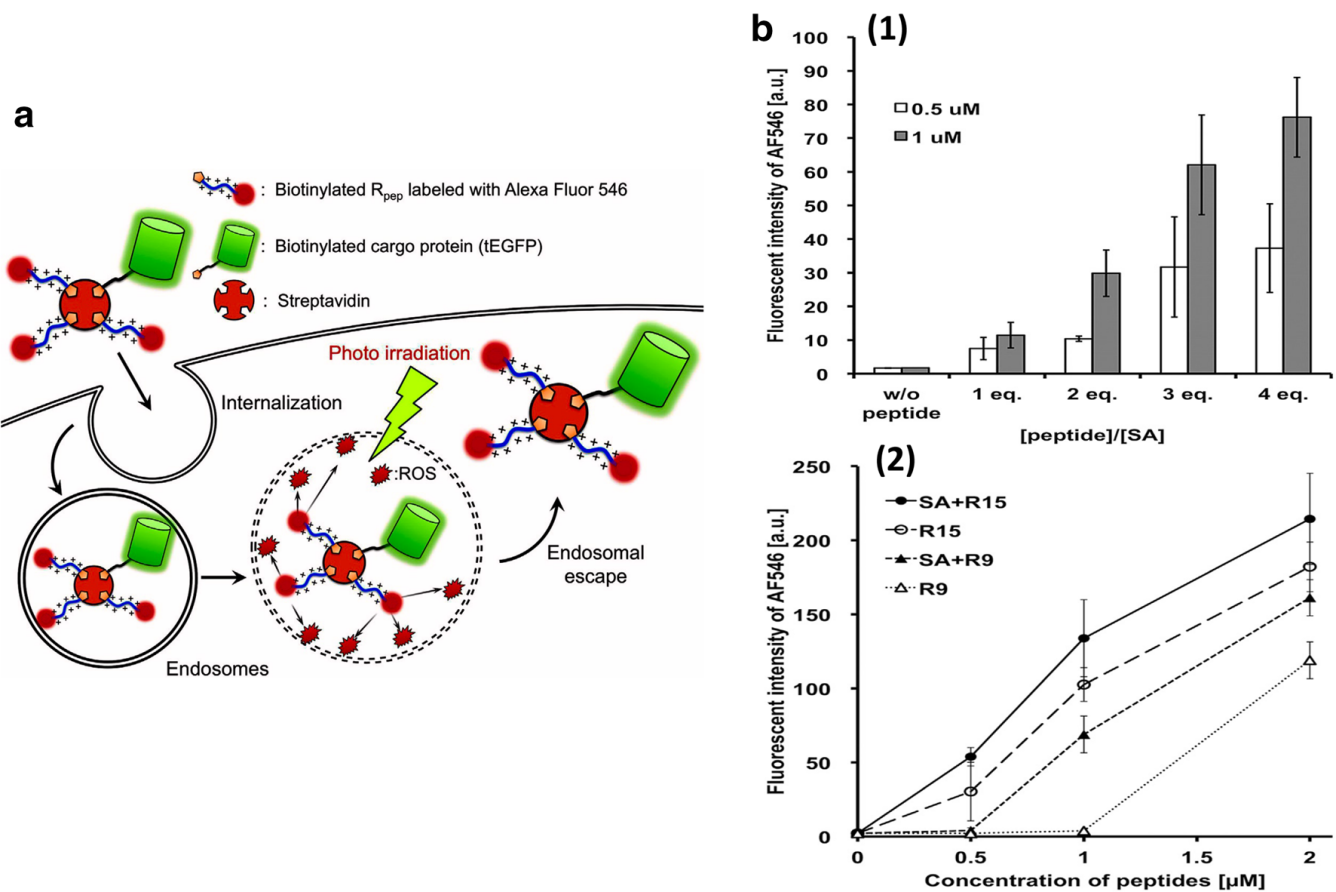
Protein transduction using the streptavidin based nano-carrier. **a** Schematic illustration of protein transduction using the streptavidin based nano-carrier. **b** (*1*) Effect of the conjugation ratio of R15 peptides to SA on the fluorescence intensity of HeLa cells after uptake of AF546-labeled SA–R15 complex. (*2*) Effects of the length of R_pep_ on the fluorescence intensity of HeLa cells after uptake of AF546-labeled R_pep_ itself ant SA–R_pep_ complex (Figure reproduced with permission from: Ref. [61]. Copyright (2015) with permission from Elsevier)

Recently, streptavidin (SA)-containing multifunctionalized NPs for carrying various biotinylated functional biomolecules have been reported. SA is a homo-tetramer protein, and each subunit can tightly bind to biotin molecule. We developed an SA-based cell-permeable nanocarrier equipped with photosensitizers as a versatile vehicle for spatiotemporally controlled cargo protein delivery into the cytosol (Fig. 3a) [61]. These nanocarriers can be prepared by attaching photosensitizer (Alexa Fluor 546: AF546)-modified biotinylated CPPs (oligo-arginine peptide R9 or R15) to a few biotin-binding sites of SA. Furthermore, a biotinylated target cargo protein is also loaded onto this carrier complex by using the remaining biotin-binding site of SA. Conjugation with more than three CPPs per SA significantly raised the cell-permeability of the SA–CPP complexes into HeLa cells (Fig. 3b). Under optimized conditions, the SA–CPP (R15) complex could be delivered into cells with both high efficiency and low cytotoxicity. Furthermore, the internalized AF546-modified SA complex could spatiotemporally escape from the endosome in a light-irradiated area.

**Fig. 4:**
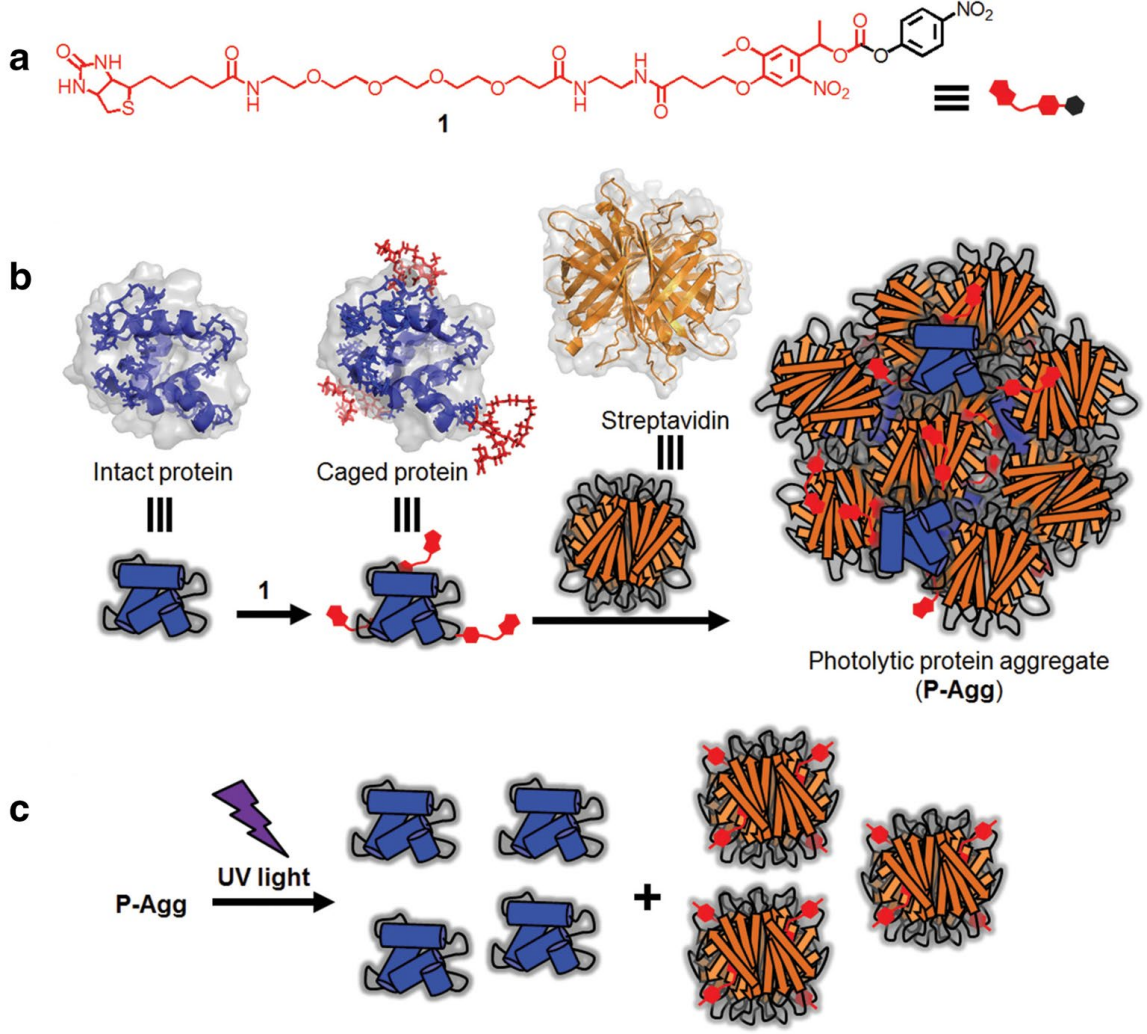
Schematic illustration of photolytic P-Aggs formation and light-induced release of active proteins. **a** The chemical structure of BCR 1 consisting of a biotinylated photo-cleavable protection group (*red*) and an amino-reactive group (*black*). **b** Schemes of P-Aggs formation. **c** Protein photoliberation from P-Aggs (Figure reproduced with permission from: Ref. [62]. Copyright (2016) with permission from John Wiley and Sons)

**Fig. 5:**
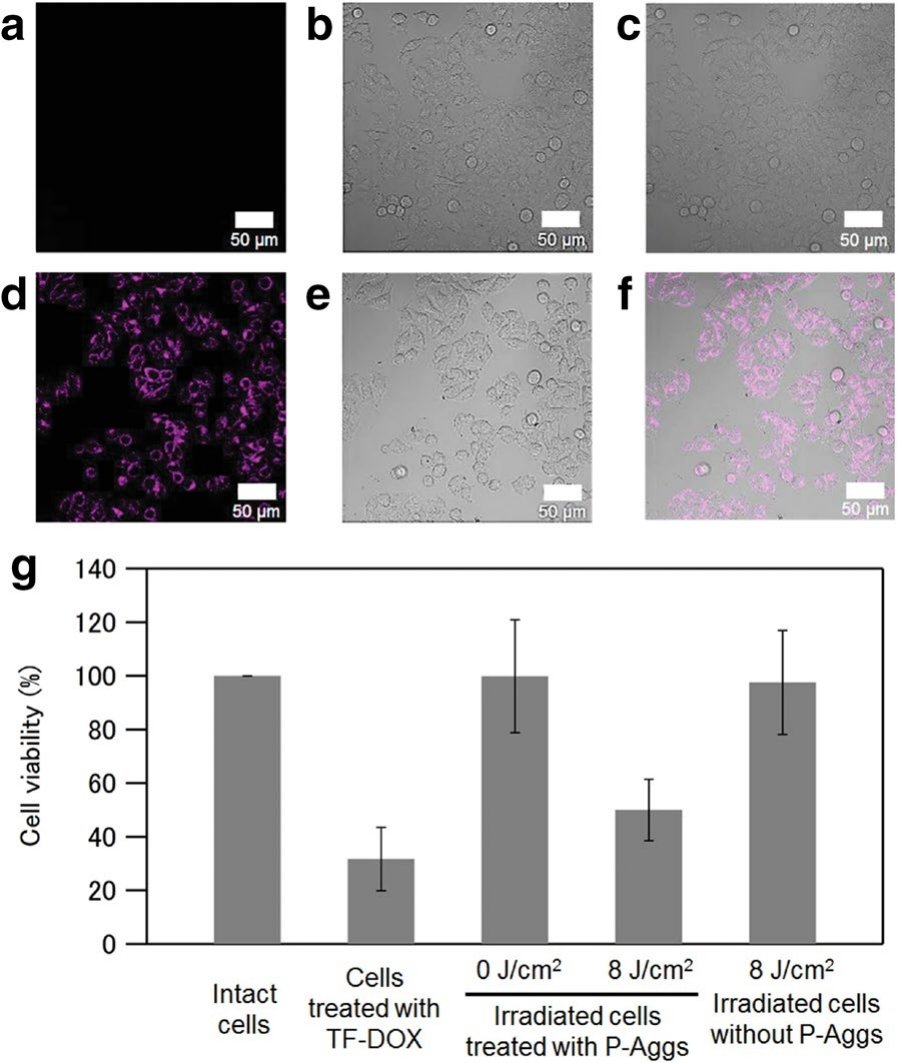
Light-induced cellular uptake of Tf or a chemotherapeutic drug through degradation of P-Aggs. **a**–**c** Confocal microscopy images of DLD1 cells treated with P-Aggs consisting of SA and AF647-labeled caged Tf before light irradiation. **d**–**f** Those after light irradiation at 8 J cm^−2^. **a**, **d** AF647-fluorescence images, **b**, **e** differential interference contrast (DIC) images, **c**, **f** each merged image of (**a**, **b**) or (**d**, **e**), respectively. The *scale bars* are 50 μm. **g** Cell viabilities of the DLD1 cells treated with doxorubicin-modified Tf (Tf-DOX) or with P-Aggs consisting of SA and the caged Tf-DOX before and after light irradiation at 8 J cm^−2^ (Figure reproduced with permission from: Ref. [62]. Copyright (2016) with permission from John Wiley and Sons)

Photolytic protein aggregates (P-Aggs) for light-controllable nanocarriers have also been developed using SA [62]. Submicron-scaled P-Aggs were constructed by mixing SA and cargo proteins labeled with a biotinylated caging reagent (BCR) and were utilized as a facile and versatile platform for the light-induced release of cargo proteins (Fig. 4). The size of P-Aggs could be controlled either by adding an excess of biotin to the above mixture to stop the increase in P-Agg size or by conducting a mixing reaction in a water pool of reverse micelles and adding biotinylated-PEG to stop the increase in P-Agg size. For example, P-Aggs were prepared by mixing SA, a BCR-caged transferrin-doxorubicin conjugate (Tf-DOX) and biotinylated AF647. These P-Aggs multifunctionalized with Tf, Alexa Fluor 647 and DOX were introduced into human colon cancer cells by endocytosis via TfR, followed by the selective release of DOX from the P-Aggs in light-irradiated cells, resulting in the spatiotemporal induction of target cancer cell apoptosis (Fig. 5).

**Fig. 6:**
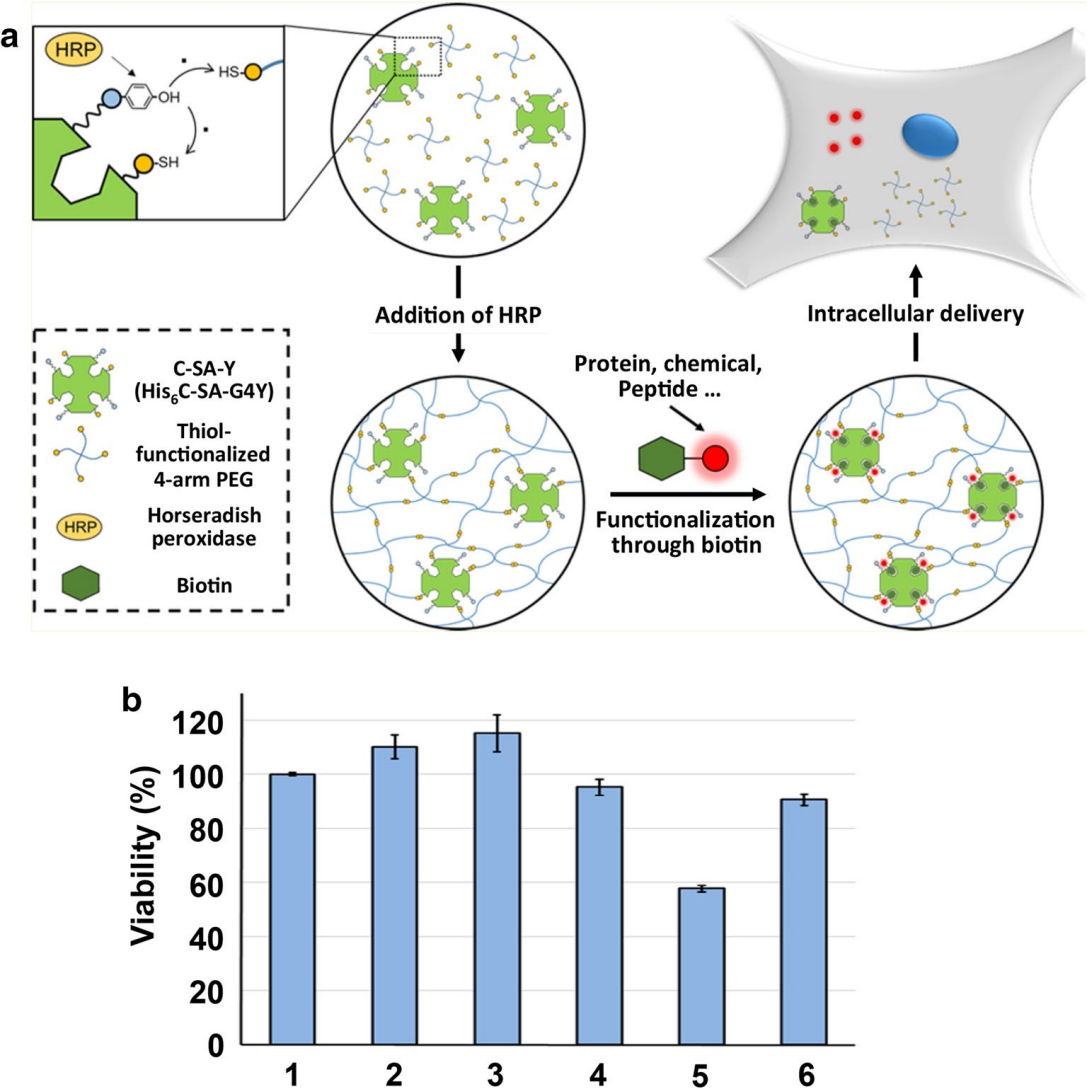
Peptide tag-induced HRP-mediated preparation of a streptavidin-immobilized redox-sensitive hydrogel. **a** Schematic illustration of HRP-mediated preparation of a streptavidin-immobilized redox-sensitive hydrogel and intracellular delivery. **b** Cytotoxicity assay of DLD1 cells incubated with C-SA-Y nanogel functionalized with CPP and saporin. The viability of cells without any treatment was set as 100%. Cells without any treatment (*1*) and treated with C-SA-Y nanogel (*2*), C-SA-Y nanogel with CPP (*3*), C-SA-Y nanogel with saporin (*4*), C-SA-Y nanogel with CPP and saporin (*5*), and saporin (*6*) (Figure reproduced with permission from Ref. [63]. Copyright (2016) with permission from American Chemical Society)

We also developed a method for preparing SA-immobilized redox-sensitive nanohydrogels via peptide tag-induced disulfide formation mediated by horseradish peroxidase (HRP) (Fig. 6a) [63]. In this system, the peptides with sequences of HHHHHHC (C-tag) and GGGGY (Y-tag) were genetically fused to the N- and C-termini of SA (C-SA-Y), respectively. Here, H, C, G and Y denote histidine, cystein, glycine and tyrosine, respectively. The C-SA-Y was mixed with HRP- and thiol-functionalized 4-arm PEG to yield a C-SA-Y-immobilized hydrogel (C-SA-Y gel) crosslinked with redox-sensitive disulfide bonds. The C-SA-Y immobilized in the hydrogel retained its affinity for biotin, allowing the incorporation of any biotinylated functional biomolecules or synthetic chemical agents into the hydrogel via biotin-SA interaction. The C-SA-Y gel was further prepared within a reverse micelle system to yield a nanosized hydrogel, rendering it a potential drug delivery carrier. A C-SA-Y nanogel functionalized with biotinylated CPP (biotin-G3R15GYC-Alexa Fluor 546) and Alexa Fluor 488-labeled saporin was prepared, and we investigated its efficacy as a drug delivery system for human colon adenocarcinoma DLD1 cells (Fig. 6a). The cell viability assay results revealed that the C-SA-Y nanogel prepared without CPP or saporin hardly affected the viability of DLD1 cells. In contrast, treatment of the human colon cancer cells with the C-SA-Y gel functionalized with both CPP and saporin resulted in a marked decrease in cell proliferation (Fig. 6b). These results indicated that the C-SA-Y nanogel had been internalized into the cells via the CPP, reducing cell variability by cytotoxicity of saporin. The internalization ability of CPP and cytotoxicity of saporin were therefore successfully integrated in the C-SA-Y nanogel, with both properties working cooperatively to yield a cytotoxic C-SA-Y nanogel.

### Nanobiomaterials for biosensing and bioanalysis

Biosensing and bioanalysis based on new nanomaterials and nanotechnology in the areas of nanoelectronics, nanooptics, nanopatterns and nanofabrication have a wide range of promising applications in point-of-care diagnostics, earlier disease diagnosis, pathological testing, food testing, environmental monitoring, drug discovery, genomics and proteomics. The rapid development of nanotechnology has resulted in the successful synthesis and characterization of a variety of nanomaterials, making them ideal candidates for signal generation and transduction in sensing. In other words, the unique properties and functionalization of biomaterial-conjugated nanostructures make them very useful for signal amplification in assays, other biomolecular recognition events and fabricating functional nanostructured biointerfaces [64, 65]. Therefore, nanomaterials and nanofabrication technologies play significant roles in fabricating biosensors and biodevices (e.g., colorimetric, fluorescent, electrochemical, surface-enhanced Raman scattering, localized surface plasmon resonance, quartz crystal microbalance and magnetic resonance imaging (MRI)), including implantable devices [66] for the detection of a broad range of biomarkers with ultrahigh sensitivity and selectivity and rapid responses.

#### Nanomaterials for enhancing sensitivity of biosensing and bioanalysis

During the last decade, several promising nanomaterials (e.g., QDs, NPs, carbon nanotubes (CNTs) and graphene) with biomolecule-modified surfaces have been widely used in the fields of biosensing, bioanalysis and diagnostics [67–70]. For example, one of the first nanomaterials to have an impact on amperometric biosensors was CNTs, which have such advantages as a small size with a large surface area, an excellent electron transfer ability, and easy biomolecule immobilization. CNT-modified electrodes improved current densities and enhanced the reactivity of biomolecules, redox cofactors and redox enzymes. In addition, aligned CNT forests facilitated direct electron transfer with the redox centers of enzymes, resulting in improved overall performance of enzyme electrodes and enzyme-labeled immunosensors [71].

Several nanomaterials have shown great promise in imaging due to their intrinsic imaging characteristics, such as their brightness, sharp bandwidth and long-term stability (e.g., fluorescent agents, such as QDs [72], magnetic NPs in MRI [73] and colloidal AuNPs [74]). For imaging, nanomaterials can be targeted to specific disease sites within the body by conjugating the materials to biomarker-specific biomolecules. These biomaterial-based imaging agents can also provide information in addition to anatomical data, e.g., information relating to physiology and function, which enables more accurate and early disease diagnosis, such as the highly sensitive detection of early-stage cancer [75].

#### Nanofabrication technologies for biosensing and bioanalysis

**Fig. 7:**
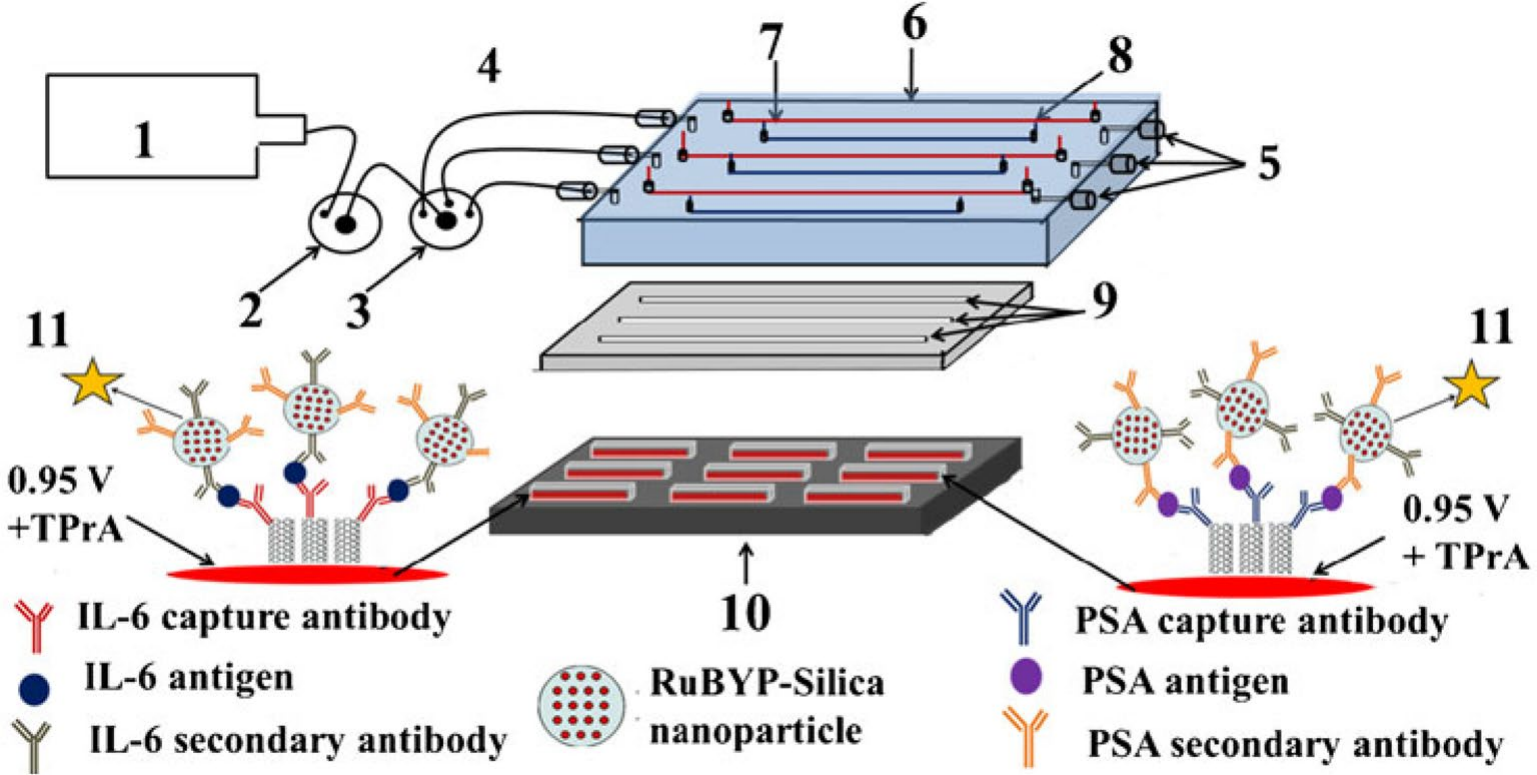
Design of microfluidic ECL array for cancer biomarker detection. (*1*) syringe pump, (*2*) injector valve, (*3*) switch valve to guide the sample to the desired channel, (*4*) tubing for inlet, (*5*) outlet, (*6*) poly(methylmethacrylate) plate, (*7*) Pt counter wire, (*8*) Ag/AgCl reference wire, (*9*) polydimethylsiloxane channels, (*10*) pyrolytic graphite chip (*black*), surrounded by hydrophobic polymer (*white*) to make microwells. Bottoms of microwells (*red rectangles*) contain primary antibody-decorated SWCNT forests, (*11*) ECL label containing RuBPY-silica nanoparticles with cognate secondary antibodies are injected to the capture protein analytes previously bound to cognate primary antibodies. ECL is detected with a CCD camera (Figure reproduced with permission from: Ref. [80]. Copyright (2013) with permission from Springer Nature)

Microarrays [76] and microfluidic [77, 78] platforms coupled with biomolecule-conjugated nanomaterials (e.g., QDs, NPs, or CNTs conjugated with enzymes, antibodies, DNAs, or aptamers) have enabled the simultaneous multiplex detection of many disease biomarkers for cancer, infectious diseases, diabetes, cardiovascular diseases and Alzheimer’s disease. For example, novel electrochemiluminescence (ECL) microwell array [79] and microfluidic [80] immunoassay devices equipped with capture-antibody-decorated single-walled carbon nanotube (SWCNT) forests on pyrolytic graphite chips have been developed. The [Ru(bpy)_3_]^2+^-doped silica NPs covered with thin hydrophilic polymer films prepared by the sequential layer-by-layer deposition of positively charged PDDA and negatively charged PAA were used as ECL labels in these systems for highly sensitive two-analyte detection. Antibodies to prostate specific antigen (PSA) and interleukin (IL)-6 were chemically conjugated to either SWCNTs or polymer-coated RuBPY-silica-Ab_2_ NPs via amidization with 1-(3-dimethylaminopropyl)-3-ethylcarbodiimide hydrochloride (EDC) and *N*-hydroxysulfosuccinimide (NHSS). The microfluidic immunoassay device provided the simultaneous detection of the biomarker proteins PSA and IL-6 in serum, demonstrating high sensitivity and detection limits in the low femtogram per milliliter range (10^−21^ M range) (Fig. 7) [80]. These platforms explored the detection of ultra-low concentrations of target biomarkers and have realized rapid, ultrasensitive and cost-effective bioassays requiring minimum sample volumes, which will enable primary care physicians and patients to perform assays in their respective settings, using so-called point-of-care diagnostics. The detection of cancer biomarkers by immunoassays and sensors using these engineered nanomaterials could also enable the diagnosis of cancer at very early stages [81, 82].

**Fig. 8:**
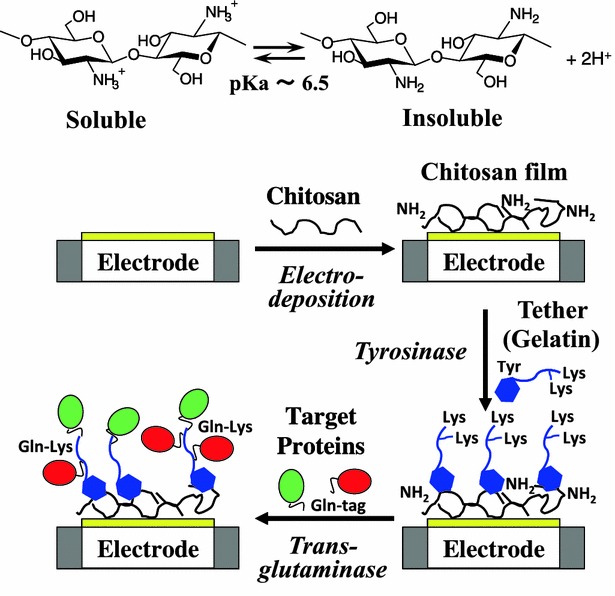
Biofabrication for construction of nanodevices. Schematic of the procedure for orthogonal enzymatic assembly using tyrosinase to anchor the gelatin tether to chitosan and microbial transglutaminase to conjugate target proteins to the tether (Figure adapted with permission from: Ref. [83]. Copyright (2009) American Chemical Society)

Fabrication must employ strategies to control chemistry to ensure not only that patterns and structures are generated at the desired location and within an appropriate time frame but also that undesired side reactions are prevented. Bionanofabrication, the use of biological materials and mechanisms for the construction of nanodevices for biosensing and bioanalysis, offers convergent approaches for building nanointerfaces between biomolecules and devices by either enzymatic assembly or self-assembly. For example, film-forming pH-sensitive chitosan directly assembles on electrodes under physiological conditions in response to electrode-imposed voltages (i.e., electrodeposition). Through recombinant technology, biomolecular engineering allows target proteins to be endowed with peptide tags [e.g., a Glutamine (Gln)-tag for transglutaminase-mediated crosslinking between the side chains of Gln and Lysine (Lys) residues] for assembly, which enables fabrication and controls bioconjugation chemistry through molecular recognition for the enzymatic generation of covalent bonds (Fig. 8) [83]. These self-assembly and enzymatic assembly methods also provide mechanisms for construction over a hierarchy of length scales. Bionanofabrication will enable the effective interfacing of biomolecules with nanomaterials to create implantable devices.

### Nanobiomaterials for biocatalysis

The use of nanomaterials for enzyme immobilization and stabilization is highly effective not only in stabilizing the enzyme activity but also in developing other advantageous properties, including high enzyme loading and activity, an improved electron transfer rate, low mass transfer resistance, high resistance to proteolytic digestion and the easy separation and reuse of biocatalysts by magnetic force [84]. The immobilization or entrapment of enzymes on the surface or interior of nanocarriers has been accomplished using various nanomaterials, such as polymer NPs (e.g., polylactic acid, polystyrene, polyvinyl alcohol, and chitosan), magnetic and superparamagnetic NPs, polymer nanofibers (e.g., nylon, polyurethane, polycarbonate, polyvinyl alcohol, polylactic acid, polystyrene, and carbon), CNTs, GO nanosheets, porous silica NPs, sol–gel NPs and viral NPs [85–87].

#### Enzyme immobilization

There are considerable advantages of effectively immobilizing enzymes for modifying nanomaterial surface properties and grafting desirable functional groups onto their surface through chemical functionalization techniques. The surface chemistry of a functionalized nanomaterial can affect its dispersibility and interactions with enzymes, thus altering the catalytic activity of the immobilized enzyme in a significant manner. Toward this end, much effort has been exerted to develop strategies for immobilizing enzymes that remain functional and stable on nanomaterial surfaces; various methods including, physical and/or chemical attachment, entrapment, and crosslinking, have been employed [86, 88, 89]. In certain cases, a combination of two physical and chemical immobilization methods has been employed for stable immobilization. For example, the enzyme can first be immobilized by physical adsorption onto nanomaterials followed by crosslinking to avoid enzyme leaching. Both glutaraldehyde and carbodiimide chemistry, such as dicyclohexylcarbodiimide/*N*-hydroxysuccinimide (NHS) and EDC/NHS, have been commonly utilized for crosslinking. However, in some cases, enzymes dramatically lose their activities because many conventional enzyme immobilization approaches, which rely on the nonspecific absorption of enzymes to solid supports or the chemical coupling of reactive groups within enzymes, have inherent difficulties, such as protein denaturation, poor stability due to nonspecific absorption, variations in the spatial distances between enzymes and between the enzymes and the surface, decreases in conformational enzyme flexibility and the inability to control enzyme orientation.

**Fig. 9:**
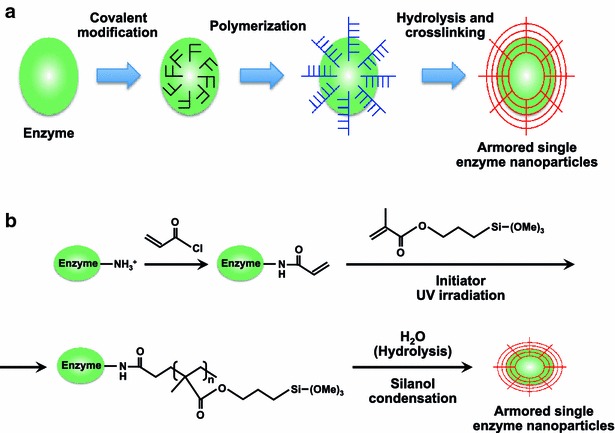
Illustration of armored single-enzyme nanoparticle. **a** Schematic of preparation of the single-enzyme nanoparticles. **b** Chemistry for the synthesis of single-enzyme nanoparticles (Figure adapted with permission from Ref. [90]. Copyright (2003) American Chemical Society)

To overcome these problems, many strategies for enzyme immobilization have been developed. One approach is known as ‘single-enzyme nanoparticles (SENs),’ in which an organic–inorganic hybrid polymer network less than a few nanometers in thickness is built up from the surface of an enzyme. The synthesis of SENs involves three reactions: first, amino groups on the enzyme surface react with acryloyl chloride to yield surface vinyl groups; then, free-radicals initiate vinyl polymerization from the enzyme surface using a vinyl monomer and pendant trimethoxy-silane groups; finally, orthogonal polymerization occurs via silanol condensation reactions to crosslink the attached polymer chains into a network (Fig. 9). It was demonstrated that SENs can be immobilized in mesoporous silica; additionally, this method of immobilization was shown to provide a much more stable immobilized enzyme system than that of native enzymes immobilized by either adsorption or covalent bonding in the same material [90]. Another approach is to introduce molecular interfaces between a solid surface and enzymes. Several methods based on this approach have been reported, such as the surface modification of solid supports with hydrophilic synthetic polymers [91, 92] and peptides [93] with specificities and affinities toward enzymes, and the fusion of enzymes with peptide tags [94] or anchor proteins [95, 96].

Peptides with an affinity for nanomaterials have been identified from a combinatorial peptide library, and these peptides are promising tools for bottom-up fabrication technology in the field of bionanotechnology. Through the use of these peptides, enzymes can be directly immobilized on a substrate surface with desired orientations and without the need for substrate surface modification or complicated conjugation processes. For example, an Au-binding peptide was applied to direct the self-assembly of organophosphorus hydrolase onto an AuNP-coated graphene chemosensor. This electrochemical biosensor system could detect pesticides with a fast response time, low detection limit, better operating stability and high sensitivity [97].

The amphiphilic protein HFBI (7.5 kDa), class II hydrophobin, that is produced by *Trichoderma reesei* adheres to solid surfaces and exhibits self-organization at water–solid interfaces. A fusion protein between HFBI and glucose oxidase (GOx-HFBI) with a 21-AA flexible linker (linker sequence: SGSVTSTSKTTATASKTSTST) was constructed. This fusion protein exhibited the highest levels of both protein adsorption and high GOx activity owing to the presence of the HFBI spacer and flexible linker, which forms a self-organized protein layer on solid surface and enables the GOx component in the fusion protein to be highly mobile, respectively [95].

The crystalline bacterial cell surface layer (S-layer) proteins of prokaryotic organisms constitute a unique self-assembly system that can be employed as a patterning element for various biological molecules, e.g., glycans, polysaccharides, nucleic acids, and lipids. One of the most excellent properties of S-layer proteins is their capability to self-assemble into monomolecular protein lattices on artificial surfaces (e.g., plastics, noble metals or silicon wafers) or on Langmuir lipid films or liposomes. A fusion protein between the S-layer protein SbpA from *Bacillus sphaericus* CCM 2177 and the enzyme laminarinase (LamA) from *Pyrococcus furiosus* fully retained the self-assembly capability of the S-layer moiety, and the catalytic domain of LamA was exposed at the outer surface of the formed protein lattice. The enzyme activity of the S-layer fusion protein monolayer on silicon wafers, glass slides and different types of polymer membranes was compared with that of only LamA immobilized with conventional techniques. LamA aligned within the S-layer fusion protein lattice catalyzed two-fold higher glucose release from the laminarin polysaccharide substrate compared with the randomly immobilized enzyme. Thus, S-layer proteins can be utilised as building blocks and templates for generating functional nanostructures at the meso- and macroscopic scales [98].

#### Multienzyme complex systems

In nature, the macromolecular organization of multienzyme complexes has important implications for the specificity, controllability, and throughput of multi-step biochemical reaction cascades. This nanoscale macromolecular organization has been shown to increase the local concentrations of enzymes and their substrates, to enhance intermediate channeling between consecutive enzymes and to prevent competition with other intracellular metabolites. The immobilization of an artificial multienzyme system on a nanomaterial to mimic natural multienzyme organization could lead to promising biocatalysts. However, the above-mentioned immobilization methods for one type of enzyme on nanomaterials cannot always be applied to multienzyme systems in a straightforward manner because it is very difficult to control the precise spatial placement and the molecular ratio of each component of a multienzyme system using these methods. Therefore, strategies have been developed for the fabrication of multienzyme reaction systems [99, 100], such as genetic fusion [101], encapsulation [102] in reverse micelles, liposomes, nano/mesoporous silica or porous polymersomes, scaffold-mediated co-localization [103], and scaffold-free, site-specific, chemical and enzymatic conjugation [104, 105].

**Fig. 10:**
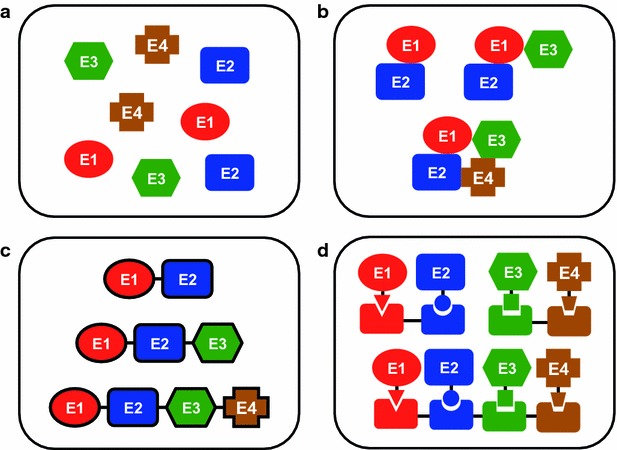
Illustration of different modes of organizing enzyme complexes. **a** Free enzymes, **b** metabolon (enzyme clusters), **c** fusion enzymes, **d** scaffolded enzymes

In many organisms, complex enzyme architectures are assembled either by simple genetic fusion or enzyme clustering, as in the case of metabolons, or by cooperative and spatial organization using biomolecular scaffolds, and these enzyme structures enhance the overall biological pathway performance (Fig. 10) [103, 106, 107]. In metabolons, such as nonribosomal peptide synthase, polyketide synthase, fatty acid synthase and acetyl-CoA carboxylase, reaction intermediates are covalently attached to functional domains or subunits and transferred between domains or subunits. Alternatively, substrate channeling in such multienzyme complexes as metabolons, including by glycolysis, the Calvin and Krebs cycles, tryptophan synthase, carbamoyl phosphate synthetase, and dhurrin synthesis, is utilized to prevent the loss of low-abundance intermediates, to protect unstable intermediates from interacting with solvents and to increase the effective concentration of reactants. Additionally, scaffold proteins are involved in many enzymatic cascades in signaling pathways (e.g., the MAPK scaffold in the MAPK phosphorylation cascade pathway) and metabolic processes (e.g., cellulosomes from *Clostridium thermocellum*). From a practical point of view, there are several obstacles for the genetic fusion of over three enzymes to construct multienzyme complexes. First, large recombinant fusion proteins are easily misfolded and subsequently are either proteolyzed or form inactive inclusion bodies in *E. coli*. Furthermore, the optimum refolding conditions of each enzyme motif in fusion proteins are not always identical. Last, rational design methods for peptide linkers between enzymes that enable control or linker spatial arrangement and orientation have not yet been developed [106]. Additionally, engineering the required interfacial interactions for efficient enzyme clustering is extremely challenging. Therefore, flexible post-translational methods using enzymatic site-specific protein–protein conjugation and synthetic scaffolds by employing orthogonal interaction domains for assembly have been particularly attractive because of the modular nature of biomolecular design [103].

##### Post-translational enzymatic modification-based multienzyme complexes

Many proteins are subjected to post-translational enzymatic modifications in nature. The natural post-translational processing of proteins is generally efficient and site-specific under physiological conditions. Therefore, in vitro and in vivo enzymatic protein modifications have been developed for site-specific protein–protein conjugation. The applications of enzymatic modifications are limited to recombinant proteins harboring additional protein/peptide tags. However, protein assembly using enzymatic modifications (e.g., inteins, sortase A, and transglutaminase) is a promising method because it is achieved simply by mixing proteins without special techniques [106].

**Fig. 11:**
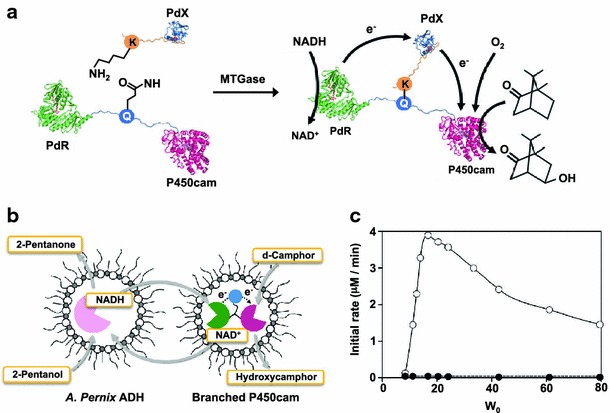
The branched fusion protein construction by MTGase-mediated site-specific protein conjugation. **a** A fusion protein of putidaredoxin reductase (PdR) and P450cam linked with a peptide containing a reactive Gln residue and putidaredoxin attached K-tag generated a three-way branched fusion protein by MTGase. **b** Reaction scheme for d-camphor hydroxylation by branched P450cam with cofactor regeneration in a reversed micellar system. **c** Effect of W_0_ on the initial activities of branched P450cam (*open circles*) and an equimolar mixture of PdR, PdX and P450cam (*closed circles*) (**a** adapted with permission from: Ref. [106]. Copyright (2012) Springer, **b**, **c** adapted with permission from Ref. [109]. Copyright (2010) Oxford University Press)

Recently, we demonstrated a covalently fused multienzyme complex with a “branched structure” using microbial transglutaminase (MTGase) from *Streptomyces mobaraensis,* which catalyzes the formation of an ϵ-(γ-glutamyl) lysine isopeptide bond between the side chains of Gln and Lys residues. A cytochrome P450 enzyme from *Pseudomonas putida (*P450cam) requires two soluble redox proteins, putidaredoxin (PdX) and putidaredoxin reductase (PdR), to receive electrons from NADH for its catalytic cycle, in which PdX reduced by PdR with NADH activates P450cam. Therefore, it has been suggested that the complex formation of P450cam with PdX and PdR can enhance the electron transfer from PdR to PdX and from PdX to P450cam. This unique multienzyme complex with a branched structure that has never been obtained by genetic fusion showed a much higher activity than that of tandem linear fusion P450cam genetically fused with PdX and PdR (Fig. 11a) [108]. This multienzyme complex with a branched structure was further applied to a reverse micelle system. When the solubility of substrate is quite low in an aqueous solution, the reverse micelle system is often adopted for simple, one-step enzymatic reactions because the substrate can be solubilized at a high concentration in an organic solvent, subsequently accelerating the reaction rate. In the case of a multienzyme system, especially systems including electron transfer processes, such as the P450cam system, the reverse micelle system is difficult to apply because each component is usually distributed into different micelles and because the incorporation of all components into the same aqueous pool of micelles is very difficult. Unlike the natural P450cam system, all components of the branched P450cam system were incorporated into the same aqueous pool of micelles at a 1:1:1 ratio (Fig. 11b) and enabled both extremely high local protein concentrations and efficient electron transfer to P450cam, resulting in a reaction activity higher than that of a reverse micelle system composed of an equimolar mixture of PdR, PdX and P450cam (Fig. 11c) [109].

##### Scaffold protein-based multienzyme complexes

Scaffold proteins enable the precise spatial placement of the components of a multienzymatic reaction cascade at the nanometer scale. Scaffolds are involved in many enzymatic reaction cascades in signaling pathways and metabolic processes [110], and they can provide advantages over reactions catalyzed by freely diffusing enzymes by segregating reactions, increasing throughput and providing modularity for the construction of novel reaction networks. Recently, various multienzyme systems have been developed using natural scaffold proteins [111] and synthetic scaffolds [112] composed of elements of natural scaffold proteins, such as cellulosomes [113] and signal transduction scaffolds [114].

**Fig. 12:**
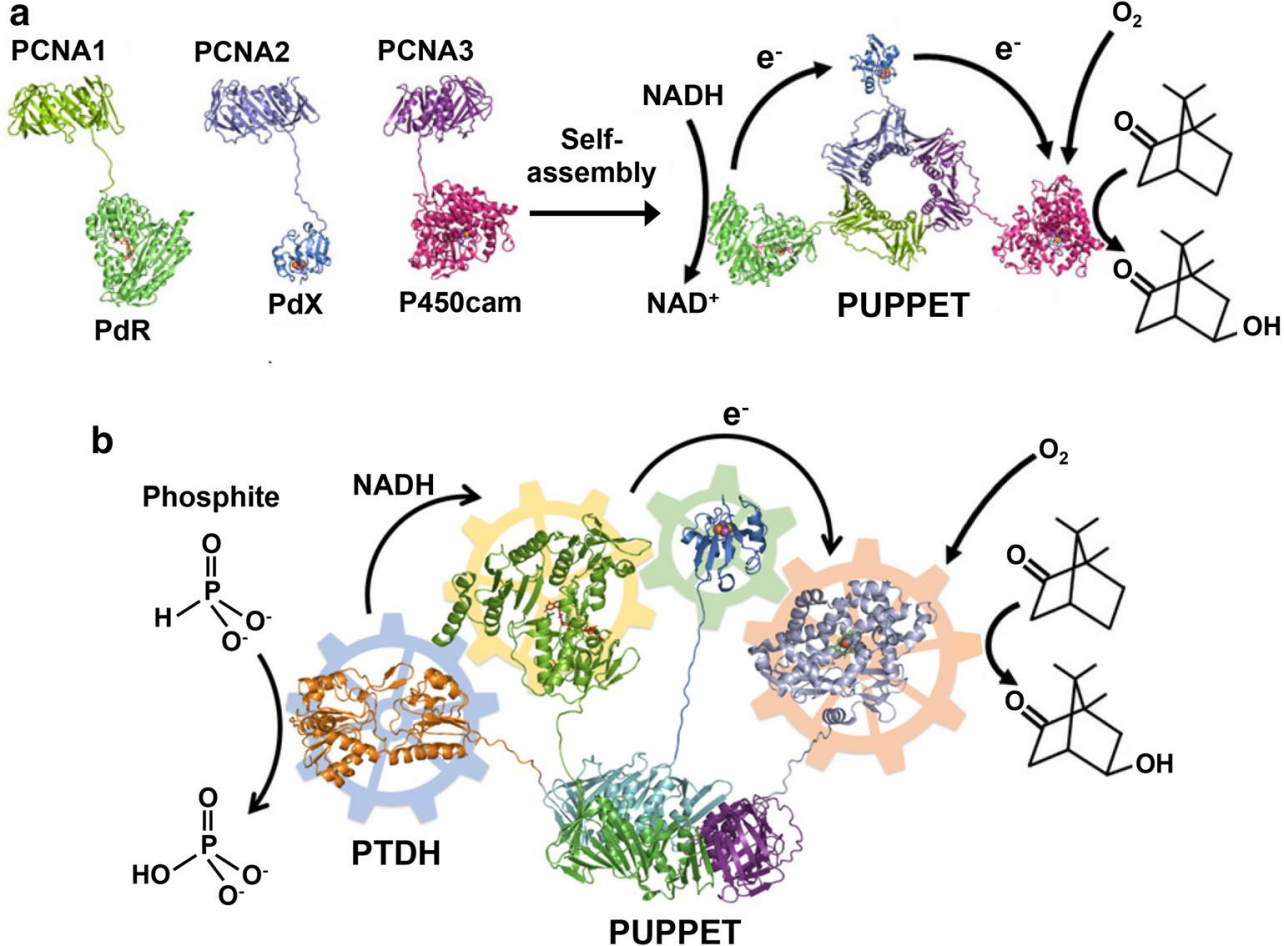
Schematic illustration of PCNA-mediated multienzyme complex formation. **a** Self-assembly of PCNA-based heterotrimeric complex (PUPPET) consisting of P450cam, its electron transfer-related proteins PdR and PdX that catalyzes the hydroxylation of d-camphor. **b** PTDH-PUPPET complex that catalyzes the hydroxylation of d-camphor by regenerating NADH with consumption of phosphite (**a** reproduced with permission from: Ref. [111]. Copyright (2010) Wiley–VCH. **b** Reproduced with permission from: Ref. [115]. Copyright (2013) Wiley–VCH)

Proliferating cell nuclear antigen (PCNA) is a DNA-sliding clamp that forms a symmetrical ring-shaped structure encircling double-stranded DNA (dsDNA) and acts as a scaffold for DNA-related enzymes, such as DNA polymerase and helicase. The archaeon *Sulfolobus solfataricus* has three distinct PCNA genes with the three expressed PCNA proteins, PCNA1, PCNA2 and PCNA3, which form a heterotrimeric complex. These three PCNAs were fused to the three component proteins (i.e., PdR, PdX, and P450cam) composing the *P. putida* P450 system (Fig. 12a). The resulting fusion proteins, PCNA1-PdR, PCNA2-PdX and PCNA3-P450cam, completely retained the functions of the component proteins, including the heterotrimerization of the PCNAs, the catalytic activities of PdR and P450cam, and the electron transfer function of PdX. The three fusion proteins immediately formed a heterotrimeric complex in vitro by mixing. Compared to an equimolar mixture of PdR, PdX and P450cam, the complex showed a 52-fold enhancement in the monooxygenase activity of P450cam because of efficient electron transfer within the complex from PdR to PdX and from PdX to P450cam [111]. This system based on the PCNA scaffold was further extended to a phosphite-driven self-sufficient P450cam system in vitro by incorporating phosphite dehydrogenase (PTDH) for cofactor NADH regeneration (Fig. 12b) [115]. The *K*m value of PTDH-incorporated PUPPET (PTDH-PUPPET) for NAD^+^ (51.0 ± 2.7 μM) in the presence of d-camphor and phosphite was slightly smaller than that of an equimolar mixture of PUPPET and PTDH (69.7 ± 4.8 μM). This result indicates that the oxidation of NADH by the PdR domain in PTDH-PUPPET might increase the effective local concentration of NAD^+^ around the PTDH domain and that this proximity effect on cofactor channeling could potentially be improved by optimizing the arrangement of PTDH and PdR on the PCNA scaffold.

Designer cellulosomes containing four different enzymes (two cellulases and two xylanases) from *Thermobifida fusca* have been reported, where four dockerin-fused cellulolytic enzymes were incorporated into specific locations on an artificial, chimeric scaffold containing four cohesins corresponding to each dockerin. As expected, compared to their free enzyme mixture system without the chimeric scaffolding, the resulting multienzyme complexes exhibited enhanced activity (~2.4-fold) on wheat straw as a complex cellulosic substrate [116].

Recently, Deuber et al. demonstrated in vivo multienzyme complex formation in *E. coli* cells via synthetic protein scaffold expression. Protein scaffolds with various arrangements of fusion domains were built from the interaction domains of signaling proteins, the mouse SH3 and PDZ domains and the rat GTPase protein-binding domain (GBD). The three enzymes acetoacetyl-CoA thiolase, hydroxymethylglutaryl-CoA synthase and hydroxymethylglutaryl-CoA reductase, which catalyze a cascade reaction from acetyl-CoA to mevalonate, were genetically tagged with their cognate peptidyl ligands. These protein scaffolds and enzymes with peptidyl ligands were co-expressed in *E. coli* cells. A significant 77-fold increase in mevalonate production was achieved by the expression of the optimized scaffold: (GBD)_1_-(SH3)_2_-(PDZ)_2_ [114].

##### Oligonucleotide scaffold-based multienzyme complexes

DNA has numerous attractive features as a scaffold for multienzyme complexes. Its properties, such as high rigidity, programmability, complexity and assembly through complementary hybridization, allow DNA to form excellent scaffolds with linear, two-dimensional (2D) and 3D structures (e.g., simple dsDNA helices, Holliday junctions, DNA tiles, and DNA origami) for arranging multiple enzymes with controlled spacing in linear, 2D or 3D geometric patterns and for constructing interactive multienzyme complexes and networks [117–120]. DNA–protein conjugates are necessary to achieve DNA-directed protein assembly for the fabrication of multienzyme complexes on DNA scaffolds. However, this requirement makes it difficult to utilize this assembly method in vivo. Currently, there are several methodologies for conjugating proteins with DNA [117]. Proteins have been assembled onto DNA scaffolds through intervening adapter molecules, such as biotin–streptavidin, Ni–NTA-hexahistidine, antibodies-haptens and aptamers. Alternatively, direct covalent conjugation with DNA can be achieved by modifying cysteine (Cys) or Lys residues via disulfide or maleimide coupling, as well as by bioorthogonal chemistry, such as expressed protein ligation, Staudinger ligation and Huisgen cycloaddition.

By utilizing DNA nanostructures as assembly scaffolds, it has become feasible to organize the DNA-directed assembly of artificial multienzyme complexes. DNA-mediated assembly was employed to control the activity of a multidomain enzyme. Cytochrome P450 BM3 (P450 BM3) is composed of two domains, a flavin adenine dinucleotide and flavin mononucleotide-containing reductase domain (BMR) and a heme-containing monooxygenase domain (BMP). P450 BM3 shows monooxygenase activity by transferring electrons to BMP from NADPH through BMR. Both subdomains were genetically fused to the HaloTag protein, a self-labeling enzyme, enabling bioconjugation with chloroalkane-modified DNAs and subsequently reconstituting BM3 activity by DNA-mediated assembly. The arrangement of the two domains on a DNA scaffold can control the distance between them. The distance-dependent activity of multidomain P450 BM3 complexes was investigated by varying the length of spacing scaffolds between the BMR and BMP domains. The resulting changes in distance between the redox centers of the two domains regulated the efficiency of electron transfer and thus the enzymatic activity of the reconstituted P450 BM3 [121].

2D DNA nanostructures provide an even greater opportunity to organize multienzyme systems into more complicated geometric patterns. Thiolated nucleic acids were covalently linked to glucose oxidase (GOx) and horseradish peroxidase (HRP) by using *N*-[(1-maleimidocapropyloxy)sulphosuccinimide ester] as a bifunctional crosslinker. The GOx/HRP enzyme cascade was organized on 2D hexagonal DNA strips via self-assembly. The distance between two enzymes was controlled by varying the positions of two free DNA tethers on the hexagonal DNA strips. The complementary DNA-conjugated enzymes organized on the two-hexagon strips (shorter distances) showed 1.2-fold higher activity than the four-hexagon strips. With shorter distances, intermediate (H_2_O_2_) diffusion was more efficient, which therefore resulted in increased cascade reaction efficiency. However, the enzyme cascade was not activated in the absence of the DNA scaffolds or in the presence of foreign DNA [122]. These observations indicate that spatial arrangement at the nanometer scale using a 2D nanostructure comprising a rigid DNA duplex could control the flux of an intermediate from a primary enzyme to a secondary enzyme and that the flux control dominated the multienzyme cascade reaction rate.

**Fig. 13:**
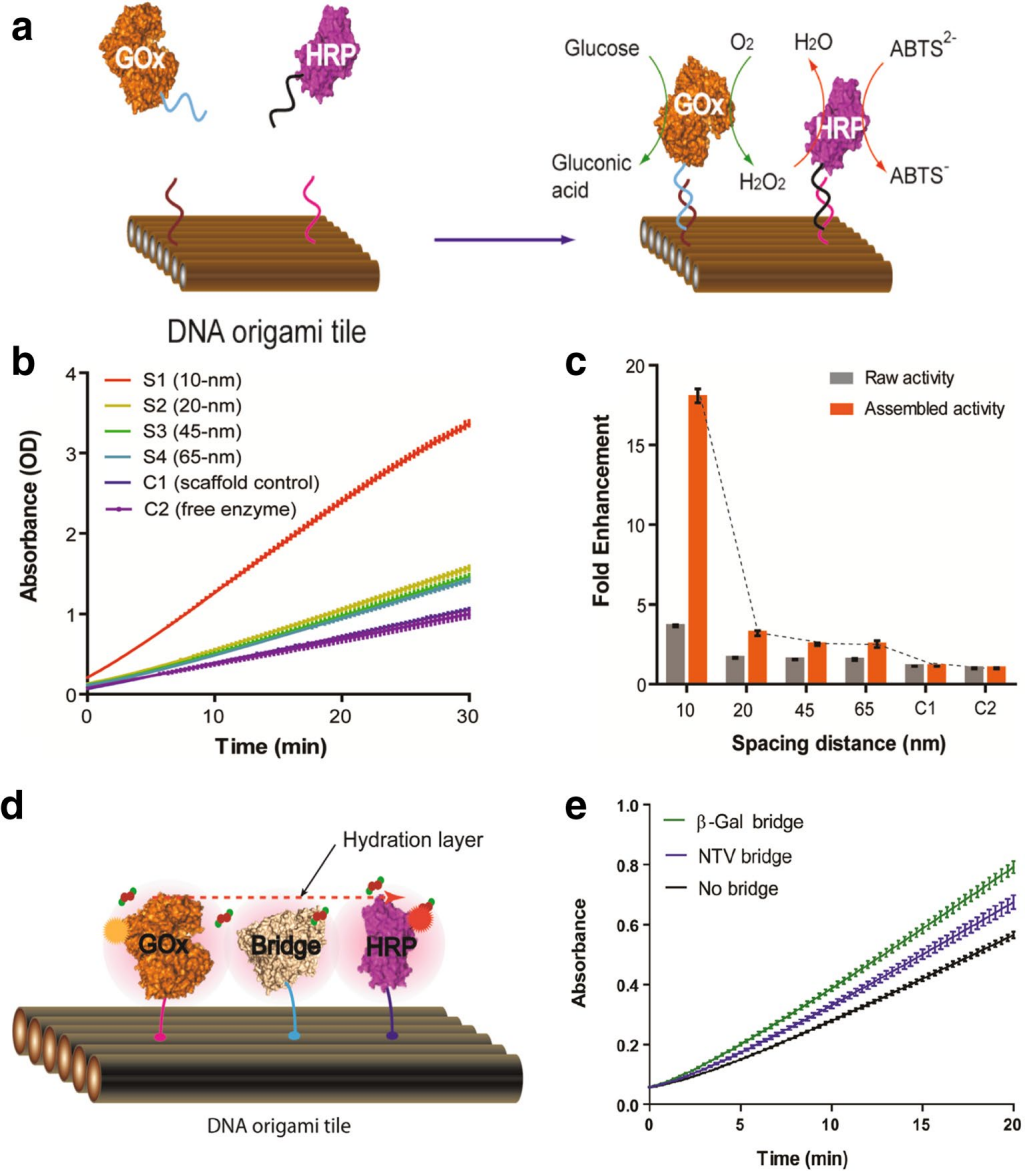
Schematic illustration of interenzyme substrate diffusion for an enzyme cascade organized on spatially addressable DNA nanostructures. **a** DNA nanostructure-directed coassembly of GOx and HRP enzymes with control over interenzyme distances and details of the GOx/HRP enzyme cascade. **b** Spacing distance-dependent effect of assembled GOx/HRP pairs as illustrated by plots of product concentration (Absorbance of ABTS^−^) vs time for various nanostructured and free enzyme samples. **c** Enhancment of the activity of the enzyme pairs on DNA nanostructures compared to free enzyme in solution. **d** The design of an assembled GOx/HRP pair with a protein bridge used to connect the hydration surfaces of GOx and HRP. **e** Enhancement in the activity of assembled GOx/HRP pairs with β-Gal and NTV bridges compared to unbridged GOx/HRP pairs (Figure reproduced with permission from: Ref. [123]. Copyright (2012) American Chemical Society)

More accurate distance control of the GOx/HRP enzyme cascade was realized using DNA origami tiles as a scaffold. The distance between enzymes was systematically varied from 10–65 nm, and the corresponding activities were evaluated. The study revealed the existence of two different distance-dependent kinetic processes associated with the assembled enzyme pairs. Strongly enhanced activity was observed when the enzymes were closely spaced, while the activity decreased drastically for enzymes as little as 20 nm apart. Increasing the spacing further showed much weaker distance dependence (Fig. 13a–c). This study revealed that intermediate transfer between enzymes might occur at the connected hydration shells for closely spaced enzymes. This mechanism was verified by constructing different sizes of noncatalytic protein bridges (β-galactosidase (β-Gal) and NeutrAvidin (NTV)) between GOx and HRP to facilitate intermediate transfer across protein surfaces. The bridging protein changed the Brownian diffusion, resulting in the restricted diffusion of H_2_O_2_ along the hydration layer of the contacted protein surfaces and enhancing the enzyme cascade reaction activity (Fig. 13d, e) [123].

An enzyme cascade nanoreactor was constructed by coupling GOx and HRP using both a planar rectangular orientation and short DNA origami NTs. Biotinylated GOx and HRP were positioned on the streptavidin-decorated planar rectangular DNA sheet via the biotin–avidin interaction with a specific interenzyme distance (i.e., the distance between GOx and HRP) of 15 nm. This DNA sheet equipped with GOx and HRP was then rolled into a confined NT, resulting in the encapsulation of the enzymes in a nanoreactor. Remarkably, the enzymatic coupling efficiency of this enzyme cascade within short DNA NTs was significantly higher than that on the planar rectangular DNA sheet alone. When both enzymes were confined within the DNA NTs, H_2_O_2_ could not diffuse out of the diffusion layer, which was much thicker than the diameter of the DNA NTs (20 nm), resulting in a high coupling of the reaction intermediate H_2_O_2_ between the enzymes [124].

A similar modular type of enzyme cascade nanoreactor was constructed using 3D DNA origami building blocks. Each of the DNA origami units contained three biotin-conjugated strands protruding from the inner surface of the tubular structure. The deglycosylated avidin and NTV were immobilized on the inner surface of the units via the biotin–avidin interaction to facilitate the further binding of biotinylated enzymes. Biotinylated GOx and HRP were anchored inside the origami compartment with the help of NTV. The resulting GOx- and HRP-immobilized tubular DNA origami structures were connected together by hybridizing 32 short (3–6 bases) sequences. The GOx/HRP cascade reaction of the assembled dimer nanoreactor showed significantly higher activity than that without a DNA scaffold [125].

Engineered RNA modules were assembled into discrete (0D), one-dimensional (1D) and 2D scaffolds with distinct protein-docking sites (duplexes with aptamer sites) and used to control the spatial organization of a hydrogen-producing pathway in bacteria. The 0D, 1D and 2D RNA scaffolds were assembled in vivo through the incorporation of two orthogonal aptamers for capturing the target phage-coat proteins MS2 and PP7. Cells expressing the designed RNA scaffold modules and both ferredoxin/MS2 (F_M_) and [FeFe]-hydrogenase/PP7 (H_P_) fusion proteins showed remarkable increases in hydrogen production. Namely, 4-, 11- and 48-fold enhancements in hydrogen production compared with that of control cells were observed from the RNA-templated hydrogenase and ferredoxin cascade reactions in cells expressing 0D, 1D and 2D RNA scaffolds, respectively. This study suggests that a metabolic engineering approach can be used to introduce structural nucleic acid nanostructures inside cells for the organization of multienzyme reaction pathways [126].

## Biomolecular engineering for nanobio/bionanotechnology

Biomolecular engineering addresses the manipulation of many biomolecules, such as nucleic acids, peptides, proteins, carbohydrates, and lipids. These molecules are the basic building blocks of biological systems, and there are many new advantages available to nanotechnology by manipulating their structures, functions and properties. Since every biomolecule is different, there are a number of technologies used to manipulate each one individually.

**Fig. 14:**
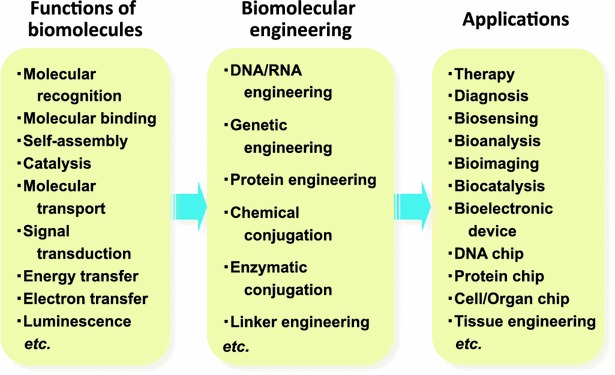
Overview of biomolecular engineering for enhancing, altering and multiplexing functions of biomolecules, and its application to various fields

Biomolecules have various outstanding functions, such as molecular recognition, molecular binding, self-assembly, catalysis, molecular transport, signal transduction, energy transfer, electron transfer, and luminescence. These functions of biomolecules, especially nucleic acids and proteins, can be manipulated by nucleic acid (DNA/RNA) engineering, gene engineering, protein engineering, chemical and enzymatic conjugation technologies and linker engineering. Subsequently, engineered biomolecules can be applied to various fields, such as therapy, diagnosis, biosensing, bioanalysis, bioimaging, and biocatalysis (Fig. 14).

### Nucleic acid engineering

Nucleic acids, such as DNA and RNA, exhibit a wide range of biochemical functions, including the storage and transfer of genetic information, the regulation of gene expression, molecular recognition and catalysis. Nucleic acid engineering based on the base-pairing and self-assembly characteristics of nucleic acids is key for DNA/RNA nanotechnologies, such as those involving DNA/RNA origami, aptamers, and ribozymes [16, 17, 127].

#### DNA/RNA origami

DNA/RNA origami, a new programmed nucleic acid assembly system, uses the nature of nucleic acid complementarity (i.e., the specificity of Watson–Crick base pairing) for the construction of nanostructures by means of the intermolecular interactions of DNA/RNA strands. 2D and 3D DNA/RNA nanostructures with a wide variety of shapes and defined sizes have been created with precise control over their geometries, periodicities and topologies [16, 128, 129]. Rothemund developed a versatile and simple ‘one-pot’ 2D DNA origami method named ‘scaffolded DNA origami,’ which involves the folding of a long single strand of viral DNA into a DNA scaffold of a desired shape, such as a square, rectangle, triangle, five-pointed star, and even a smiley face using multiple short ‘staple’ strands [130]. To fabricate and stabilize various shapes of DNA tiles, crossover motifs have been designed through the reciprocal exchange of DNA backbones. Branched DNA tiles have also been constructed using sticky ends and crossover junction motifs, such as tensegrity triangles (rigid structures in a periodic-array form) and algorithmic self-assembled Sierpinski triangles (a fractal with the overall shape of an equilateral triangle). These DNA tiles can further self-assemble into NTs, helix bundles and complex DNA motifs and arrays [17]. 3D DNA origami structures can be designed by extending the 2D DNA origami system, e.g., by bundling dsDNAs, where the relative positioning of adjacent dsDNAs is controlled by crossovers or by folding 2D origami domains into 3D structures using interconnection strands [131]. 3D DNA networks with such topologies as cubes, polyhedrons, prisms and buckyballs have also been fabricated using a minimal set of DNA strands based on junction flexibility and edge rigidity [17].

Because the folding properties of RNA and DNA are not exactly the same, the assembly of RNA was generally developed under a slightly different perspective due to the secondary interactions in an RNA strand. For this reason, RNA tectonics based on tertiary interactions have been introduced for the self-assembly of RNA. In particular, hairpin–hairpin or hairpin–receptor interactions have been widely used to construct RNA structures [16]. However, the fundamental principles of DNA origami are applicable to RNA origami. For example, the use of three- and four-way junctions to build new and diverse RNA architectures is very similar to the branching approaches used for DNA. Both RNA and DNA can form jigsaw puzzles and be developed into bundles [17].

One of the most important features of DNA/RNA origami is that each individual position of the 2D structure contains different sequence information. This means that the functional molecules and particles that are attached to the staple strands can be placed at desired positions on the 2D structure. For example, NPs, proteins or dyes were selectively positioned on 2D structures with precise control by conjugating ligands and aptamers to the staple strands. These DNA/RNA origami scaffolds could be applied to selective biomolecular functionalization, single-molecule imaging, DNA nanorobot, and molecular machine design [131]. The potential use of DNA/RNA nanostructures as scaffolds for X-ray crystallography and nanomaterials for nanomechanical devices, biosensors, biomimetic systems for energy transfer and photonics, and clinical diagnostics and therapeutics have been thoroughly reviewed elsewhere [16, 17, 127–129]; readers are referred to these studies for more detailed information.

#### Aptamers

**Fig. 15:**
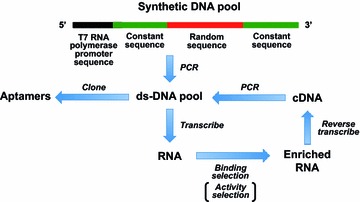
The general procedure for the in vitro selection of aptamers or ribozymes

Aptamers are single-stranded nucleic acids (RNA, DNA, and modified RNA or DNA) that bind to their targets with high selectivity and affinity because of their 3D shape. They are isolated from 10^12^ to 10^15^ combinatorial oligonucleotide libraries chemically synthesized by in vitro selection [132]. Many protocols, including high-throughput next-generation sequencing and bioinformatics for the in vitro selection of aptamers, have been developed and have demonstrated the capacity of aptamers to bind to a wide variety of target molecules, ranging from small metal ions, organic molecules, drugs, and peptides to large proteins and even complex cells or tissues [39, 133–136]. The general in vitro selection procedure for an aptamer, SELEX (Fig. 15), is as follows: a synthetic DNA pool is prepared by chemical synthesis. DNAs consist of a random or mutagenized sequence region flanked on each end by a constant sequence and with a T7 RNA polymerase promoter at the 5′ end. This DNA is amplified by a few cycles of polymerase chain reaction (PCR) and subsequently transcribed in vitro to make the RNA pool. The RNA molecules are then selcted based on their binding affinity to the target molecule, for example, by passing them through a target-immobilized affinity column. The retained RNAs are eluted, reverse transcribed, amplified by PCR, and transcribed; then, the entire cycle is repeated. After multiple rounds of selection (generally 6–18 rounds), quite large populations (>10^13^ different sequences) can be sieved, the ratio of active-to-inactive RNA sequences increases and finally the pool becomes dominated by molecules that can bind the target molecule.

Chemically modified nucleotides provide several advantages, such as enhanced nuclease resistance, an improved binding affinity, increased oligonucleotide pool diversity and improved success rate of selection. Therefore a modified oligonucleotide pool is becoming more popular for aptamer selection. Although chemically modified nucleotides and deoxynucleotide triphosphates cannot be recognized by wild-type T7 RNA polymerases and A-type DNA polymerases, such as *Taq* polymerase, fortunately, modified nucleotide triphosphates (2′-fluoro pyrimidines, 2′-*O*-methyl nucleotides) and functionalized 2′-deoxynucleotide triphosphates with AA-like residues (e.g., indole, benzyl, or alkyne moieties) can be recognized by some mutant RNA polymerases [137] and B-type polymerases, and *Pwo* and Vent (exo-) DNA polymerases [138], respectively.

Specific aptamers against diverse targets have been developed and aptamer-conjugated nanomaterials such as drug-encapsulated polymer NPs, CNTs, AuNPs, QDs and DNA origami demonstrated potential in applications ranging from therapy, targeted drug delivery, sensors and diagnostic reagents to aptamer-directed protein arrays on DNA nanostructures. The details of such applications will not be covered in this review; readers are referred to several recently published reviews [29–31, 40, 64, 68, 132, 139, 140].

#### Ribozymes

Natural ribozymes are RNA molecules that have enzymatic activity for cleaving phosphodiester linkages. Therefore, ribozymes have significant potential for use in cancer, genetic disease, and viral therapeutics by specifically inhibiting gene expression through cleaving RNA substrates, such as mRNA, with the viral genome of RNA containing a sequence complementary to the catalytic center of the ribozymes [141].

Natural ribozymes bind to substrate RNAs through Watson–Crick base pairing, which offers the sequence-specific cleavage of substrate RNAs. Two ribozymes, the ‘hammerhead’ ribozyme and the ‘hairpin’ ribozyme, have been extensively studied [142]. The catalytic motif of a ribozyme is surrounded by a flanking sequence that is responsible for ‘guiding’ the ribozyme to its target RNA and giving stability to the structure. With the hammerhead ribozyme, cleavage is dependent on divalent metal ions, such as magnesium, and can occur after any NUH triplet (where N = any nucleotide and H = A, C or U) within the target RNA sequence. The kinetics of the reaction can vary significantly (up to one or more orders of magnitude) with different triplet-flanking sequence combinations; thus, the choice of an appropriate ribozyme cleavage site is the first and most important step in hammerhead ribozyme design [143].

Artificial ribozymes with catalytic properties have been isolated by in vitro selection from random or combinatorial nucleic acid libraries. Variations of the aptamer selection strategies can be used to isolate catalytic nucleic acid sequences by changing the binding selection step of the aptamer selection process to an activity selection step (Fig. 15). Such approaches have been used to change the function of known ribozymes and to create completely new ones from a random or combinatorial nucleic acid pool [144]. A broad range of chemical reactions could be catalyzed, such as the formation, cleavage and rearrangement of various types of covalent bonds. Examples including not only the cleavage or ligation of RNA substrates by phosphoester transfer at the phosphorus center [144, 145] but also Diels–Alder reactions, N-glycosidic bond formation, alkylations, acylations, and amide bond formations at the carbon centers [144, 146] have been reviewed. The catalytic performance, nuclease resistance and diversity of the oligonucleotide pools of ribozymes could also be enhanced by the incorporation of chemically modified nucleotides, as utilized in aptamer selection protocols [146].

Ribozymes can be expressed from a vector, which offers the advantage of the continued intracellular production of these molecules. However, the turnover rates of ribozymes are rather low in some cases, since dissociation from the cleavage product is the rate-limiting step that controls their usefulness. Furthermore, some ribozymes require high divalent metal ion concentrations for efficient substrate cleavage, which may limit their use in intracellular environments. All of these concerns, as well as off-target activity, resistance to serum and cellular nucleases, and cell-specific, targeted delivery, need to be addressed and overcome in order to utilize ribozymes in therapies. Ribozymes can be hardly incorporated into cells in their naked forms and often required a vehicle for efficient delivery. Many classes of nanomaterials including cationic liposomes, cationic polymer micelles [147] and spherical nucleic acids composed of inorganic core and densely packed, highly oriented nucleic acid shell [148] have been used as delivery vehicles to prevent nuclease-dependent degradation and to enhance cell-targeting and intracellular transduction [143].

### Gene engineering

Gene engineering is a powerful tool for creating artificial genes for proteins and enzymes with desired, improved and multiple properties such as molecular recognition, molecular binding, self-assembly, catalysis, molecular transport, signal transduction, energy transfer, electron transfer, and luminescence, which contribute to develop novel nanobiomaterials, nanobiodevices and nanobiosystems. This technology has been employed to evolve genes in vitro through an iterative process consisting of recombinant generation. Coupled with the powerful HTS or selection methods, gene engineering has been widely applied to solve problems in protein engineering. This technology includes technologies for direct gene manipulation, such as gene mutagenesis, DNA sequence amplification [e.g., PCR and rolling circle amplification (RCA)], DNA shuffling and gene fusion.

There are many methods to generate genetic diversity and to create combinatorial libraries. For example, a variety of in vitro gene manipulation techniques have been developed over the past decade that allow various types of directed changes in a gene by modifying (inserting, deleting or replacing) one or more codons (gene mutagenesis), swapping domains between related functional gene sequences (DNA shuffling) and fusing domains from different functional gene sequences (gene fusion), resulting in the creation of diverse collections of mutant gene clones. There are two main types of mutagenesis, i.e., random and site-directed mutagenesis.

#### Random mutagenesis

With random mutagenesis, point mutations are introduced at random positions in a gene of interest, typically through error-prone PCR mutagenesis, in which MnCl_2_ is added to the reaction mixture to cause a reduction in the fidelity of the DNA amplification [149]. The modified error-prone PCR method, which achieves higher frequencies of base substitutions and both transition and transversion mutations, was developed using mixtures of triphosphate derivatives of nucleoside analogs [150, 151]. An error-prone RCA method, which is an isothermal DNA amplification method with the addition of MnCl_2_ to the reaction mixture, was also developed for random mutagenesis [152]. Different in vitro chemical mutagenesis methods have also been used to introduce random mutations into a gene of interest. In these methods, bases of DNA are modified by chemical mutagens, such as nitrous acid, bisulfate, hydroxylamine and ethyl methane sulfonate, and these methods have less bias than does mutagenesis using PCR-based methods [153]. Randomized sequences are then cloned into a suitable expression vector, and the resulting mutant libraries can be screened to identify mutants with altered or improved properties.

#### Site-directed mutagenesis

Site-directed mutagenesis is a method for altering a gene sequence at a selected location by using overlapping extension PCR. Point mutations, insertions, or deletions are introduced by incorporating DNA primers containing the desired modification with a DNA polymerase in an amplification reaction. Site-saturation mutagenesis further allows the substitution of predetermined protein sites against all twenty possible AAs at once by employing degenerate primers in which the three bases of the targeted codon are replaced by mixtures, most commonly NNN or NNK (N = A, C, G or T; K = G or T). A completely randomized codon, NNN, results in a library size of 64 different sequences encoding all 20 AAs and 3 stop codons. On the other hand, NNK codons reduce the library size by half, still encoding 20 AAs, with the advantage of having only one stop codon. In this configuration, the AAs W, F, I, Y, Q, N, H, K, D, E, M and C are encoded by a single codon, while G, A, V, P, and T, and L, S, and R are encoded by two and three codons, respectively [154].

#### DNA shuffling

**Fig. 16:**
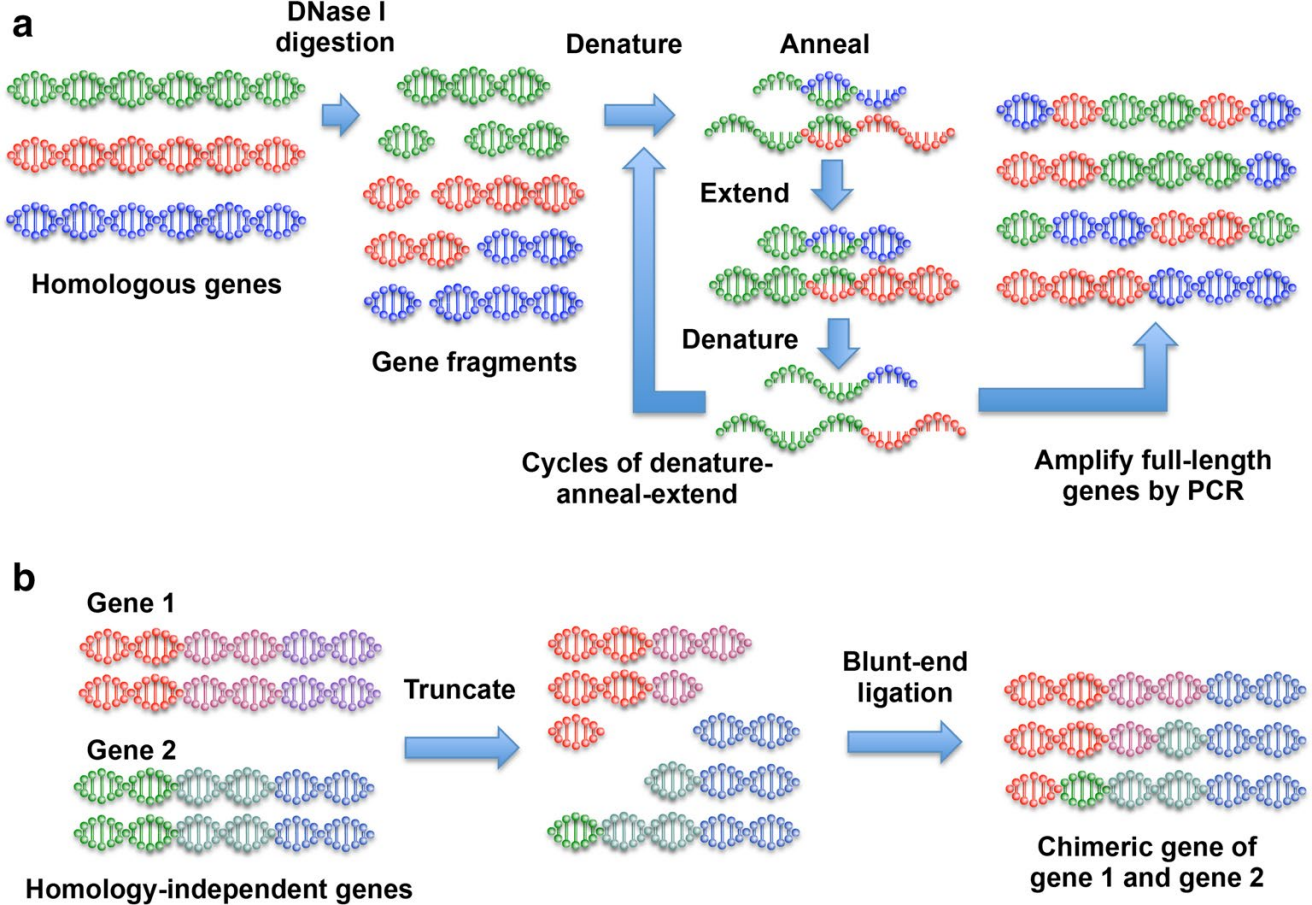
Illustrations of genetic recombination methods for protein evolution. **a** DNA shuffling (in vitro recombination of homologous genes). **b** ITCHY (in vitro recombination of homology-independent genes) (Figure adapted from Ref. [172])

DNA shuffling is a method for the in vitro recombination of homologous genes to quickly generate a large library of chimeric progeny genes incorporating sequence fragments from a number of parent genes by random fragmentation though DNase I and PCR extension without primers for reassembly; this process is followed by PCR amplification with primers to generate full-length chimeras suitable for cloning into an expression vector (Fig. 16a) [155]. One significant drawback of this DNA-shuffling method is the low frequency of chimeric genes in the shuffled library, which may be due to the homo-duplex formation of DNA fragments derived from the same parental genes at the annealing step, the probability of which is much higher than that of hetero-duplex formation. To address this problem, a modified DNA-shuffling method can be used; this method involves the fragmentation of the parental genes using restriction enzymes rather than DNase I [156] or uses single-stranded DNA (ssDNA) templates rather than dsDNA templates for DNase I fragmentation [157]. Since the use of ssDNA as templates will decrease the probability of homo-duplex formation, the percentage of the parental genes in the shuffled library should be significantly reduced.

DNA shuffling has been extended to distantly or completely unrelated gene families, which require methods that do not rely on homologous recombination because of the degree of sequence divergence. Sequence homology-independent protein recombination [158] and incremental truncation for the creation of hybrid enzymes lead to the formation of chimeric genes (Fig. 16b) [159]. The rearrangement of these chimeras by shuffling yields functional hybrids [160]. The main advantage of these methods is that knowledge about detailed protein structure is not required [161].

Exon shuffling is a natural molecular mechanism for the formation of new eukaryotic genes. New exon combinations can be generated by recombination within the intervening intron sequences, yielding new rearranged genes with altered functions. The natural process of exon shuffling can be mimicked in vitro by generating libraries of exon-shuffled genes and subsequently screening target DNA from libraries [162]. In this method, exons or combinations of exons that encode protein domains are amplified by PCR using mixtures of chimeric oligonucleotides that determine which exons are spliced together. By means of a self-priming overlap polymerase reaction, mixtures of these PCR fragments are combinatorially assembled into full-length genes. Recombination is performed by connecting an exon from one gene to an exon from a different gene. In this way, two or more exons from different genes can be combined together ectopically, or the same exon can be duplicated, to create a new exon–intron structure.

#### Gene fusion

Fusion genes are created by genetically fusing the open reading frames of two or more genes in-frame through ligation or overlap extension PCR. To construct such fusion genes, two types of connection are possible. One is ‘end-to-end’ fusion, in which the 5′ end of one gene is linked to the 3′ end of the other gene. The second is insertional fusion, in which one gene is inserted in-frame into the middle of the other parent gene [163]. These methods offer various advantages for producing fusion genes with high throughput in different orientations and including linker sequences to maximize the performance of fusion partners [164].

### Protein engineering

**Fig. 17:**
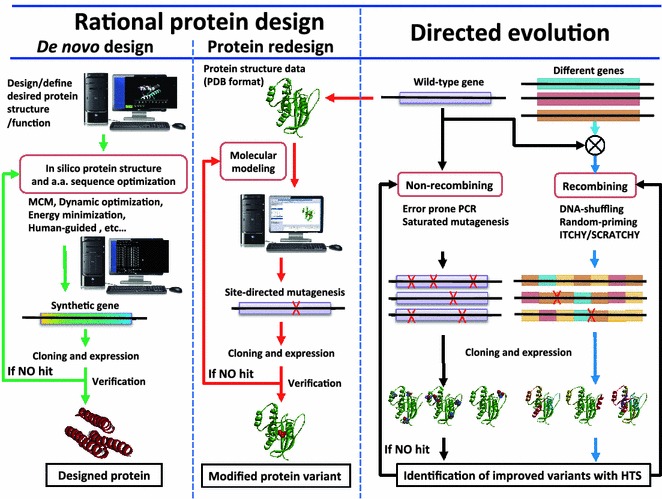
Two general strategies and their procedures for protein engineering

The field of protein engineering has always played a central role in biological science, biomedical research, and biotechnology. Protein engineering is also indispensable technology to design useful and valuable building blocks for nanobio/bionanotechnology to fabricate a variety of artificial self-assembled protein systems with nanoscale structures [18, 19], proteins with tagged peptides for immobilization on NPs [94] and engineered proteins for applications to bioelectronic devices [23, 26, 27], therapy [42, 44, 45, 67, 165], bioimaging [67, 166], biosensing [83, 97, 167], and biocatalysis [87, 89, 95, 98, 101, 103, 108, 110–116]. There are two general strategies for protein engineering, i.e., rational protein design and directed evolution (high-throughput library screening- or selection-based approaches) (Fig. 17).

#### Rational protein design

In rational protein design (Fig. 17, the left panel), detailed knowledge of the structure and function of a protein is used to make desired changes to the protein. In general, this approach has the advantage of creating functionally improved proteins easily and inexpensively, since site-directed mutagenesis techniques allow precise changes in AA sequences, loops and even domains in proteins [161]. However, the major drawback of protein redesign is that detailed structural knowledge of a protein is often unavailable, and, even when it is available, substitutions at sites buried inside proteins are more likely to break their structures and functions. Therefore, it is still very difficult to predict the effects of various mutations on the structural and functional properties of the mutated protein, although many studies have been done to predict the effects of AA substitutions on protein functions [168]. Another rational protein design method is computational protein design, which aims to design new protein molecules with a target folding protein structure, novel function and/or behavior. In this approach, proteins can be designed by transcendentally setting AA sequences compatible with existing or postulated template backbone structures (de novo design) or by making calculated variations to a known protein structure and its sequence (protein redesign) [169]. Rational protein design approaches make predicted AA sequences of protein that will fold into specific 3D structures. Subsequently, these predicted sequences should be validated experimentally through the chemical synthesis of an artificial gene, followed by protein expression and purification. The details of computational protein design methods will not be covered in this review; readers are referred to several recently published reviews [170, 171].

#### Directed evolution (protein engineering based on high-throughput library screening or selection)

The directed evolution approach (Fig. 17, the right panel) involves many technologies, such as gene library diversification, genotype–phenotype linkage technologies, display technologies, cell-free protein synthesis (CFPS) technologies, and phenotype detection and evaluation technologies [172]. This approach mimics the process of natural selection (Darwinian evolution) to evolve proteins toward a target goal. It involves subjecting a gene to iterative rounds of mutagenesis (creating a molecular library with sufficient diversity for the altered function), selection (expressing the variants and isolating members with the desired function), and amplification (generating a template for the next round). This process can be performed in vivo (in living cells), or in vitro (free in solutions or microdroplets). Molecular diversity is typically created by various random mutagenesis and/or in vitro gene recombination methods, as described in “Gene engineering”.

Functionally improved variants are identified by an HTS or selection method and then used as the parents for the next round of evolution. The success of directed evolution depends on the choices of both diversity-generation methods and HTS/selection methods. The key technology of HTS/selection methods is the linkage of the genotype (the nucleic acid that can be replicated) and the phenotype (the functional trait, such as binding or catalytic activity). Aptamer and ribozyme selection from nucleic acid libraries can be performed much faster than those of functional proteins because the nucleic acids themselves have binding or catalytic activities (i.e., selectable phenotypes), such that the genotype and phenotype are identical. However, since proteins cannot be amplified, it is necessary to have a linkage between the phenotype exhibited by the protein and the genotype (mRNA or DNA) encoding it to evolve proteins.

**Fig. 18:**
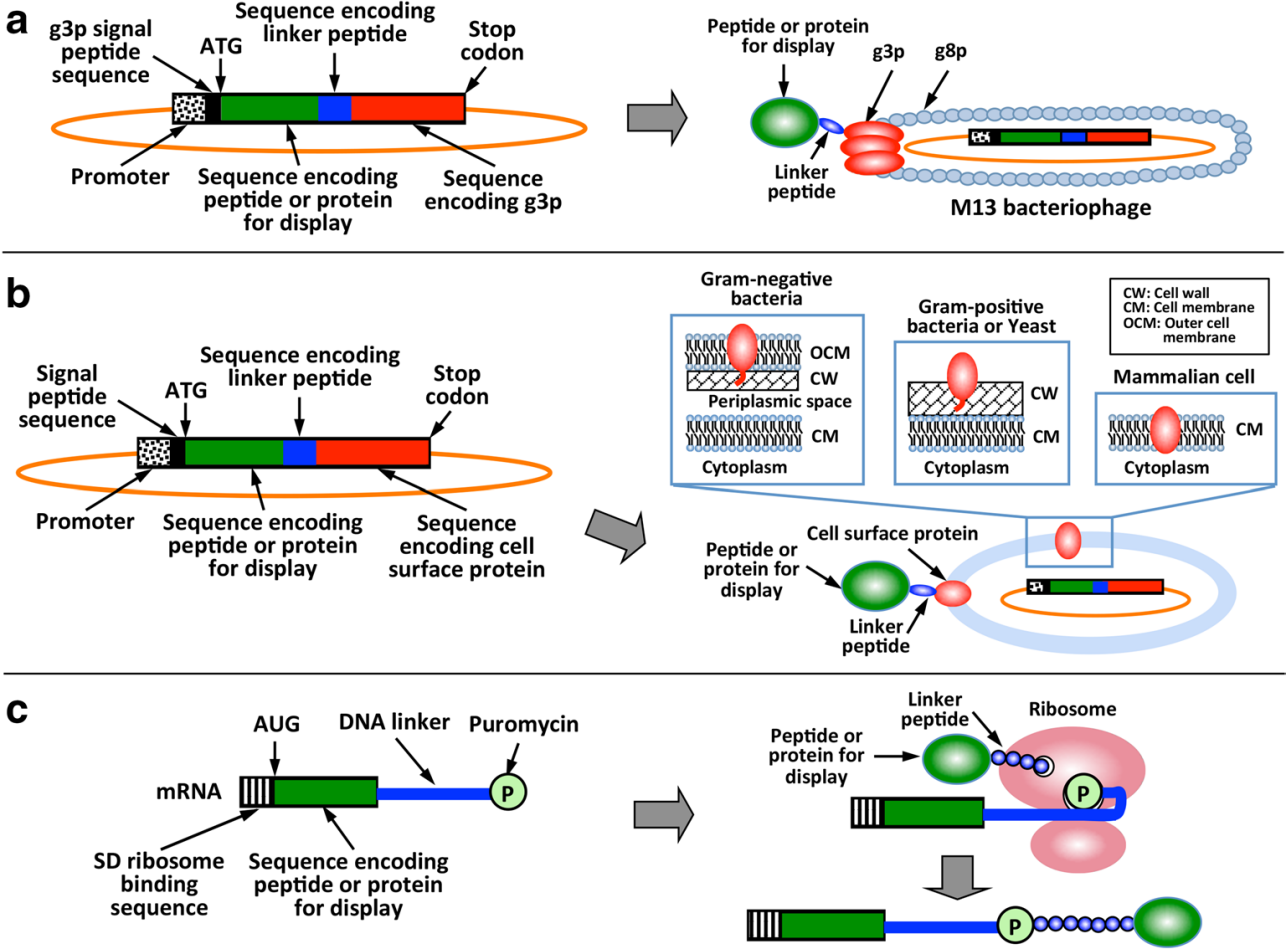
Various genotype–phenotype linkage technologies. **a** Phage display technology. **b** Cell surface display technologies: in vivo display on the surface of bacteria, yeast or mammalian cell. **c** RNA display technology

Many genotype–phenotype linkage technologies have been developed; these link proteins to their corresponding genes (Fig. 18) [172–174]. Genotype–phenotype linkage technologies can be divided into in vivo and in vitro display technologies. In vitro display technologies can be further classified into RNA display and DNA display technologies.

In vivo display technology includes phage display [175] and baculovirus display [176], in which a protein gene designated for evolution is fused to a coat protein gene and expressed as a fusion protein on the surface of phage and virus particles. Cell surface display technologies are also in vivo display technologies and use bacteria [177, 178], yeast [179, 180] and mammalian cells [181] as host cells, in which the fusion gene resulting from a protein gene and a partial (or full) endogenous cell surface protein gene is expressed and displayed on the cell surface. These in vivo display technologies can indirectly link a protein designated for evolution and its gene through the display of the protein on biological particles or cells. However, the library sizes of in vivo display technologies are usually restricted to the 10^8^–10^11^ size range by the efficiency of the transformation and transduction steps of their encoding plasmids.

In vitro display technologies are based on CFPS systems. Recent advances in CFPS technologies and applications have been reviewed elsewhere [182–185]. RNA display technology includes mRNA display and ribosome display [186]. mRNA display covalently links a protein to its coding mRNA through a puromycin linker that is covalently attached to the protein via ribosome-catalyzed peptide bond formation. Ribosome display noncovalently links a protein to its coding mRNA genetically fused to a spacer sequence lacking a stop codon through a ribosome because the nascent protein does not dissociate from the ribosome. Such display technologies using in vitro translation reactions can screen proteins that would be toxic to cells and can cover quite large libraries (10^15^) by bypassing the restricted library size bottleneck of in vivo display technologies (Table 1).

**Table 1:** Various display technologies

Technology (typical number of sequences screened per library)	Description	Strengths or *weaknesses*
Bacterial cell display (10^8^–10^9^)	Fusion gene libraries of the target proteins and bacterial surface proteins Fusion proteins are displayed on bacterial cell surface	Selects proteins displayed on bacterial cell surfaces Flow cytometry allows multiparameter, quantitative screening *Smaller library size* *Cannot screen proteins that would be toxic to cells*
Yeast or mammalian cell display (10^8^–10^10^)	Fusion gene libraries of the target protein and cell surface proteins of yeast or mammalian cells Fusion proteins are displayed on cell surface	Selects proteins displayed on eukaryotic cell surfaces Flow cytometry allows multiparameter, quantitative screening *Smaller library sizes* *Cannot screen proteins that would be toxic to cells*
Phage or baculovirus display (10^11^)	Fusion gene libraries of the target protein and phage or virus coat proteins Infected bacteria produces phage or virus particles displaying fusion protein libraries on the surface	Robust and quick *Cannot screen proteins that would be toxic to cells*
Ribosome display (10^15^)	mRNA-target protein complexes are displayed on stalled ribosomes in cell free protein synthesis system Reverse-transcription PCR allows amplification after rounds of selections	Large library size Can screen proteins that would be toxic to cells *Requires stringent conditions and stable proteins*
mRNA display (10^15^)	mRNA-target protein fusions are synthesized in cell free protein synthesis system by conjugating them through a puromycin linker Reverse-transcription PCR allows amplification after rounds of selections	Large library size Can screen proteins that would be toxic to cells *Works well with small proteins but not large ones* *Requires stringent conditions*

There are several in vitro DNA display technologies, such as CIS display [187], *M. Hae* III display [188], STABLE display [189], microbead display [190] and in vitro compartmentalization (IVC) [191]. CIS display noncovalently links RepA (DNA-binding protein) fusion protein and its coding DNA template through the interaction between RepA and the CIS element of the DNA template. For *M. Hae* III display, the DNA methyltransferase *M. Hae*III covalently links a protein and its DNA template. IVC technology uses the aqueous droplets in water–oil emulsions to compartmentalize individual genes and gene products. STABLE display and microbead display technologies utilize noncovalent biotin–streptavidin binding to link biotin-labeled DNA templates and streptavidin-fused proteins.

The details of HTS and selection methods, such as fluorescence-activated cell sorting-based phenotype detection and evaluation technologies coupled with these display technologies [172, 192–195], as well as the applications of the directed evolution of enzymes, antibodies, receptors and other proteins in such areas as environmental issues, catalysis, gene therapy, and therapeutic protein and vaccine development will not be covered in this review; readers are referred to several recently published articles and reviews [42, 172, 175, 177, 180, 181, 186, 187, 193–208].

Recent, significant advances in protein engineering have come through computational methods, such as SCHEMA, ProSAR, and ROSETTA. Computational design based on these methods greatly decreases the need for probing randomized sequence space, rendering the route to novel biocatalysts much more efficient [195, 209–212]. Therefore, in the future, more detailed knowledge about the relationship between protein structures and functions, as well as advancements in high-throughput technology, may greatly expand the capabilities of protein engineering.

### Chemical and enzymatic conjugation technologies

In the current postgenomic era, many studies require chemically modified proteins or protein bioconjugates that are impossible to prepare via standard ribosomal synthesis. Conjugation technologies to site-specifically modify proteins with diverse natural and unnatural functionalities have been developed in the last two decades. These technologies have been widely utilized to fabricate hybrid biomolecular material, such as protein/protein, protein/peptide, protein/nucleic acid, protein/lipid, protein/oligosaccharide, and protein/ligand hybrids, and hybrid materials comprising biomolecules and inorganic/organic materials for use in nanobio/bionanotechnology. These technologies range from classical chemical bioconjugation technologies targeting natural AAs to more sophisticated approaches, such as unnatural AA (UAA) incorporation based on amber stop codon suppression, bioorthogonal chemical conjugations, protein chemical ligations and enzymatic conjugations.

#### Chemical conjugation technologies targeting natural AAs

**Fig. 19:**
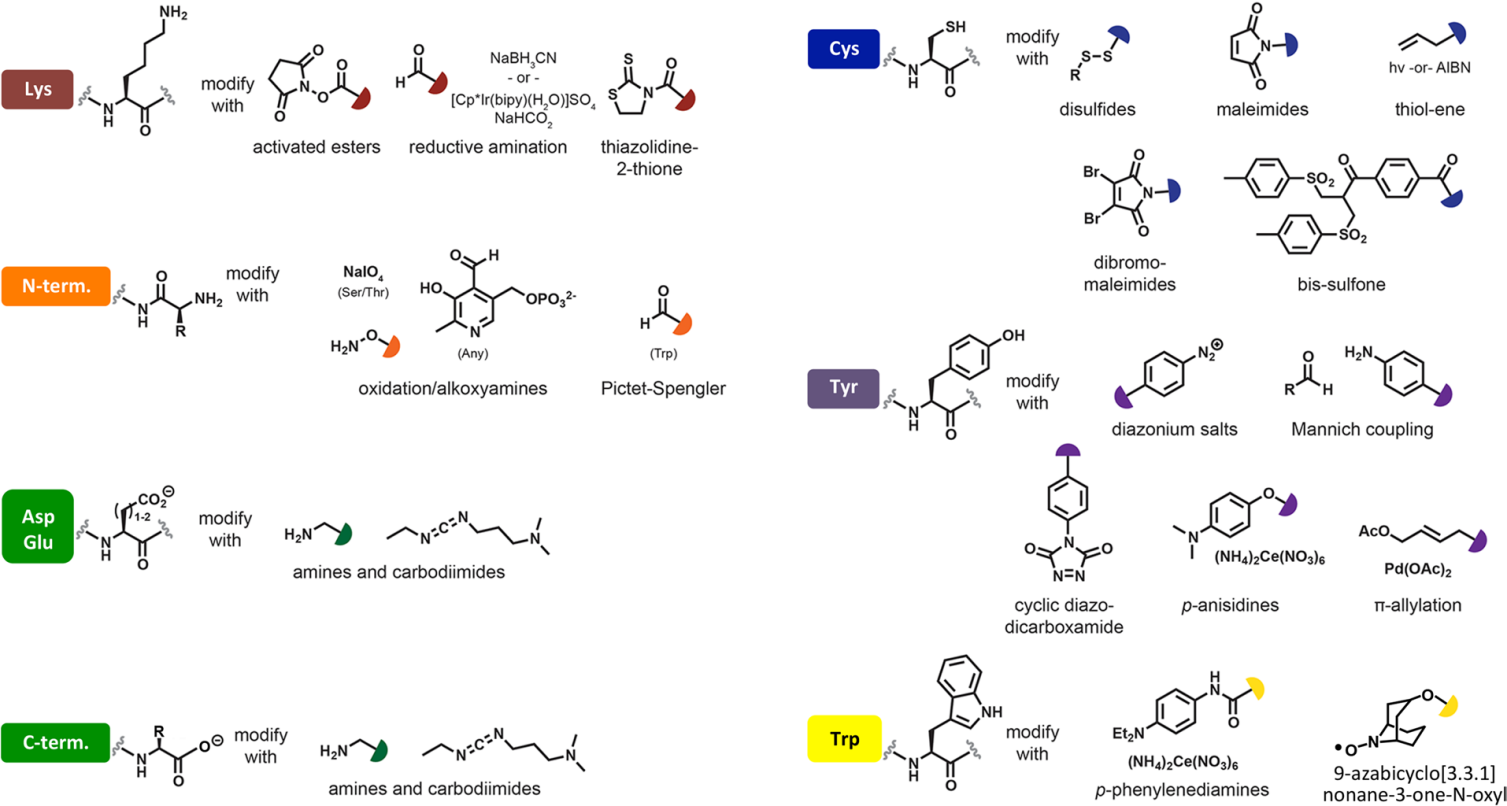
Standard chemical conjugation technologies for proteins targeting at side chains of natural AA (Figure adapted with permission from: Ref. [213]. Copyright (2015) American Chemical Society)

Standard chemical conjugation technologies for proteins target the side chains of natural AAs, such as the primary amine groups (R–NH_2_) of Lys residue and the N-terminus, the carboxylic acid groups (R–COOH) of Asp, Glu and the C-terminus, the thiol group (R–SH) of Cys, the phenyl ring of tyrosine (Tyr) and the indole ring of tryptophan (Trp) (Fig. 19) [213].

Lys is one of the most common AA residues in proteins with an average abundance of approximately 6% and is often surface-exposed due to its hydrophilicity; therefore, it is an excellent target site for conjugation. On the other hand, the N-terminus provides a more site-selective location but is not always surface-exposed. The primary amine of Lys has been predominantly functionalized with *N*-hydroxysuccinimidyl-esters (NHS-esters), NHS-ester sulfates or isothiocyanates. In these electrophilic reagents, NHS-esters are highly used for primary amine-targeted functionalization because of the reaction simplicity. A limitation of NHS-esters is a side reaction of hydrolysis in water (<5 h half-life), which accelerates as the pH increases above 7. This hydrolysis competes with desired reactions and reduces reaction efficiency [214].

The N-terminus can be selectively targeted for modification when it is sufficiently accessible and not post-translationally modified. The transamination reaction mediated by pyridoxal-5-phosphate can be applied to the modification of the N-terminal residue without the presence of toxic Cu(II) or denaturing organic cosolvents, although proteins possessing N-terminal serine (Ser), threonine (Thr), Cys, or Trp residues will be incompatible with this method because of known side reactions with aldehydes [215].

Asp and Glu are also the most common AA residues in naturally occurring proteins; they have an average abundance of approximately 12%, are often surface-exposed and are excellent target conjugation sites. The carboxylic acid side chains of Asp, Glu and the C-terminus can be functionalized by carbodiimide chemistry, typically using EDC, which has been widely used for covalently crosslinking a carboxylic acid and amine. However, the relatively high abundance of Lys, Asp and Glu and the high solvent accessibility of their side chains make it impossible to modify a single site on the protein surface using these methods.

Cys is not definitively hydrophilic or hydrophobic, and it is an attractive residue site for directed target conjugation because its average abundance in naturally occurring proteins is estimated to be approximately 1%. The relatively low abundance of Cys facilitates the genetic modification of the protein sequence to introduce a unique Cys. The nucleophilic side chain of Cys can be site-selectively targeted to create a well-defined conjugate. At slightly basic pH levels, the thiolate moiety can be modified with disulfides, maleimides, thiol-ene, dibromo-maleimides or bis-sulfone. Modification with disulfide (under mild oxidative condition) and maleimide (Michael addition) reagents produces disulfide and thiosuccinimide bond linkages that are not stable in the presence of free thiols, such as reduced glutathione (GSH) abundant in the cytoplasm of cells [213]. This GSH-sensitive conjugation property has been positively utilized for the release of drug delivery system payloads in the cytoplasm. In contrast, the ring-opening hydrolysis of thiosuccinimide using maleimide derivative incorporating a basic amino group adjacent to the maleimide, positioned to provide intramolecular catalysis of thiosuccinimide ring hydrolysis, yields a stable conjugate (e.g., an antibody–drug conjugate) [216].

Methods for the conjugation of Tyr, which has an average abundance of 3% in proteins, have also been developed. In the presence of strong oxidizing agents (e.g., H_2_O_2_) and appropriate catalysts, the phenolic side chain of the Tyr residue can crosslink with other phenolic compounds. The oxidizing agents required to catalyze these reactions are not discerning, and there is concern over causing undesired side reactions to other portions of proteins. To overcome this problem, a Tyr coupling reaction has been developed; it involves an electrophilic reagent, imines formed in situ from aldehydes and electron-rich anilines. This three-component Mannich-type coupling reaction is highly selective for Tyr and proceeds under mild conditions [217].

Conventional methods for the conjugation of Trp, which has an average abundance of approximately 1%, require toxic heavy metals or biochemically incompatible conditions. Some of these methods also exhibit cross reactivity with other AAs (particularly Tyr), thus limiting the range of applications. Recently, a transition metal-free method using 9-azabicyclo[3.3.1]nonane-3-one-*N*-oxyl (keto-ABNO) for the conjugation of Trp was reported. This new method showed novel features, such as high Trp selectivity, the formation of single conjugates with high homogeneity, facile conjugation at an ambient temperature and nearly neutral pH and a short reaction time [218].

#### Chemical conjugation technologies targeting UAAs

Although the site-specific substitution of AAs with rare AAs, such as Cys or Tyr, provides multiple options for the covalent functionalization of proteins, it is possible to damage the folding or function of the proteins when the genetic modifications for such chemical conjugations become too extensive. The incorporation of UAAs into proteins allows for greater flexibility in protein modifications. Unlike the natural AA residues, these UAAs can contain entirely unique reactive moieties, such as azide, cyano, iodo, bromo, boc, and dansyl groups, and thereby afford completely bioorthogonal reactivity that can occur inside of living systems without interfering with native biochemical processes. The site-specific incorporation (SSI) of UAAs utilizes nonsense codons to incorporate one or a few UAAs into a single or multiple defined locations in a protein. The amber stop codon suppression is commonly employed, in which the target-location codon is mutated to an amber stop codon [219]. To incorporate the UAA, a tRNA and aminoacyl tRNA-synthetase (aaRS) are engineered to pair with the UAA and recognize the amber codon as a sense codon. SSI substitutes a natural AA with an unnatural analog, where the endogenous aminoacyl tRNA-synthetase (aaRS) accepts the UAA and aminoacylates the appropriate tRNA with the UAA. Incubating an auxotrophic expression host in culture media containing the analog UAA instead of the specific natural AA leads to UAA incorporation at nearly every location where the specific natural AA would have been incorporated [220]. The SSI of UAAs has been further extended to CFPS systems [219, 221]. The incorporation of multiple different UAAs has been achieved by the extension of codon-anticodon pairs using a different four-base codon for each tRNA [222]. Technology using acylating ribozyme (flexizyme) instead of ssRS has been developed for in vitro semi-enzymatic synthesis and acylation [223]. Therefore, SSI is minimally invasive and allows the incorporation of any UAA into a specific site of a protein with minor effects.

#### Bioorthogonal chemical conjugation technologies

**Fig. 20:**
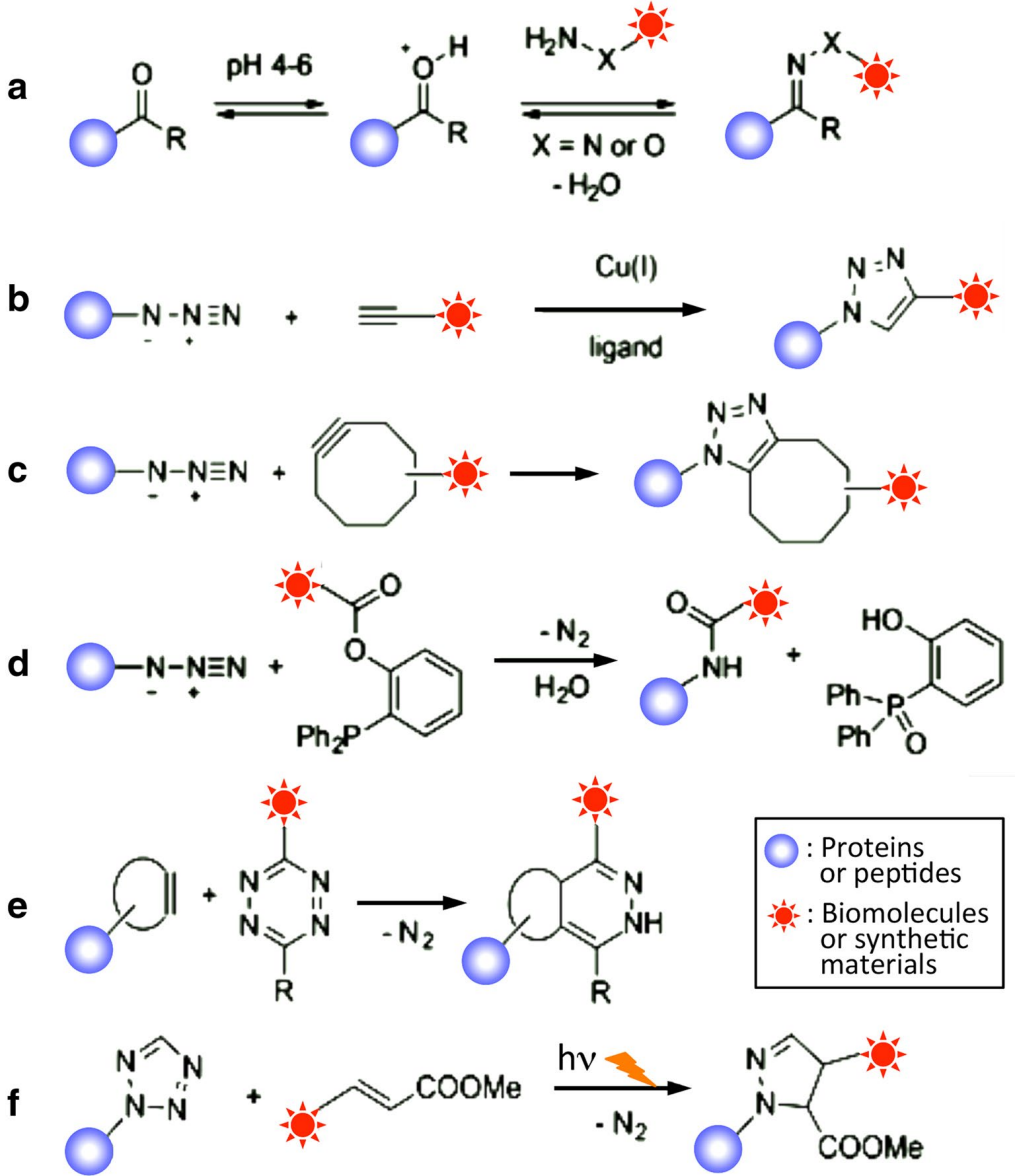
Chemoselective bioconjugation reactions. **a** Ketone/hydroxylamine condensations. **b** Copper-catalyzed alkyne–azide Huisgen cycloadditions. **c** Strain-promoted alkyne-azide cycloadditions. **d** Staudinger ligation. **e** Diels–Alder cycloadditions. **f** Photo-click cycloadditions (Figure adapted with permission from: Ref. [224]. Copyright (2014) American Chemical Society)

UAA-incorporated proteins or peptides can be chemoselectively conjugated with other biomolecules and synthetic inorganic/organic materials bearing bioorthogonal functional groups. Other biomolecules, such as nucleic acids, lipids, and synthetic inorganic/organic materials modified with bioorthogonal functional groups, can also be subjected chemoselectively to a bioconjugation reaction. This reaction should occur in an aqueous solution at near physiological pH, have rapid kinetics even with submillimolar concentrations of reactants, and occur at physiological temperatures. The chemical reactions that satisfy these requirements include the keton/hydroxyamine condensations, Huisgen [3 + 2] cycloaddition, the Staudinger ligation, the Diels–Alder cycloadditions and a photo-Click cycloadditions (Fig. 20) [224, 225].

##### Conjugation reactions through aldehydes and ketones

The carbonyl groups of aldehydes and ketones can specifically react with strong α-effect nucleophiles, such as hydrazides (R–N–NH_2_) and alkoxyamines (R–O–NH_2_), under acidic conditions (pH 4–6) to produce stable hydrazones and oximes, respectively. Since these ketone/aldehyde condensations show rather slow kinetics with second-order rate constants, large excesses of the conjugation reagent are necessary in order to achieve good labeling efficiency. In general, ketone/aldehyde condensations are best suited for conjugation reactions in vitro or at the cell surface because the reaction requires an acidic pH and high concentrations of the labeling reagent, which are problematic in terms of cell toxicity [224].

##### Conjugation reactions through azides

The azide group is truly orthogonal in its reactivity to the majority of biological functionalities. The Huisgen 1,3-dipolar cycloaddition of alkynes and azides has been widely adopted since this reaction is both selective and high yielding when catalyzed by Cu(I) for Cu-chelating propylene derivatives or by strain release for strained cycloheptyne derivatives. Another cycloaddition reaction, the inverse-electron-demand Diels–Alder reaction between tetrazines and strained alkenes or alkynes, yields dihydropyridazines or pyridazines with nitrogen gas as the only byproduct. These reactions have recently been explored as chemoselective reactions for labeling and manipulating biomolecules in their native states. The reactions are extraordinarily fast (up to 10^5^ M^−1^ s^−1^) and have improved second-order kinetics relative to the chelating Cu(I)-catalyzed azide-alkyne cycloaddition (10–200 M^−1^ s^−1^) [224]. The 1,2,3-triazole linkage formed in the cycloaddition reaction (click reaction) is thermodynamically and hydrolytically stable. This reaction is insensitive to aqueous conditions and pH levels ranging from 4 to 12, succeeds over a broad temperature range, and tolerates a wide variety of functional groups. Pure products can be easily isolated by simple filtration or extraction without chromatography or recrystallization. Many other bioorthogonal conjugation reactions have been reported; readers can refer to recent reviews [224, 225].

#### Chemical ligation technologies

**Fig. 21:**
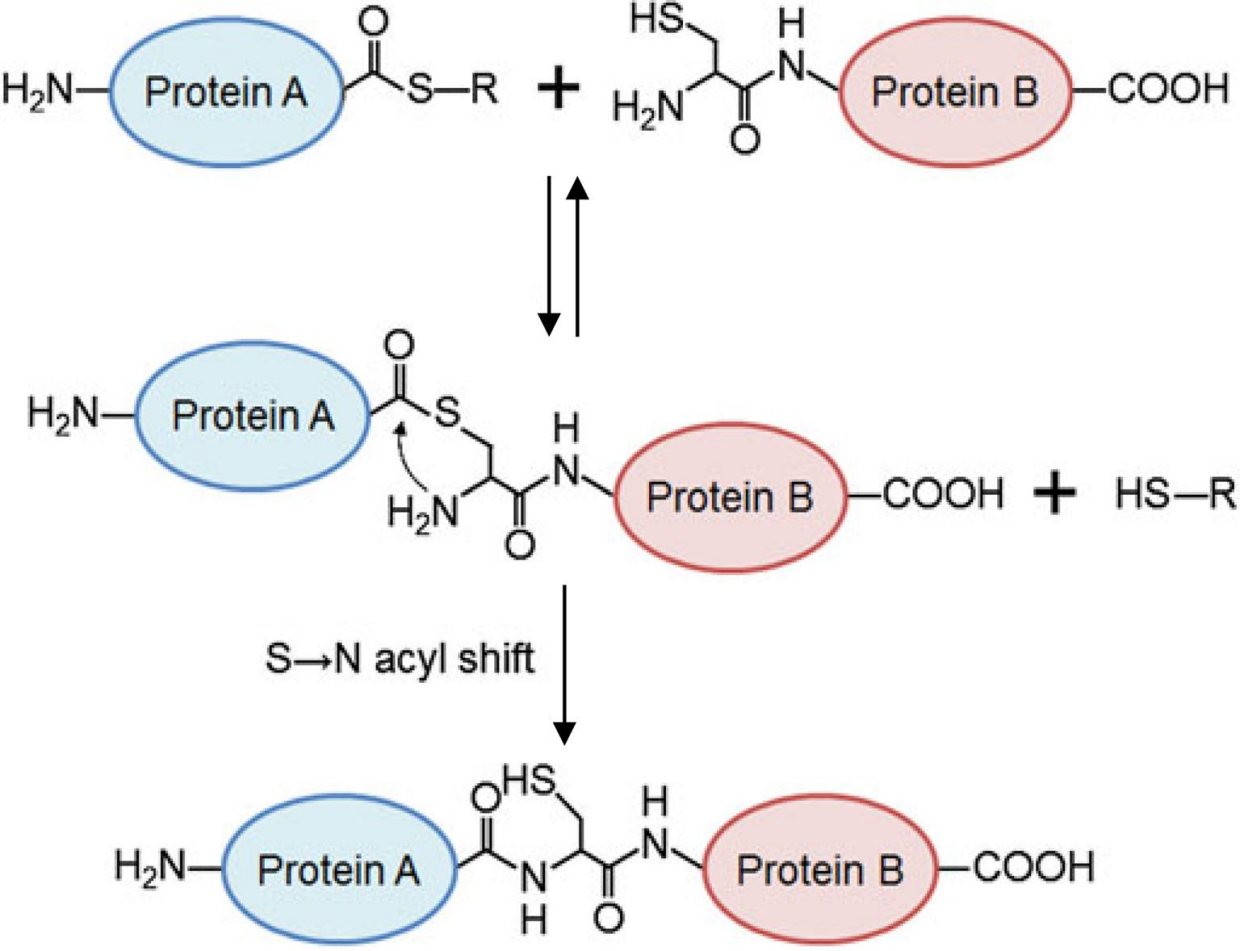
Native chemical ligation. Native chemical ligation (NCL) is a chemoselective coupling reaction that links a peptide fragment containing an N-terminal Cys (α-Cys) residue and another peptide fragment bearing a C-terminal α-thioester group by a native peptide bond (Figure reproduced with permission from: Ref. [106]. Copyright (2012) Springer)

Native chemical ligation (NCL) has become the most general and robust method for the conjugation of protein–peptide, protein–protein, protein–DNA, and protein–NP materials because it is simple, general, and has a high yield efficiency [226]. NCL is a chemoselective coupling reaction that generates a native peptide bond by a reversible transthioesterification between a peptide fragment containing an N-terminal Cys residue (α-Cys) and another peptide fragment bearing a C-terminal α-thioester group, followed by an irreversible intramolecular N-S acyl shift (Fig. 21). This reaction proceeds efficiently under physiological conditions and is compatible with all natural AA side chains. Therefore, through the recombinant preparation of proteins having an α-Cys residue, NCL can be used to generate proteins containing modifications at their N-termini. It is advantageous to conduct NCL in an aqueous solution at a neutral pH even though a C-terminal α-thioester derivative can be competitively hydrolyzed. Recent extensions of NCL, such as ligation rate acceleration, chemoselective post-ligation modifications, and the streamlined ligation of multiple peptide fragments, have been reviewed [227].

**Fig. 22:**
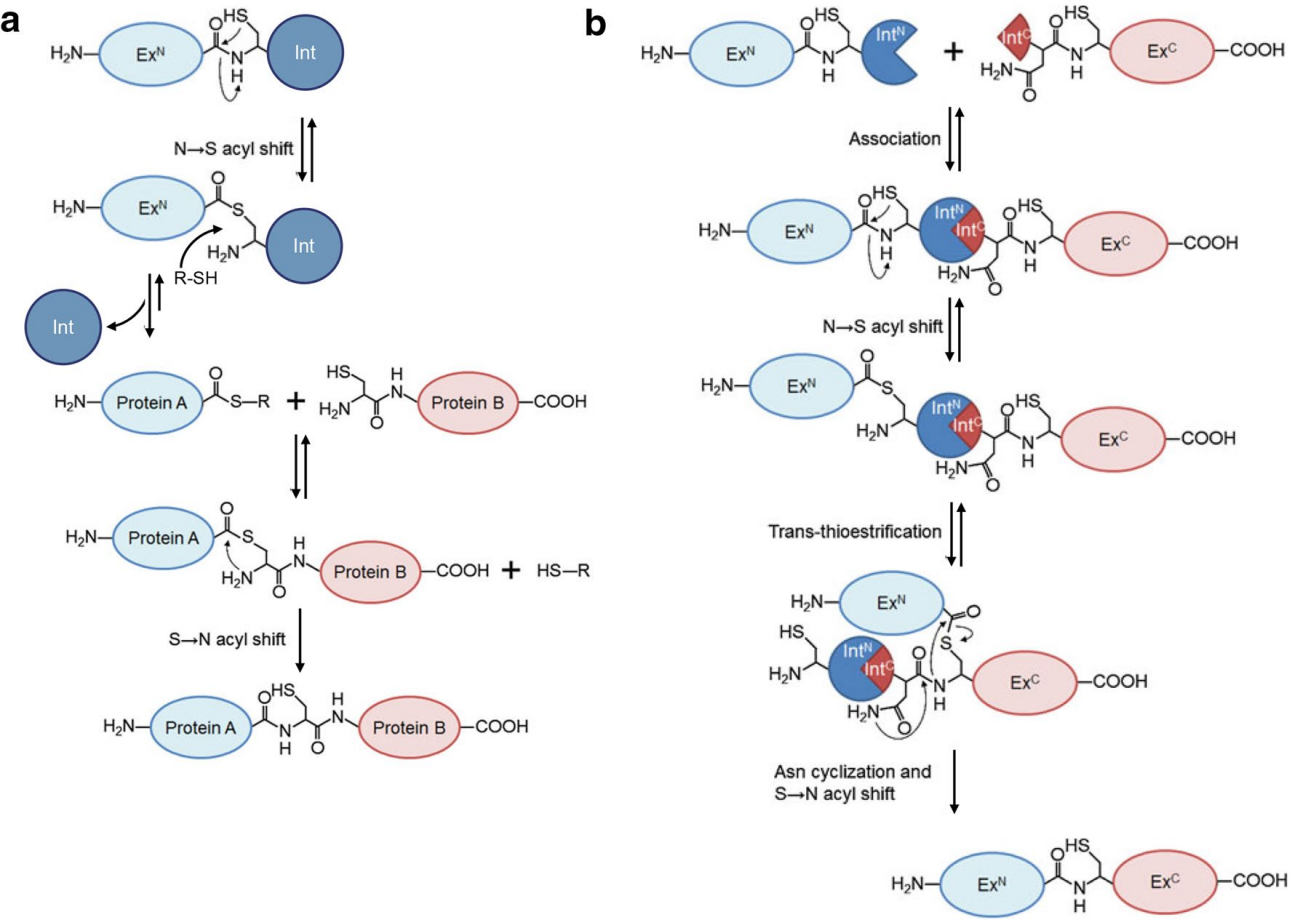
Intein-based chemical conjugation. **a** Expressed protein ligation (EPL) is a semisynthetic version of NCL in which synthetic and recombinant polypeptides are chemically ligated together. Proteins (A) expressed as intein fusions can be cleaved from the intein with a variety of thiols to give the corresponding α-thioester derivative. Proteins (B) containing N-terminal Cys can be made recombinantly by masking the Cys with a protease tag that can be later removed. **b** Protein trans-splicing (PTS) post-translationally links two protein fragments. An intein domain is split into two fragments, Int^N^ and Int^C^, which are fused to the flanking exteins, Ex^N^ and Ex^C^. Ex^N^–Int^N^ and Int^C^–Ex^C^ associate and fold to form a functional intein. This functional intein can restore protein splicing activity to excise itself, and to conjugate ExN and ExC with a peptide bond (Figures adapted with permission from: Ref. [106]. Copyright (2012) Springer)

Expressed protein ligation (EPL) and protein trans-splicing (PTS) are both intein-based chemical conjugation technologies that permit the assembly of a protein from smaller synthetic and/or recombinant unprotected polypeptide building blocks (Fig. 22). An intein is an internal protein domain that can autocatalytically excise itself from a precursor protein. The cis-splicing of intein by the addition of high concentrations of thiol derivatives can be used to generate a C-terminal α-thioester of a protein from protein-intein fusion. In EPL, one or more of the peptides is of recombinant origin, but the actual ligation step is still a chemical process and can be performed under a wide range of reactions to introduce a variety of functional materials, such as fluorophores, UAAs, isotopic labels, and post-translational modifications, into a large number of proteins [228]. By contrast, PTS post-translationally links two recombinant protein fragments. An intein domain is split into two fragments (split intein or trans-splicing intein), Int^N^ and Int^C^, which are fused to the flanking polypeptides, termed the N and C exteins (Ex^N^ and Ex^C^). The ligation step in PTS must be performed under conditions compatible with protein folding because the process involves the functional reconstitution of a split intein. In this step, Ex^N^–Int^N^ and Int^C^–Ex^C^ associate, fold to form a functional intein, restore autocatalytic protein splicing activity to excise the Int^N^–Int^C^, and ligate the flanking Ex^N^ and Ex^C^ with a peptide bond of Cys.

Although the advances in NCL, EPL and PTS made it possible to precisely introduce a variety of functional materials into peptides and proteins, these technologies also have some drawbacks, as follows. (1) The preparation of synthetic peptide α-thioesters is still technically difficult. (2) Since the ligation process is a chemical reaction, the higher concentrations of both or either of the reactants are required. (3) The application of EPL to many disulfide bond-containing proteins is restricted or complicated since the use of high concentrations (usually more than several tens of mM) of thiol derivatives is needed to induce thiolysis of the protein-intein fusions. (4) The expression of intein-based fusion proteins often results in the formation of inclusion bodies due to the large protein sizes and poor solubility, which requires additional refolding steps.

#### Enzymatic conjugation technologies

**Fig. 23:**
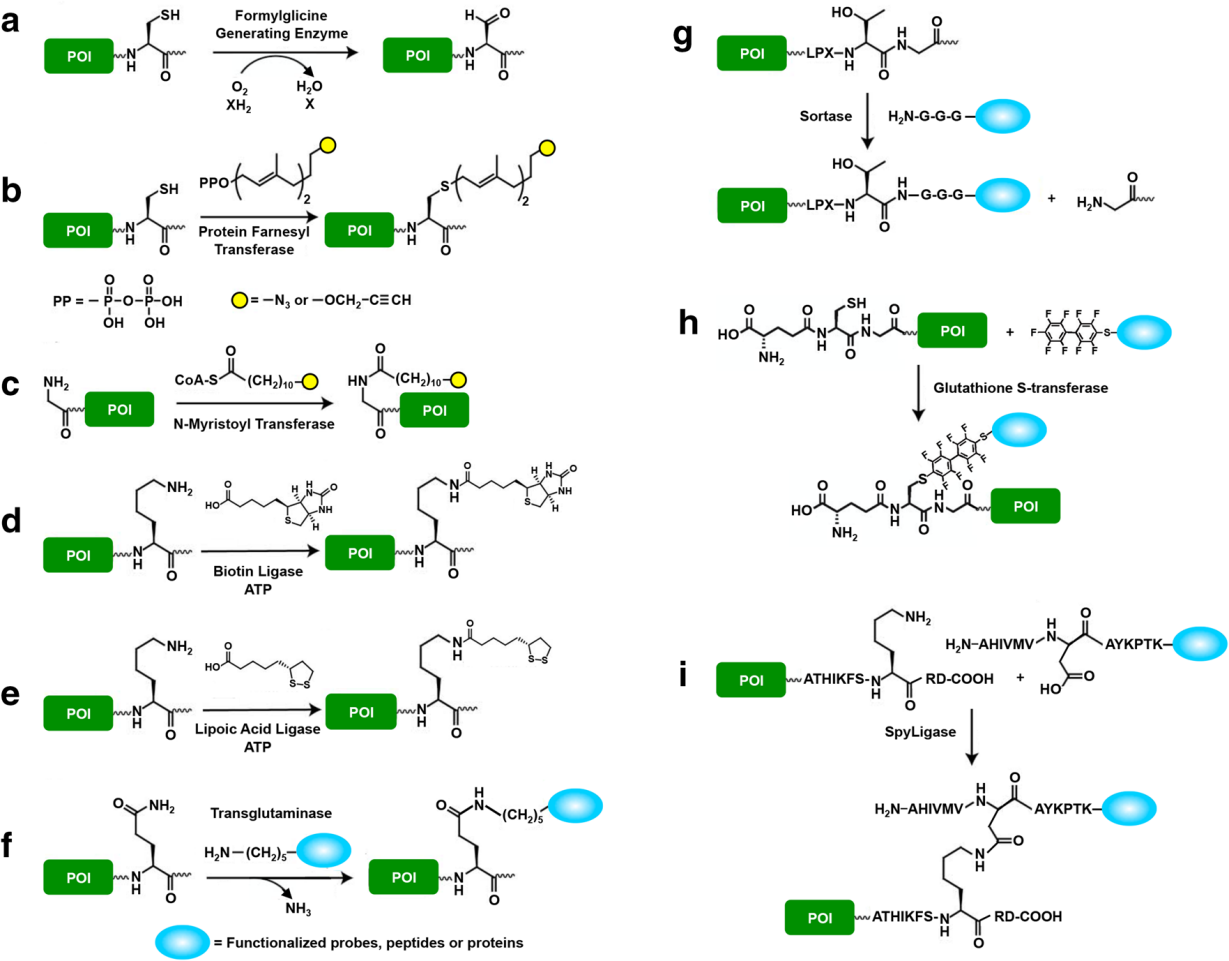
Chemoenzymatic labeling strategies of the protein of interest (POI) using post-translational modification enzymes. **a** Formylglycine generating enzyme (FGE) recognizes LCXPXR peptide motif and converts the side chain of Cys residue into an aldehyde group. The POI fused to the aldehyde tag can be further functionalized with aminooxy or hydrazide probes. **b** Farnesyltransferase (FTase) recognizes the four AAs sequence CA_1_A_2_X (A_1_ and A_2_ are non-charged aliphatic AAs and X is C-terminal Met, Ser or Phe) at the C-terminus and catalyzes the attachment of the farnesyl isoprenoid group to the Cys residue. The POI can be further labeled by bioorthogonal chemical conjugation of the farnesyl moiety functionalized with azide or alkyne. **c** *N*-Myristoyl transferase (NMT) recognizes the GXXXS peptide motif at the N-terminus and attaches a myristate group to an N-terminal Gly residue. The POI can be further labeled by bioorthogonal chemical conjugation of myristate moiety functionalized with azide or alkyne. **d** Biotin ligase recognizes the GGLNDIFEAQKIEWH peptide motif derived from biotin carboxyl carrier protein and catalyzes the transfer of biotin from an ATP intermediate (biotinyl 5′-adenylate) to Lys residue. Biotinylated POI can then be labeled with streptavidin conjugated with a variety of chemical probes. **e** Lipoic acid ligase recognizes the GFEIDKVWYDLDA peptide motif and catalyzes the attachment of lipoic acid or its derivatives to Lys residue in the motif. **f** Transglutaminase (TGase) catalyzes the transamination reaction and forms an iso-peptide bond between Gln in POI and Lys residue-functionalized small molecule probes, peptides or proteins. **g** Sortase cleaves LPXTG peptide tag fused to POI between Thr and Gly residue and conjugates oligo Gly-functionalized small molecule probes, peptides or proteins to POI by forming a peptide bond between Thr and Gly residues. **h** GST catalyzes Cys arylation and conjugates probes bearing a 4-mercaptoperfluorobiphenyl moiety to the N-terminal γ-Glu-Cys-Gly sequence of POI. **i** SpyLigase catalyzes the generation of an isopeptide bond between Lys residue in KTag and Asp residue in SpyTag

In nature, numerous proteins are post-translationally modified by enzymes and play important roles in controlling cellar processes, such as metabolism, signal transduction, gene expression, and cell differentiation. These enzymes participating in post-translational modifications catalyze the covalent addition of some chemical groups (e.g., phosphate, acetate, amide, and methyl groups and biotin, flavins, carbohydrates and lipids) to the N- or C-terminus or a side chain of an AA residue at specific site in a protein; these enzymes can also catalyze the cleavage and ligation of peptide backbones in proteins. Natural post-translational modifications of proteins are generally efficient under physiological conditions and site-specific. Therefore, a variety of transferase or ligase enzymes have been repurposed for site-specific protein modification. Typically, a small tag peptide sequence incorporated into the target protein is recognized by the post-translational modification enzyme as a substrate and then transfers functional moieties from an analog of its natural substrate onto the tag (Fig. 23). Examples include formylglycine-generating enzyme (FGE), protein farnesyltransferase (PFTase), *N*-myristoyltransferase (NMTase), biotin ligase (BirA), lipoic acid ligase (LAL), microbial transglutaminase (MTGase), sortase A (SrtA), glutathione *S*-transferase (GST), SpyLigase, and several engineered self-labeling protein tags. Except for self-labeling protein tags, a primary benefit of this approach is the small size of the peptide tag that must be incorporated into proteins, which ranges from 3 to 15 residues. Some enzymes only recognize the tag peptide at a specific position in the primary sequence of the protein (often the N- or C-terminus), while others are not inherently limited by tag position.

Enzymatic protein conjugation technologies, including non-site-specific crosslinking by such oxidoreductases as peroxidase, laccase, tyrosinase, lysyl oxidase, and amine oxidase, are reviewed elsewhere [105]. Here, we briefly review recent enzymatic conjugation technologies for site-specific protein conjugation and crosslinking of biomolecules and synthetic materials. The applications of enzymatic conjugations and modifications of proteins with other biomolecules and synthetic materials are limited to recombinant proteins harboring additional protein/peptide tags. However, protein functionalization using enzymatic conjugations is a promising method because it is achieved simply by mixing proteins without special techniques. The details of enzymatic conjugation technology applications will not be covered in this review; readers are referred to several recently published reviews [229–232].

##### FGE

The FGE oxidizes Cys or Ser residue to formylglycine (FGly) within a conserved 13-AA consensus sequence found in prokaryotic Type I sulfatases. The modification is thought to occur co-translationally, before protein folding. The consensus sequence can be incorporated into heterologous proteins expressed in *E. coli*, where it is modified efficiently by a co-expressed bacterial FGE. Furthermore, the minimized core motif sequence CX(P/A)XR or SXPXR, derived from the most highly conserved portion of the FGE recognition site, directed the efficient conversion of Cys or Ser to FGly. The aldehyde-bearing residue FGly can be subsequently used for covalent conjugation using complementary aminooxy- or hydrazide-functionalized moieties by ketone-reactive chemistries (Fig. 23a) [233].

##### PFTase

PFTase is an α/β heterodimer enzyme that catalyzes the transfer of a farnesyl isoprenoid group from farnesyl pyrophosphate (FPP) through a thioether bond to a sulfur atom on a Cys in a tetrapeptide sequence (denoted as a CA_1_A_2_X-box, here C is Cys, A_1_ and A_2_ are aliphatic AAs, and X is one of a variety of AAs) four residues from the C-terminus (Fig. 23b). Since PFTase can tolerate many simple modifications to the aldehyde-containing isoprenoid substrate, it can be used to introduce a diverse range of functionalities into proteins containing a CA_1_A_2_X-box positioned at the C-terminus. Subsequent chemoselective reactions with the resulting protein can then be used for a wide range of applications. The catalytic activity of PFTase toward various FPP analogs has been greatly improved by site-directed mutagenesis around the substrate-binding pocket of PFTase [234].

##### NMTase

NMTase from *Candida albicans* catalyzes the acyl transfer of myristic acid from myristoyl-CoA to the amino group of an N-terminal glycine (Gly) residue of a protein to form an amide bond. NMTase recognizes the sequence GXXX(S/T), where X can be any AA (Fig. 23c). This enzyme can successfully transfer alkyne- and azide-containing myristic acid analogs that incorporated the bioorthogonal groups at the distal end of the lipid to the N-terminal Gly residue of recombinant proteins containing an N-terminal myristoylation motif. This method provides a convenient and potentially general method for N-terminal-specific recombinant protein labeling [235].

##### BirA

BirA from *E. coli* catalyzes the adenosine triphosphate (ATP)-dependent amide bond formation between the carboxylic group of biotin and the ε-amino group of a Lys in an acceptor peptide sequence (23 AA residues) (Fig. 23d). This acceptor sequence was further optimized to a 15-AA acceptor peptide sequence (GLNDIFEAQKIEWHE) [236]. BirA can be used to site-specifically conjugate a biotin moiety to recombinant proteins by the genetic fusion of the BirA recognition acceptor peptide sequence with the target protein. The enzymatic biotin labeling to a protein allows the subsequent formation of very strong noncovalent conjugate with avidin due to the low dissociation constant between biotin and avidin (∼10^−15^ M). Another orthogonal acceptor sequence for yeast BirA has been further developed to enable two-color imaging [237]. The substrate tolerance of BirA was also expanded to biotin analogs, including ketone, azide, and alkyne groups, which contain alternative functionalities suitable for bioorthogonal reactions [238].

##### LAL

LAL from *E. coli* catalyzes the ATP-dependent amide bond formation between the carboxylic group of lipoic acid and the ε-amino group of a lysine in an optimized 13-AA recognition acceptor sequence (GFEIDKVWYDLDA) (Fig. 23e). The Trp 37 residue at the lipoic acid-binding pocket of LAL was substituted with small AA residues to accept a wider range of lipoic acid analogs containing an aliphatic azide, aryl-aldehyde, or aryl-hydrazine moiety [239]. These lipoic acid analogs are attached to a Lys residue in the acceptor sequence of a protein and are then used to conjugate diverse functional molecules by bioorthogonal reactions.

##### MTGase

Transglutaminase is a unique enzyme that catalyzes the acyl-transfer reaction between the γ-carboxyamide group of a Gln residue in proteins and a wide variety of unbranched primary amines, commonly the ε-amino group of a Lys residue, and forms an isopeptide bond between the side chain of Gln residues and primary amines (Fig. 23f). Because this conjugation reaction is irreversible, involves the release of ammonia and proceeds quickly even under low temperature conditions (~15 °C), the conjugation product is stable, and a high yield can be obtained. MTGase is isolated from *Streptomyces mobaraensis*, which is widely used in the food industry, and recognizes various peptide sequences consisting of Gln residues. A notable correlation was observed between the polypeptide chain regions of high temperature factor (B-factor) determined crystallographically and the MTGase attacking sites, thus indicating the role of polypeptide chain mobility or local unfolding in dictating site-specific enzymatic modifications [240]. Consequently, enhanced MTGase polypeptide chain flexibility limits the enzymatic reaction with Gln residues on rigid polypeptide in globular proteins. Therefore, it is possible to predict the site(s) of Gln residue modifications by MTGase on the basis of local structure and dynamics of polypeptide chain containing Gln residue.

Because of its openness with regard to the primary amine substrate, MTGase is an attractive catalyst for generating protein conjugates with small functional molecules, lipids, nucleic acids, synthetic polymers, e.g., PEG, peptides and even other proteins. Although the substrate specificity of MTGase toward the polypeptide sequence containing a Gln residue (Q-tag) has not yet been clarified, the Q-tag derived from the polypeptides of globular proteins, the ribonuclease S-peptide (KETAAAKFERQHMDS and its Lys to Ala-substituted peptide AETAAAAFERQHMDS), the F-helix peptide of horse heart myoglobin (PLAQSH) or the designed N-terminal oligo-Gly tag (N-Gly_5_), which are recognized as a Gln-substrate by MTGase, can be utilized as Q-tag substrates [108, 241–244]. For protein modification by MTGase, these Q-tags are incorporated at the N- or C-terminus or inside the loop region of proteins by genetic means. Subsequently, MTGase can site-specifically conjugate the Q-tag in the protein with a primary amine-containing short synthetic linker or a Lys residue-containing polypeptide tag (KTag) harboring a functional moiety. However, one of the drawbacks of conjugating proteins possessing many Lys and Gln residues is that the activity of MTGase toward Gln and Lys residues makes it difficult to control the site(s) of modification.

##### SrtA

SrtAs are cell envelope-bound housekeeping transpeptidases from gram-positive bacteria. SrtA attaches surface proteins, such as virulence factors, to the penta-Gly motif of branched lipid II, the peptidoglycan precursor. SrtA recognizes the peptide sequence (LPXTG) and catalyzes the cleavage of the amide bond between the Thr and Gly residues by means of an active site Cys residue (Cys184) (Fig. 23g). This process generates a covalent acyl-enzyme intermediate. The carboxyl group of the Thr of the thioester intermediate then undergoes nucleophilic attack by an amino group of the oligo-Gly substrates, producing ligated products and altering the primary structure. Recent reports have demonstrated that the ε-amino group of Lys residues can also act as a nucleophile instead of the ε-amino group of oligo-Gly [245]. Since both of the optimized recognition peptide sequences, LPETGG [246] and oligo-Gly with more than two repeats [247], for SrtA-mediated transpeptidation are very short, these motifs can be easily incorporated into proteins or polypeptides either by standard genetic means or chemical peptide synthesis. Benefiting from its simplicity and specificity, a soluble truncated *Staphylococcus aureus* SrtA that lacks the N-terminal membrane-anchoring motif has begun to be applied for a wide variety of protein engineering and bioconjugation purposes, including the in situ site-specific fluorescent labeling of membrane proteins [248–252] and the fabrication of an electrochemically active protein bilayer on electrodes [253].

Unfortunately, since this conjugation reaction is reversible and the acyl-enzyme intermediate is hydrolyzed by water even in the presence of enough oligo-Gly nucleophiles, the conjugation reaction does not proceed to completion. However, we have overcome this limitation by introducing a β-hairpin structure around the ligation site of products and preventing substrate recognition by SrtA, thereby successfully stabilizing conjugation products and providing a high yield [254]. *S. aureus* SrtA needs Ca^2+^ for stabilizing the active site conformation, and its strong Ca^2+^ dependency makes *S. aureus* SrtA difficult for use under low Ca^2+^ concentrations and in the presence of Ca^2+^-binding substances. To overcome this problem, we designed an *S. aureus* SrtA heptamutant (P94R/E105K/E108A/D16N/D165A/K190E/K196T) that exhibited a high Ca^2+^-independent catalytic activity and successfully catalyzed a selective protein–protein ligation in living cells, which usually retain low Ca^2+^ concentrations [255]. These recent advances in *S. aureus* SrtA-mediated ligation will contribute to the development and design of many other protein conjugates and multienzyme complexes both in vitro and in vivo.

##### GST

GST catalyzes conjugation reactions between the Cys residue of glutathione (GSH, γ-Glu-Cys-Gly) and various electrophiles and allows the cell to detoxify xenobiotics in vivo (Fig. 23h). The ubiquitous nature of GST facilitates this bioconjugation with polypeptides bearing an N-terminal GSH in aqueous media and enables the chemo- and regioselective functionalization of a single Cys thiol group of GSH based on a nucleophilic aromatic substitution reaction between Cys residues and perfluoroarenes, even in the presence of other unprotected Cys residues and reactive functional groups on the same polypeptide chain. This conjugation reaction can be carried out over a wide range of temperatures (4–60 °C) and in co-solvent system with the addition of organic solvents (up to 20%) [256]. However, this technology is currently limited to peptide-based couplings due to the requirement for both an N-terminal γ-Glu-Cys-Gly sequence and a perfluoraryl reaction partner.

##### SpyLigase

SpyLigase is an artificial ligase obtained by engineering a domain (CnaB2) from the fibronectin adhesion protein FbaB of *Streptococcus pyogenes* (Spy), which is essential for the bacteria to invade human cells. Within CnaB2, there is a post-translational modification to form an isopeptide bond between Lys31 and Asp117 residues, which is catalyzed by an apposed Glu77 residue. Based on the 3D structure and isopeptide bond formation mechanism of CnaB2, the domain was rationally split into three parts, SpyTag (AHIVMVDAYKPTK), KTag (ATHIKFSKRD) and SpyLigase (11 kDa, containing the catalytic Glu77 residue). SpyLigase was derived from CnaB2 first by the removal of SpyTag and KTag, and then by circular permutation via replacing residues from the C-terminus of CnaB2 with a Gly/Ser linker, followed by N-terminal CnaB2 residues. SpyLigase not only can ligate KTag and SpyTag fused at the C- or N-terminus of peptides but can also direct the ligation of KTag to SpyTag inserted in the middle of a protein (Fig. 23i). The yield of conjugation products decreased from approximately 50–10% by elevating the reaction temperature from 4 to 37 °C, likely due to a dynamic change in the secondary structure of SpyLigase [257].

#### Self-labeling protein tag-based chemoenzymatic conjugation technologies

**Fig. 24:**
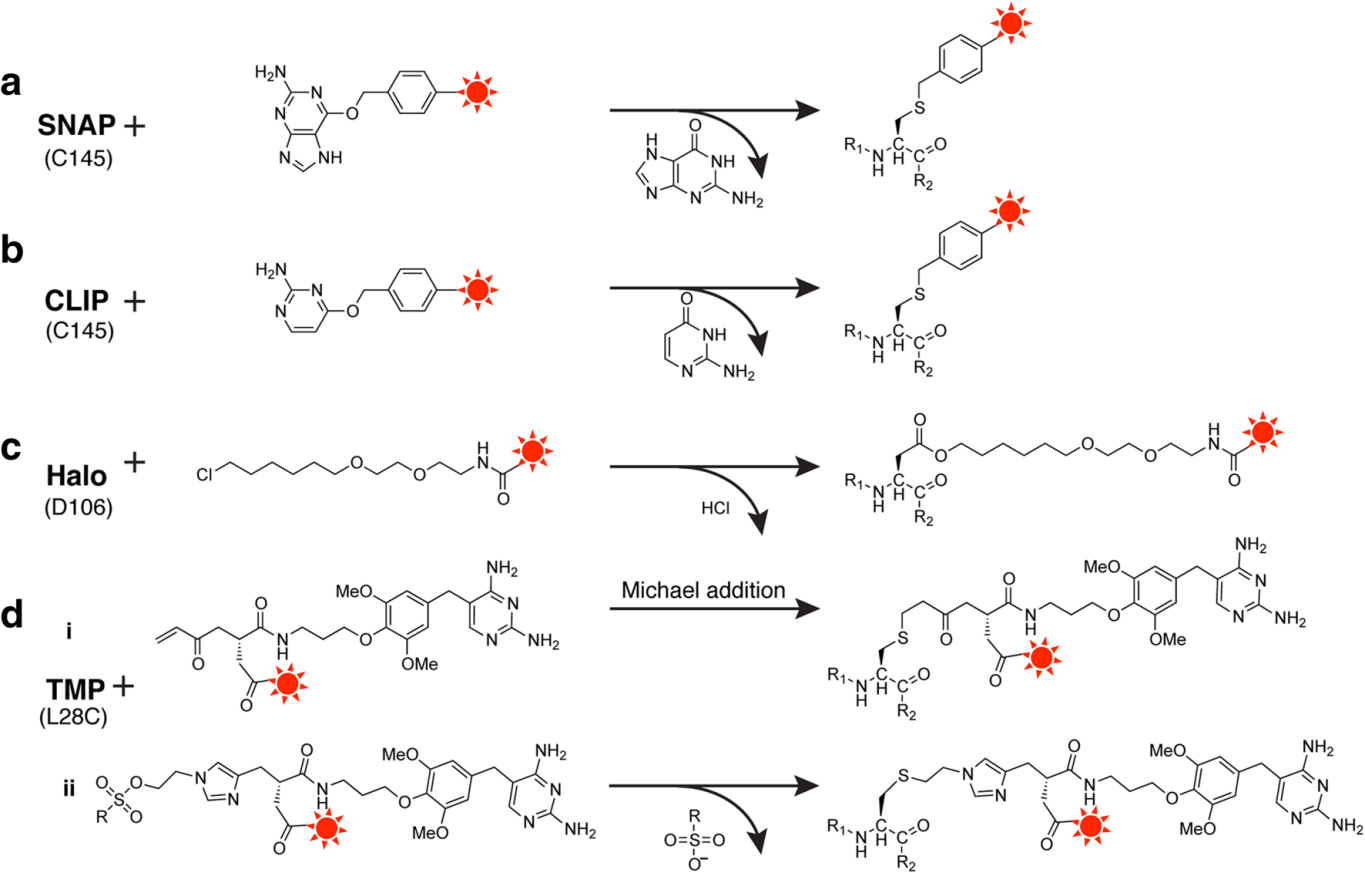
Self-labeling protein tags. **a**, **b** Both SNAP- and CLIP-tag derive from *O* ^6^-methylguanine-DNA methyltransferase with C145 as the active site. **c** The Halo-tag derives from haloalkane dehalogenase whose active site D106 forms an ester bond with the chloroalkane linker. **d** The TMP-tag noncovalently binds with trimethoprim and brings the α, β-unsaturated carbonyl (*i*) or sulfonyl (*ii*) into proximity of the engineered reactive Cys (L28C) (Figure adapted with permission from: Ref. [229]. Copyright (2017) American Chemical Society)

Chemoenzymatic labeling methods exploit the exquisite molecular recognition mechanism between substrates/inhibitors and enzymes to create a new specific covalent linkage between them by engineering enzymes (Fig. 24) [229].

##### SNAP-tag

SNAP-tag (20 kDa) was derived from the human DNA repair protein *O* ^6^-alkylguanine-DNA alkyl-transferase (AGT). The normal function of AGT is to repair *O* ^6^-alkylated guanine in DNA by transferring the alkyl group in an SN2 reaction to a reactive Cys145 residue in AGT. The repair mechanism is unusual because the protein is irreversibly inactivated. Consequently, the reaction of AGT-fusion proteins with *O* ^6^-benzylguanine (BG) derivatives harboring functional moieties leads to the irreversible and covalent labeling of the fusion proteins since the functional moieties on BG are transferred along with the benzyl group of BG to the reactive Cys, creating a stable thioether covalent bond. The SNAP-tag-mediated labeling of proteins in bacteria and yeast is specific, since the respective endogenous AGTs do not accept BG as substrates, whereas AGT-deficient cell lines should be used for labeling in mammalian cells [258].

##### CLIP-tag

Subsequently, AGT mutant-based CLIP-tag, which reacts specifically with *O* ^2^-benzylcytosine (BC) derivatives, was developed by directed evolution. To generate a mutant library of AGT, AA residues at positions with indirect proximity to BG bound in the active site were chosen with the aid of the crystal structure of wild-type AGT. After two-step library screenings using yeast and phage display, CLIP-tag, the eight-point mutant of AGT (Met60Ileu, Tyr114Glu, Ala121Val, Lys131Asn, Ser135Asp, Leu153Ser, Gly157Pro, Glu159Leu) was selected. CLIP-tag with potent catalytic activity exhibited a 105-fold change in substrate specificity and a 100-fold greater preference for BC over BG [259]. The mutual orthogonality of the SNAP- and CLIP-tags enables the simultaneous labeling of multiple proteins in the same cellular context.

##### HaloTag

*Rhodococcus* haloalkane dehalogenase (DhaA) removes halides from aliphatic hydrocarbons by a nucleophilic displacement mechanism. A covalent ester bond is formed during catalysis between an Asp106 residue in the enzyme and the hydrocarbon substrate. The base-catalyzed hydrolysis of this covalent intermediate subsequently releases the hydrocarbon as an alcohol and regenerates the Asp106 nucleophile for additional rounds of catalysis. The based-catalyzed cleavage is mediated by a conserved His272 residue located near the Asp106 nucleophile. HaloTag (33 kDa) was derived from a mutant DhaA, whose catalytic His272 residue is substituted with a Phe residue and does not exhibit the enzymatic activity of intermediate cleavage. However, the apparent binding rates of haloalkanes to this mutant are low compared to those of common affinity-based interactions, such as biotin–streptavidin, potentially hampering the practical utility of this mutant as a protein tag. To overcome this issue, several variants with dramatically improved binding rates were identified using a semi-rational strategy, protein–ligand binding complex modeling, site-saturation mutagenesis, and HTS for faster binding kinetics. A mutant with three point substitutions, Lys175Met/Cys176Gly/Tyr273Leu, i.e., HaloTag, has a high apparent second-order rate constant, thus allowing the labeling reaction to reach completion even under low haloalkane ligand concentrations [260]. Covalent bond formation between the HaloTag and chloroalkane linker (14 atoms long with 6 carbon atoms proximal to the terminal chlorine) functionalized with small synthetic molecules is highly specific, occurs rapidly under physiological conditions and is essentially irreversible. Therefore, the HaloTag-fused protein can be covalently labeled with a variety of functional group-modified chloroalkane linkers and can be applied to a wide range of fluorescent labels, affinity handles, or solid supports.

##### Trimethoprim (TMP)-tag

TMP-tag (18 kDa) was derived from *E. coli* dihydrofolate reductase (eDHFR), which binds the small-molecule inhibitor TMP with high affinity (∼1 nM *K*_D_) and selectivity (affinities for mammalian DHFRs are *K*_D_ > 1 μM). The first-generation TMP-tag harnessed the high-affinity interaction between eDHFR and TMP to form long-duration and yet reversible binding without covalent bond formation. The second-generation, engineered, self-labeling TMP-tag (Leu28Cys) exploited a proximity-induced Michael addition reactivity between a Cys28 residue engineered on the eDHFR surface near the TMP binding site and a mild electrophile, such as an α, β-unsaturated carbonyl moiety, e.g., the β-carbon of acrylamide, or a sulfonyl group installed on the TMP derivatives. To optimize the positioning of the Cys residue nucleophile and the acrylamide electrophile of the TMP derivatives, the site of point mutation on the eDHFR surface and the atom length of the spacer between the 4′-OH group of the TMP and the reactive β-carbon of the acrylamide functional group were investigated based on the molecular modeling of the eDHFR and TMP derivative complexes. After subsequent combinatorial screening in vitro, the combination of the TMP-tag (Leu28Cys) and the TMP derivatives with a 10-atom spacer was selected and exhibited superior specificity and efficiency in protein labeling with fluorophores for live cell imaging [261]. Since the covalent TMP-tag is based on a modular organic reaction rather than a specific enzyme modification, it is easier to build additional features into the covalent TMP-tag.

Self-labeling protein tags, such as SNAP-, CLIP-, Halo- and TMP-tags, feature exquisite specificity and broad applicability to the areas of subcellular protein imaging in live cells, the fabrication of protein–DNA, protein–peptide and protein–protein complexes, and protein immobilization on solid materials, but they are limited by their large molecular size (20–30 kDa) and expensive substrate derivatives, except for HaloTag.

### Linker engineering

Linker engineering is also an important technology for controlling the distances, orientations and interactions among functional components crosslinked in conjugates. Linkers are indispensable units for the fabrication of multidimensional biomaterials or complexes of bio/organic/inorganic materials. Such linkers can be classified as chemical or biological linkers, such as oligonucleotides or polypeptides.

#### Chemical linkers

Chemical linkers have been widely used to modify or crosslink biomolecules, such as proteins, peptides, nucleic acids and drugs, synthetic polymers and solid surfaces with functional molecules and materials. Chemical linkers can be characterized by the following properties: chemical specificity, reactive groups, spacer arm length, water solubility, cell membrane permeability, spontaneously reactive or photoreactive groups, and cleavability by such stimuli as pH, redox, and light. Particularly, spacer arm length and water solubility are important parameters for protein modifications and crosslinking using chemical linkers. For example, when biomolecules are functionalized with small molecules, such as fluorophores or bioorthogonal functional groups, rigid, short methylene arms are utilized as spacers. Various photocleavable, short chemical linkers were also developed to control the functions of crosslinked biomolecules [54, 262, 263]. In contrast, when proteins are functionalized with hydrophobic or large materials, hydrophilic, flexible, long spacer arms formed from PEG chains are often utilized to increase the water solubility of functionalized chemical linkers and to avoid steric hindrance between proteins and functionalized materials. We utilized PEG chains as chemical linkers to prepare a Fab’-green fluorescent protein (GFP) immunoconjugate for a homogeneous immunoassay [264], an enzyme-streptavidin conjugate for enzyme activity control [265, 266], and a *Synechocystis* sp. DnaB intein-TMP conjugate for in vitro protein ligation [267], and the results showed that the length of the PEG chemical linkers affected both the conjugation efficiency and the controllability of protein function. We also produced antibody-lipid and peptide-lipid conjugates for cell surface display [268–270] using PEG chain linkers. Although there are enormous bioconjugation applications for biomolecules using chemical linkers, the details of recent applications are reviewed elsewhere [271–279].

#### Biological linkers

##### Oligonucleotide linkers

In the bottom-up fabrication of nanoscale systems, synthetic DNA oligonucleotides are extraordinarily useful as a construction unit. The extremely high specificity of Watson–Crick base pairing allows one to readily design DNA linkers by using the predictable adenine–thymine (A–T) and guanine–cytosine (G–C) hydrogen-bonding interaction between complementary nucleic acids. In practice, short DNA oligomers with approximately 10–30 nucleotides (mostly 21 nucleotides forming a 7-nm long base pair segment) have been utilized as linkers to noncovalently conjugate complementary oligonucleotide-modified materials by hybridization and facilitate the fabrication of a wide variety of programed structures [117–120, 280]. These DNA linkers have been utilized to immobilize functional materials (e.g., DNA, aptamers, peptides, proteins, antibodies, enzymes, and NPs) on complementary DNA-modified solid supports for bioanalysis [117, 281], to fabricate multifunctional NPs for biosensing and bioimaging [65, 68, 77, 79], for DNA origami, and for placing cascading multienzyme complexes on DNA scaffolds [120, 122–125]. Although short DNA linkers display a relatively high physicochemical stability in vitro, some approaches, such as the utilization of unnatural base DNA or PNA, are required for in vivo applications to prevent degradation by nucleases.

**Fig. 25:**
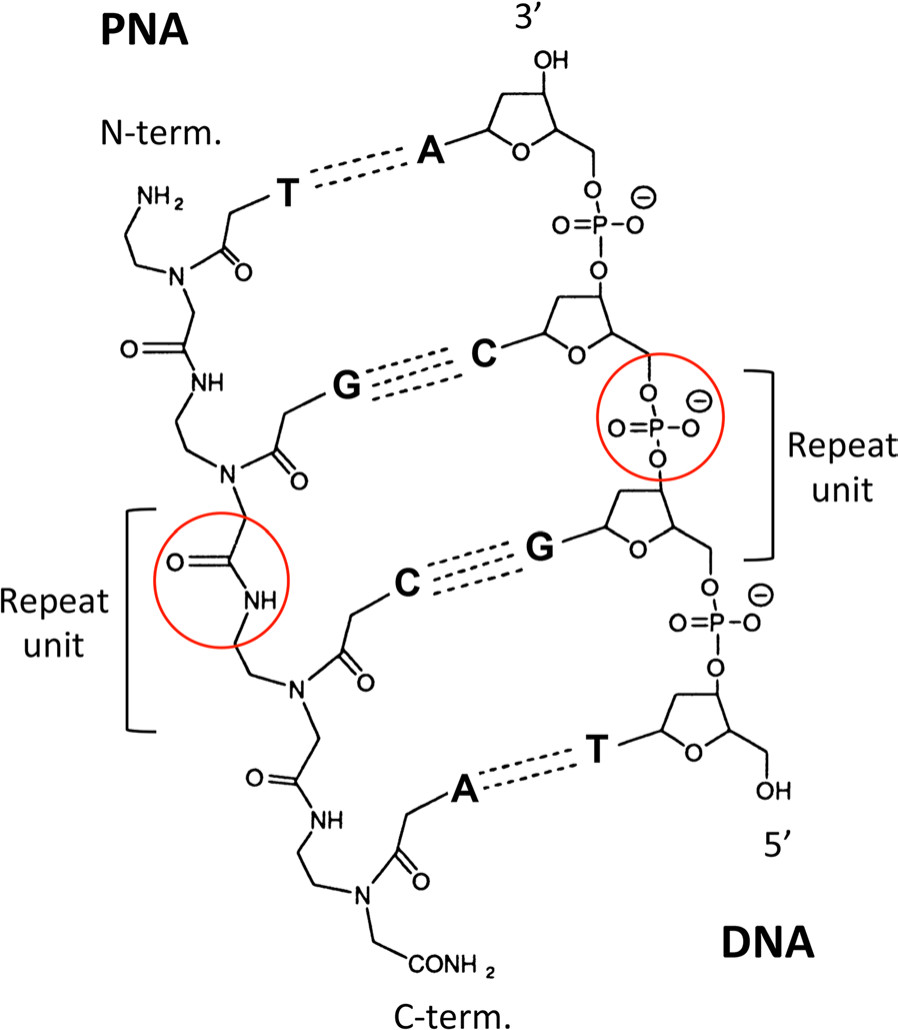
Schematic chemical structures of PNA and DNA. The *circles* show the different backbone linkages of PNA and DNA. A, T, G, and C denote adenine, thymine, guanine and cytosine, respectively

PNA is a DNA analog with a noncyclic, peptide-like backbone (Fig. 25). Owing to its flexible and neutral backbone instead of a negatively charged deoxyribose phosphate backbone, PNA exhibits very good hybridization properties with DNA, RNA, PNA, and DNA duplexes at low and even high ion concentrations, as well as a higher temperature stability than the corresponding pure nucleic acid complexes. Therefore, PNA can highly discriminate mismatched DNA and has a stronger binding affinity for complementary DNA than does its DNA counterpart. PNA also displays a very high stability against enzymatic degradation due to its peptide-like backbone [282]. Applications of PNA linkers in the fields of therapy, diagnosis, and biosensing have been reviewed [282–284]. For example, coupling a radioactively labeled PNA to a TfR mAb rendered the antisense agent transportable through the blood–brain barrier [285]. The identification and differentiation of tuberculous and nontuberculous mycobacteria in liquid cultures were clinically evaluated by a fluorescence hybridization assay using PNA [286]. The detection of a complementary oligonucleotide at a femtomolar (~10^−15^ M) level was accomplished based on the ion-channel sensor technique using Au electrodes modified with self-assembled monolayers of the PNA probe and 8-amino-1-octanethiol [287].

##### Three classes of peptide linkers

The concepts of the protein domains and modules were first proposed in 1973 by Wetlaufer [288] and 1981 by Go [289], respectively. These concepts gave insights into domains and modules as the basic structural, functional or evolutionary units of proteins. A wide variety of naturally occurring multidomain fusion proteins with different architectures have been generated through evolution and characterized to meet the functional requirements of living organisms at the molecular level [290]. The strategies used by nature to evolve fusion proteins have been mimicked by the construction of hybrid or chimeric proteins using molecular biology techniques. Inspired by natural fusion proteins, synthetic fusion proteins have been designed to achieve synergistically improved bioactivities or to generate novel functional combinations derived from each of their component moieties, which are integrated into one molecule by peptide linkers. The fusion proteins have been widely applied in various areas, including recombinant protein production by the tag-mediated enhancement of protein expression, solubility and high-throughput purification [291, 292], fluorescent protein-mediated molecular imaging [293], advanced biocatalysis [101, 108, 111, 115, 164, 290, 294–297], biosensing and bioelectronic materials [290, 298–300], pharmaceuticals, diagnostics and therapeutics [208, 290, 301, 302], reporter protein-mediated immunoassays [303–310], the chimeric receptor-mediated control of cell fate, e.g., growth, death, migration or differentiation [311–319], the library selection of antibodies [203, 320, 321] and antibody-mediated drug delivery [218, 322, 323].

Genetic fusion and enzymatic conjugation technologies have been commonly adopted for the construction of fusion proteins. Among them, an end-to-end genetic fusion is the simplest method for constructing a fusion protein, where the coding genes of functional units are combined together and expressed in a suitable host organism. Direct tandem genetic fusion through restriction enzyme sites is simple; the flexible and unstructured N- or C-terminal regions of the component proteins and additional short peptides derived from restriction enzyme sites act as a peptide linker to provide enough space between the functional units of a fusion protein for correct folding. However, if the N- or C-terminus is not flexible or not long enough to prevent steric hindrance, this effect will reduce the degrees of freedom of units in fusion protein dynamics and may cause unfavorable results, such as inclusion body formation derived by protein misfolding, a loss of function and a low yield of functional fusion proteins. For this reason, longer peptide linkers are generally inserted between functional units [290].

Peptide linkers are generally classified into three groups according to their structures: flexible linkers, rigid linkers, and site-specific linkers cleavable by proteolytic enzyme digestion. In addition to the basic role of linking functional units together or releasing functional units (e.g., toxin release in drug delivery systems, affinity tag cleavage from tag-fused recombinant pharmaceutical proteins in the purification process), peptide linkers may offer many other advantages for the production of fusion proteins, such as improving biological activity and structural stability and achieving desirable biopharmaceutical pharmacokinetic profiles [324]. Therefore, peptide linkers play a variety of structural and functional roles in fusion proteins.

##### Flexible peptide linkers

Flexible linkers are frequently adopted as natural inter-domain peptide linkers in multidomain proteins when the joined domains require a certain degree of movement or interaction. Based on the analysis of AA preferences for residues contained in these natural flexible linkers, it has been revealed that they are generally composed of small, nonpolar (e.g., Gly) or polar (e.g., Ser, Thr) residues [325]. The small size of these AA residues provides flexibility and enables the mobility of the connected functional units. The incorporation of Ser or Thr can maintain the stability of the peptide linker in aqueous solutions by forming hydrogen bonds with water molecules, thereby reducing unfavorable interactions between the linker and protein moieties. The most widely used synthetic flexible linker is the G_4_S-linker, (G_4_S)_n_, where n indicates the number of G_4_S motif repeats. By changing the repeat number “n,” the length of this G_4_S linker can be adjusted to achieve appropriate functional unit separation or to maintain necessary interactions among units, thus allowing proper folding or achieving optimal biological activity [324]. Poly-Gly (G_n_) linkers also form an elongated structure similar to that of the unstable 3_10_-helix conformation. Since Gly has the greatest freedom in backbone dihedral angles among the natural AAs, G_n_ linkers can be assumed to be the most “flexible” polypeptide linkers [326]. In addition to the G_4_S linkers and poly-Gly linkers, many other flexible linkers, such as KESGSVSSEQLAQFRSLD and EGKSSGSGSESKST for the construction of a single-chain variable fragment (scFv), have been designed by searching libraries of 3D peptide structures derived from protein data banks for crosslinking peptides with proper V_H_ and V_L_ molecular dimensions [327]. These flexible linkers are also rich in small or polar AAs, such as Gly, Ser, and Thr, and they contain additional AAs, such as Ala, to maintain flexibility, as well as large polar AAs, such as Glu and Lys, to increase the solubility of fusion proteins.

##### Rigid peptide linkers

Rigid linkers act as stiff spacers between the functional units of fusion proteins to maintain their independent functions. The typical rigid linkers are helix-forming peptide linkers, such as the poly-proline (Pro) helix (P_n_), poly-Ala helix (A_n_) and α-helix-forming Ala-rich peptide (EA_3_K)_n_, which are stabilized by the salt bridges between Glu^−^ and Lys^+^ within the motifs [328]. Fusion proteins with helical linker peptides are more thermally stable than are those with flexible linkers. This property was attributed to the rigid structure of the α-helical linker, which might decrease interference among the linked moieties, suggesting that changes in linker structure and length could affect the stability and bioactivity of functional moieties.

The Pro-rich peptide (XP)_n_, with X designating any AA, preferably Ala, Lys, or Glu, can also constrain the linker to an extended conformation with relatively limited flexibility. The Pro residue is a very unique AA; it is a cyclic AA, and its side chain cyclizes back to the amide on the backbone, which restricts the confirmation of its backbone to a small range of backbone angles. Since the Pro residue has no amide hydrogen to form a hydrogen bond with other AAs, it can avoid ordered structures and prevent interactions between the linkers and neighboring domains [324, 329]. Therefore, Pro resides in (XP)_n_ linkers can increase the linker stiffness and effectively separate neighboring domains.

##### Site-specific, cleavable peptide linkers

Genetic fusion technology provides an effective means for recombinant protein expression and purification. A comprehensive review of affinity tags can be found elsewhere [292, 330]. Examples of affinity tags include poly-His, FLAG, HA, strep II, the calmodulin-binding peptide and the chitin-binding domain. These tags specifically interact with their partner molecules and allow the fused protein to be captured by corresponding partner molecule-modified matrices. In most cases, the tags are removed from the fusion proteins after an affinity tag-assisted purification process to obtain the final product consisting of pure target protein. This is usually achieved by enzymatic or chemical cleavage at the junction between the tag and the target protein. Endoproteases commonly used to cleave fusion tags include factor Xa (I(E/D)GR↓X), enterokinase (DDDDK↓X), thrombin (LVPR↓GS), tobacco etch virus protease (ENLYFQ↓(G/S)) and a genetically engineered derivative of human rhinovirus 3C protease, PreScission™ (LEVLFQ↓GP) [329, 330]. Chemicals that are specific and efficient for the chemical cleavage of proteins in solution are CNBr (Met↓), 2-(2′-nitrophenylsulfonyl)-3-methyl-3-bromoindolenine (Trp↓), 2-nitro-5-thiocyanobenzoic acid (Cys↓), formic acid (Asp↓Pro) and hydroxylamine (Asn↓Gly) [331]. Here, the down arrow and X in parenthesis indicate the cleavage site of the recognition site and any AA, respectively. In general, enzymatic cleavage is site-specific and can be carried out under mild conditions. However, cleavage efficiency may vary with different fusion proteins. Steric hindrance or the presence of unfavorable residues around the cleavage site could result in inefficient processing. In contrast to enzymatic cleavage, chemical cleavage offers a less expensive alternative but requires harsh conditions that may cause side-chain modifications. Furthermore, since chemical cleavage usually targets specific residues or dipeptide linkages, the frequent presence of the single- or double-residue site recognized by these chemicals within the AA sequence of the target protein limits its use [332].

Self-cleaving tags are a special group of fusion tags that are based on protein modules (e.g., intein, SrtA, the FrpC module, and the Cys protease domain) and possess inducible proteolytic activities. Fusion proteins containing them can be site-specifically self-cleaved by the trigger of a low molecular weight compound or a change in its conformation. Combined with appropriate affinity tags, self-cleaving tags enable fusion protein purification, cleavage and target protein separation to be achieved in a single step [332].

**Fig. 26:**
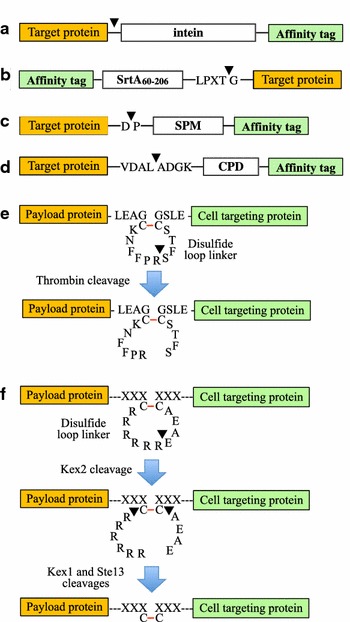
Schematic representation of the construction of self-cleaving fusion systems. *Filled triangle* indicates cleavage sites and X stands for any AA. **a** The construct of the original C-terminal intein fusion in which the target protein is fused to the N-terminus of the CBD-tagged intein. **b** The SrtA fusion construct that contains an N-terminal affinity-tag, SrtA catalytic core, the LPXTG motif and the target protein. Cleavage at the LPXTG site allows the release of the target protein with an extra N-terminal glycine. **c** The FrpC fusion construct that consists of the target protein and the affinity-tagged SPM. Cleavage at the Asp–Pro site (the first two AAs of SPM) results in the release of the target protein with an extra aspartate residue at its C-terminus. **d** The CPD fusion construct in which the affinity-tagged CPD is fused to the C-terminus of the target protein. The VD double residue in the linker sequence comes from the *Sal*I restriction site used for cloning whereas ALADGK are residues contained within the CPD. **e** The dithiocyclopeptide linker with one protease-sensitive site. The fusion protein is linked via a dithiocyclopeptide linker containing a thrombin-specific sequence, PRS. The design of dithiocyclopeptide linker was based on the structure of the cyclopeptide, somatostatin, with the replacement of AA residues 8–10, WKT, by a thrombin-specific cleavage sequence, PRS. **f** The dithiocyclopeptide linker with three secretion signal processing protease-sensitive sites. The fusion protein is linked via a dithiocyclopeptide linker containing Kex1, Kex2 and Ste13-specific cleavage sequences. Kex2 cleaves RR↓E. Kex1 and Ste13 remove C-terminal RR and N-terminal EA, respectively

In the case of Intein-tag, the target protein is fused to the N-terminus of intein [e.g., VMA intein from *Saccharomyces cerevisiae* (51 kDa) or DnaB intein from *Synechocystis* sp. strain PCC6803 (17 kDa)], whose C-terminus is conjugated with an affinity tag (Fig. 26a). Intein-mediated site-specific cleavage can be triggered by thiol reagents, such as dithiothreitol or β-mercaptoethanol.

As for SrtA-tag, the fusion protein consists of an N-terminal affinity tag, a SrtA catalytic core, the LPXTG motif and the target protein (Fig. 26b). On-resin cleavage can be induced by incubation in a Ca^2+^ ion-containing buffer, and the released target protein, with an extra Gly residue at its N-terminus, can then be collected. However, this system has a potential drawback. Although the activity of SrtA from *S. aureus* is inducible by Ca^2+^ ions and moderate conditions, it is not completely suppressed during protein expression because abundant soluble Mg^2+^ ions (10^3^- to 10^4^-fold greater in concentration than Ca^2+^ ions) in the cytosol can partly replace Ca^2+^ ions in function [333], which causes unwanted fusion cleavage at an early stage.

The FrpC module is an iron-regulated protein produced by the gram-negative bacterium *Neisseria meningitides*. The fusion construct contains the target protein, which is at the N-terminal moiety, and the affinity-tagged self-processing module (SPM) (Fig. 26c). The DNA coding sequence for the first four AAs of the SPM, which are Asp-Pro-Leu-Ala, contains an *Nhe*I restriction site that can be used for cloning. The Ca^2+^ ion-addition induces SPM-mediated cleavage, resulting in the release of the target protein with an extra Asp residue at the C-terminus.

*Vibrio cholerae* secretes a toxin with large, multifunctional, auto-processing repeats; this toxin undergoes proteolytic cleavage during translocation into host cells. The proteolysis of the toxin is mediated by a conserved internal Cys protease domain (CPD), which is activated upon the binding of the small molecule inositol polyphosphate (IP6). Affinity-tagged CPD can be fused to the C-terminus of the target protein (Fig. 26d). The IP6-addition triggers CPD-mediated cleavage, which allows the target protein to be released. Depending on the cloning site used, one or more additional residues may be appended to the C-terminus of the target protein.

Other applications of cleavable linkers are drug delivery systems to release free functional units of fusion proteins in vivo. These linkers are designed to cleave under specific conditions, such as the presence of reducing reagents or proteases. This linker system enables fusion proteins to reduce steric hindrance and improve both the independent actions and bioactivities of individual functional units after in vivo cleavage. The reduction of disulfide bonds in vivo has been widely applied for the release of payloads from drug delivery systems fabricated by chemical conjugation technologies. Similarly, disulfide linkers cleavable in vivo were designed for recombinant fusion proteins [334, 335]. One such disulfide linker (LEAG**C**KNFFPR↓SFTS**C**GSLE) is based on a dithiocyclopeptide containing an intramolecular disulfide bond formed between two Cys residues on the linker, as well as a thrombin recognition sequence (PRS) between the two Cys residues (Fig. 26e). Another disulfide linker (**C**RRRRRREAEA**C**) also contains an intramolecular disulfide bond and a peptide sequence sensitive to the secretion signal-processing proteases of the yeast secretory pathway. During protein expression, this linker is first cleaved by the protease Kex2 at CRRRRRR↓EAEAC, followed by the removal of the dipeptides RR and EA by the secretion signal-processing proteases Kex1 and Ste13 (C↓RR↓RR↓RR, EA↓EA↓C), respectively (Fig. 26f). As a result, the AAs between the two Cys residues in the linker were completely removed during secretion, and the disulfide linked fusion protein was directly expressed by *Pichia pastoris*.

##### The effect of linker composition, flexibility/rigidity and length on the functions and conformations of fusion proteins

The folding, stability, proteolytic sensitivity and function of fusion proteins might be affected by the AA composition and the flexibility/rigidity and length of the peptide linkers. For example, fusion proteins consisting of a cellulose-binding domain of *Neocallimastix patriciarum* cellulase A (Cel6A) and lipase B from *Candida antarctica* were constructed by connecting two functional units with different linker peptides (4–44 AA residues, different Asn residue numbers and positions for potential N-glycosylation sites) derived from the natural peptide linker contained in Cel6A. Analyses of linker stability toward proteolysis and the cellulose-binding activity and lipase activity of the fusion proteins were conducted; the results revealed that fusion proteins with shorter linkers (4–16 AA residues) were more stable against proteolysis but had slightly lower cellulose-binding capacities than those containing longer linkers. However, all fusion proteins retained the lipase-specific activity of the wild-type protein [336].

Bifunctional fusion proteins composed of the catalytic domains of endoglucanase (Endo5A) and β-glucosidase (Gluc1C) from a *Paenibacillus* strain were constructed by changing the connection order of two domains and linking them with flexible peptide linkers of different lengths (G_4_S)_n_ (n = 0–3). The results indicated that the substrate affinity *Km* and catalytic efficiency *k*_cat_/*Km* of Gluc1C were sensitive to its position, as it showed a decline in both affinity and catalytic efficiency when Gluc1C was placed at the N-terminus of the fusion protein. However, there was no direct relationship of linker length with either Endo5A or Gluc1C activity [337].

Tandem fusion proteins of human serum albumin and onconase (ONC) with flexible linkers (G_4_S)_n_ (n = 0–3) were constructed and expressed in *P. pastoris*. The expression level of the fusion proteins had no relationship with the linker length. However, while the ONC moiety of the fusion protein without a linker (n = 0) showed no cytotoxicity toward tumor cells, this gradually improved with increasing linker length [338].

For the development of a bifunctional immunoreagent, the B1 domain of *Streptococcal* protein G (SpG), which binds to the Fc region and CH1 domain of IgG, was fused with luciferase from *Vargula hilgendorfii* (Vluc) using flexible peptide linkers (G_4_S)_n_ (n = 0–1). The resulting fusion protein, SpG-(G_4_S)_n_-Vluc, retained the bioluminescence activity of the Vluc moiety but lost the binding affinity of SpG to IgG. However, inserting the three α-helices bundle D domain of protein A from *S. aureus* (SpA) between the SpG and the (G_4_S) linker successfully recovered the binding affinity of SpG to the CH1 domain of IgG [339].

Fusion protein pairs for noncompetitive and homogeneous immunoassays were developed by optimizing the flexible G_4_S linker length of each fusion protein. This assay system is based on the antigen-dependent reassociation of antibody variable regions (V_H_, V_L_) and the subsequent complementation of the β-Gal domains ∆α and ∆ω. The best pair was found to be V_H_-(G_4_S)_2_-∆α and V_L_-(G_4_S)_1_-∆ω, which, at its optimal concentration, showed a 2.5-fold increase in β-Gal activity upon antigen addition [340].

Chimeric receptors (chimeras of anti-fluorescein (FL) scFv and an engineered c-Mpl receptor possessing only signaling mediator STAT3-binding motifs) were designed by changing the peptide linker length between the binding motifs of JAK and STAT3 using flexible linkers (G_4_S)_n_ (n = 0, 3, 6, 9). The activation level of STAT3 was quantitatively evaluated by detecting the level of phosphorylated STAT3 after the stimulation of chimeric receptor-expressing cells with FL-labeled bovine serum albumin (BSA-FL). The results showed that the STAT3 activation levels were 0.8-, 1.5- and 1.4-fold greater with (G_4_S)_3_, (G_4_S)_6_ and (G_4_S)_9_, respectively, than without a linker. Therefore, changes in the distance from the JAK-binding domain to the STAT3-binding motif exerted relatively minor effects on the phosphorylation level of STAT3 [341].

Helical poly-Ala linkers (Ala)_n_ (n = 0–4) were inserted between the transmembrane and intracellular domains of a chimeric receptor (a tandem fusion protein of anti-FL scFv/intracellular domain-truncated EpoR/gp130 intracellular domain), and the effect of linker length on cell proliferation was investigated by stimulating chimeric receptor-expressing cells with BSA-FL. A periodic enhancement in cell proliferation was induced by the insertion of one to four Ala residues. The chimeric receptors with linkers (Ala)_n_ (n = 0, 1) transduced a growth signal, while growth activity was lost when (Ala)_n_ (n = 2–4) linkers were inserted. Furthermore, the extracellular EpoR D1 domain-truncated chimeric receptor showed different patterns in the periodic enhancement of cell proliferation by the insertion of one to four Ala residues. In this case, the chimeric receptors with linkers (Ala)_n_ (n = 0, 3, 4) failed to transduce a growth signal, whereas growth activity was restored when one or two Ala residues were inserted. These results clearly demonstrate the importance of intracellular domain orientation for the activation of chimeric receptors, which is readily controlled by the 109° rotation of the α-helix Ala linker with each increment of one Ala residue [342].

To construct a ligand-inducible scFv dimer, anti-ErbB2 scFv was fused with FKBP_F36V_, which is a mutant of FK-binding protein 12 that can be dimerized by the synthetic homodimeric ligand AP20187. The three kind of linkers, i.e., flexible (G_4_S)_3_, rigid α-helix (EA_3_K)_3_ and DKTHCP(G_4_S)_2_, derived from the hinge region of IgG were inserted between scFv and FKBP_F36V_, and the effect of linker properties on the activity of the fusion protein dimer, which can dimerize the artificial chimeric receptor ErbB2-gp130 expressed on the cell surface and induce cell proliferation signaling from the dimerized chimeric receptor, were investigated. The results showed that the fusion protein with the hinge linker was the best for activating ErbB2-gp130 chimera-induced cell proliferation [320].

**Fig. 27:**
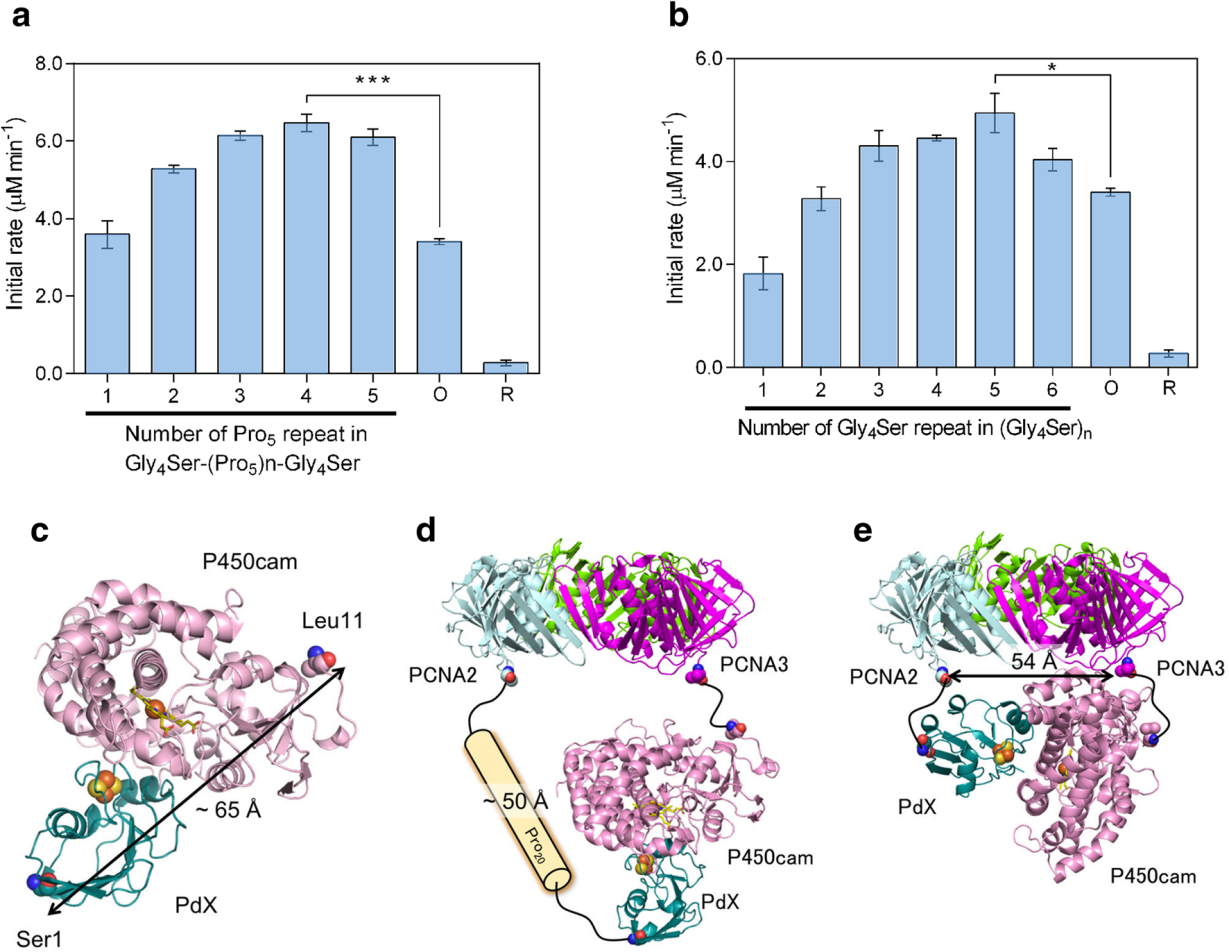
Optimization of the PCNA2-PdX fusion protein linker in PUPPET. **a** P450cam oxidation activities of the PUPPET linker variants, PUPPET-Pn (n = 1–5). **b** P450cam oxidation activities of the PUPPET linker variants, PUPPET-Gn (n = 1–6). **c** A docking model of P450cam and PdX. **d** Spatial arrangement of P450cam and the PCNA ring when the PdX-binding site of P450cam faces in the same direction to the PCNA ring. **e** Spatial arrangement of P450cam and the PCNA ring when the PdX-binding site of P450cam faces in a perpendicular direction to the PCNA ring (Figures reproduced from Ref. [343])

It has been demonstrated that the selective complex formation of P450cam with its redox partner proteins, PdX and PdR, can be achieved by fusing each component to the C-terminus of a different subunit of the heterotrimer PCNA from *Sulfolobus solfataricus* to form a self-assembling scaffold [111]. To enhance the activity of this self-assembled multienzyme complex, the peptide linker connecting PdX with PCN2 was optimized using various peptide linkers, such as flexible linkers (G_4_S)_n_ (n = 1–6), helical and rigid Pro-rich linkers (G_4_S–(P_5_)_n_–G_4_S) (n = 1–5) and other linkers (G_4_S–LVPRGS–G_4_S). Although the activity was affected by the lengths of both the rigid Pro-rich linkers and the flexible linkers, the Pro-rich linkers provided the greatest activity enhancement. The optimized Pro-rich linker (G_4_S–(P_5_)_4_–G_4_S) enhanced the activity by 1.9-fold compared with the G_4_S–LVPRGS–G_4_S linker, while the (G_4_S)_n_ (n = 1–6) linker did not yield activity higher than the maximum activity of the optimized Pro-rich linker. Both peptide linker rigidity/flexibility and length were found to be important for enhancing overall multienzyme complex activity (Fig. 27) [343].

The tandem fusion proteins β-glucanase (Gluc)/xylanase (Xyl) were constructed using peptide linkers, such as flexible linkers (G_4_S)_n_ (n = 0–3), α-helical linkers (EA_3_K)_n_ (n = 0–3) and others (MGSSSN designed using the software of the web server LINKER [344], and TGSRKYMELGATQGMGEALTRGM derived from the two α-helix bundle of *Humicola insolens* endocellulase). The effects of the linkers on the thermal stability and catalytic efficiency of both enzymes were analyzed. The Gluc moieties of most fusion constructs showed greater stability at 40–60 °C than did the parental Gluc and the linker-free fusion protein. All the Xyl moieties showed thermal stabilities similar to that of the parental Xyl, at ≥60 °C. It was also revealed that the catalytic efficiencies of the Gluc and Xyl moieties of all the fusion proteins were 3.04- to 4.26-fold and 0.82- to 1.43-fold those of the parental moieties, respectively. The flexible linker (G_4_S)_2_ resulted in the best fusion proteins, whose catalytic efficiencies were increased by 4.26-fold for the Gluc moiety and by 1.43-fold for the Xyl moiety. The Gluc and Xyl moieties of the fusion protein with the rigid linker (EA_3_K)_3_ also showed 3.62- and 1.31-fold increases in catalytic efficiency [345].

**Fig. 28:**
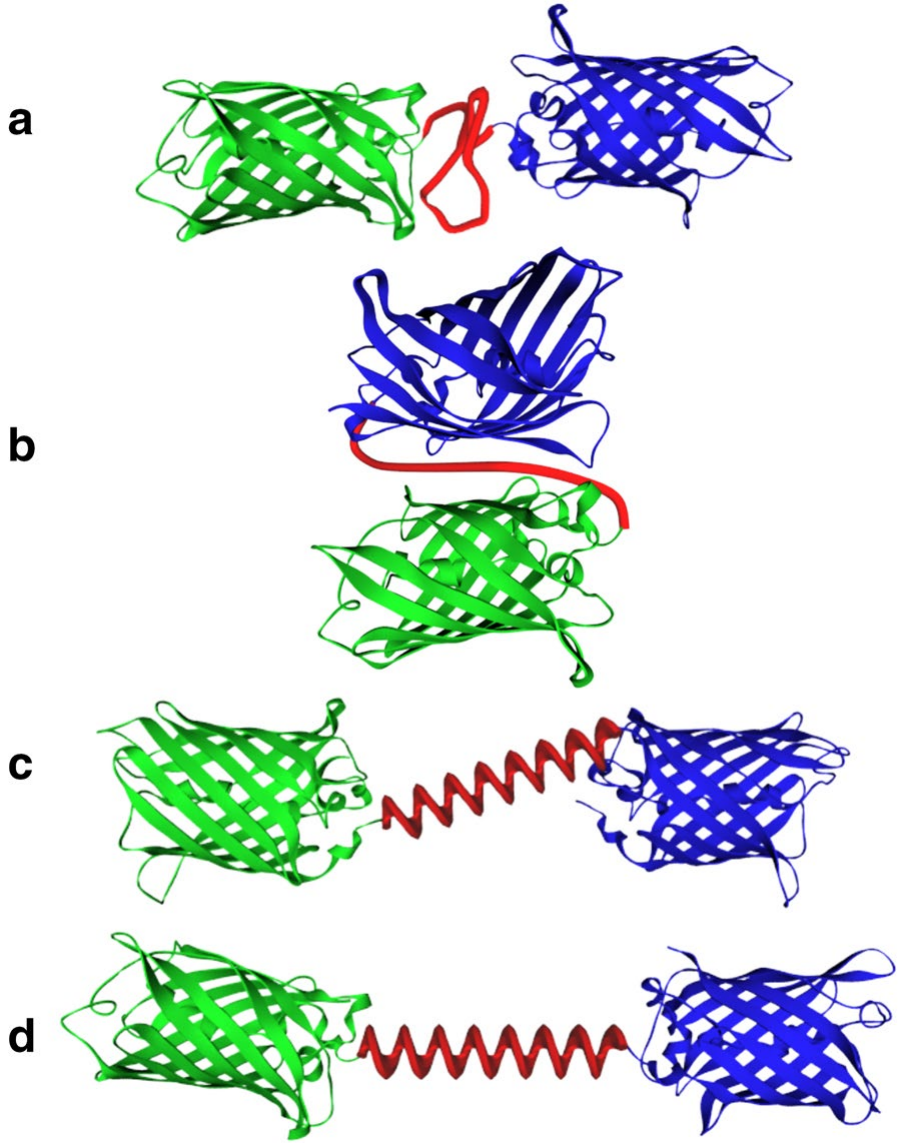
Schematic illustrations of various conformations of the fusion proteins. **a** EBFP (*blue*) and EGFP (*green*) are situated in a straight line, with the flexible linker (*red*) between the two domains. **b** EBFP and EGFP reside side by side, for the most compact conformation with the flexible linker. **c** The helical linker connects EBFP and EGFP diagonally. **d** The helical linker and the long axes of EBFP and EGFP are situated in a straight line (Figure adapted with permission from: Ref. [347]. Copyright (2004) John Wiley & Sons)

**Fig. 29:**
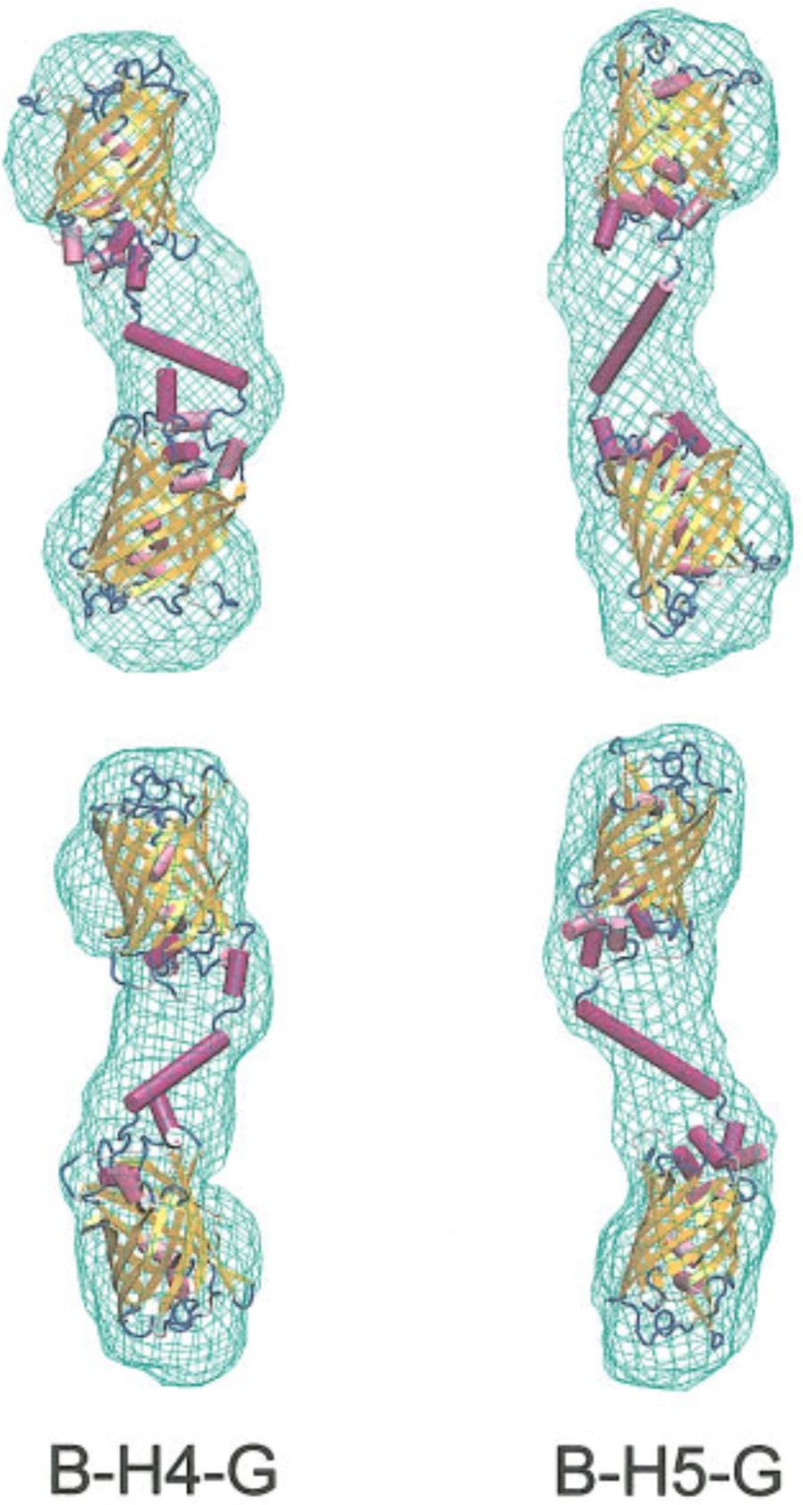
High-resolution models (cartoon representation) of the EBFP and EGFP connected with the helical linkers. B–H4–G and B–H5–G indicate EBFP–LA(EA_3_K)_n_AAA–EGFP (n = 4, 5), respectively. Low-resolution models based on only SAXS data are shown as wire-frames. The linker and the two domains are modeled and two different views are shown (Figure reproduced with permission from: Ref. [347]. Copyright (2004) John Wiley & Sons)

Aiming to clarify the criteria for designing peptide linkers for the effective separation of the domains in a bifunctional fusion protein, a systematic investigation was carried out. As a model, the fusion proteins of two *Aequorea* GFP variants, enhanced GFP (EGFP) and enhanced blue fluorescent protein (EBFP), were employed. The secondary structure of the linker and the relative distance between EBFP and EGFP were examined using circular dichroism (CD) spectra and fluorescent resonance energy transfer (FRET), respectively. The following AA sequences were designed and utilized as peptide linkers: a short linker (SL); LAAA (4 AAs) (derived from the cleavage sites for *Hind*III and *Not*I); flexible linkers (G_4_S)_n_AAA (n = 3, 4); α-helical linkers LA(EA_3_K)_n_AAA (n = 3–5); and a three α-helix bundle from the B domain of SpA (LFNKEQQNAFYEILH LPNLNEEQRNGFIQSLKDDPSQSANLLAEAKKLNDAQAAA). The differential CD spectra analysis suggested that the LA(EA_3_K)_n_AAA linkers formed an α-helix and that the α-helical contents increased as the number of the linker residues increased. In contrast, the flexible linkers formed a random, coiled conformation. The FRET from EBFP to EGFP decreased as the length of the helical linkers increased, indicating that distances increased in proportion to the length of the linkers. The results showed that the helical linkers could effectively separate the neighboring domains of the fusion protein. In the case of the fusion proteins with the flexible linkers, the FRET efficiency was not sensitive to linker length and was highly comparable to that of the fusion proteins with the SL, although the flexible linkers were much longer than the SL, again indicating that the flexible linkers had a random, coiled conformation [346]. The real in situ conformations of these fusion proteins and structures of the linkers were further analyzed using synchrotron X-ray small-angle scattering (SAXS). The SAXS experiments indicated that the fusion proteins with flexible linkers assume an elongated conformation (Fig. 28a) rather than the most compact conformation (Fig. 28b) and that the distance between EBFP and EGFP was not regulated by the linker length. On the other hand, fusion proteins with helical linkers [LA(EA_3_K)_n_AAA n = 4, 5] were more elongated than were those with flexible linkers, and the high-resolution models (Fig. 29) showed that the helical linkers connected the EBFP and EGFP domains diagonally (Fig. 28c) rather than longitudinally (Fig. 28d). However, in the case of the shorter helical linkers (n = 2, 3, especially n = 2), fusion protein multimerization was observed. Since most residues of the short helical linkers are situated closer to the two domains of the fusion protein, the charged residues, Glu and Lys in the (EA_3_K) unit are likely to form ion pairs with the oppositely charged residues on the top surfaces of EBFP and EGFP. Consequently, this ion pairs formation causes destabilization of the short helix and melted helix linkers may act as attractants for the attachment of neighboring molecules due to their charges and hydrophobicity, thereby causing multimerization of fusion protein. On the other hand, in the case of the fusion protein with the longer helical linkers (n = 4, 5), the linkers retained the α-helix structure and could solvate monomeric fusion proteins. These results clearly suggested the outstanding ability of the rigid helical linkers to control the distance and reduce the interference between the domains [347]. This study is the first example of modeling in situ fusion protein conformations and linker structures by combining SAXS data of fusion proteins, structural information of the functional units from the Brookhaven Protein Data Bank (PDB), and molecular dynamics calculations of peptide linker structures. Recently, this modeling method was applied to evaluate the in situ conformations and structures of fusion proteins composed of a de novo two-helix bundle protein and a single trimeric foldon domain of fibritin from the bacteriophage T4 connected by a short peptide linker (KLAAA). Size exclusion chromatography, multi-angle light scattering, analytical ultracentrifugation, and SAXS analyses indicated that the small (S form), middle (M form), and large (L form) forms of the fusion protein oligomers exist as 6-, 12-, and 18-mers, respectively. The SAXS data further suggested that the S and M forms have barrel- and tetrahedron-like shapes, respectively [348].

The selection of a suitable peptide linker, which allows a desirable conformation and interaction among functional units in fusion proteins, is key to the successful design of fusion proteins. Generally, rigid linkers exhibit relatively stiff structures by adopting α-helical structures or by containing multiple Pro residues with the *cis* isomer of the peptide bond. Under many circumstances, they can separate the functional domains in fusion protein more efficiently than do flexible linkers. The length of the linkers can be easily adjusted by changing the linker-unit repeat-number, such as (EA_3_K), to achieve an optimal distance between functional units. As a result, when the spatial separation of the functional units is critical to avoid steric hindrance and to preserve the folding, stability and activity of each unit in the fusion proteins, rigid linkers would be selected. However, there are other types of fusion proteins, in which functional units are required to have a certain degree of movement/interaction or a precise proximal spatial arrangement and orientation to form complexes. In such cases, flexible linkers are often chosen because they can serve as a passive linker to maintain a distance or to adjust the proximal spatial arrangement and orientation of functional units. However, optimizing the peptide linker sequence and predicting the spatial linker arrangement and orientation are more difficult for flexible linkers than for rigid linkers. Current strategies are mostly empirical and intuitive and have a high uncertainty. Therefore, computational simulation technologies for predicting fusion protein conformations and linker structures would potentially encourage rational flexible linker design with improved success rates.

##### Rational algorithms and software for designing linker sequences and structures

The rational design of fusion proteins with desired conformations, properties and functions is a challenging issue. Most current approaches to linker selection and design processes for fusion proteins are still largely dependent on experience and intuition; such selection processes often involve great uncertainty, particularly in the case of longer flexible linker selection, and many unintended consequences, such as the misfolding, low yield and reduced functional activity of fusion proteins may occur. This is mostly because of our limited understanding of the sequence–structure–function relationships in these fusion proteins. To overcome this problem, the computational prediction of fusion protein conformation and linker structure can be considered a cost-effective alternative to experimental trial-and-error linker selection. Based on the structural information of individual functional units and linkers (either from the PDB or homology modeling), considerable progress has been made in predicting fusion protein conformations and linker structures [290]. Approaches for the design or selection of flexible linker sequences to connect two functional units can be categorized into two groups. The first group comprises library selection-based approaches, in which a candidate linker sequence is selected from a loop sequence library without consideration of the conformation or placement of functional units in the fusion proteins. The second group comprises modeling-based approaches, in which functional unit conformation and placement and linker structure and AA composition would be optimized by simulation.

Regarding the first approach, a computer program called LINKER was developed. This web-based program (http://astro.temple.edu/feng/Servers/BioinformaticServers.htm) automatically generated a set of peptide sequences based on the assumption that the observed loop sequences in the X-ray crystal structures or the nuclear magnetic resonance structures were likely to adopt an extended conformation as linkers in a fusion protein. Loop linker sequences of various lengths were extracted from the PDB, which contains both globular and membrane proteins, by removing short loop sequences less than four residues and redundant sequences. LINKER searched its database of loop linker sequences with user-specified inputs and outputted several candidate linker sequences that meet the criteria. The basic input to the program was the desired length of the linker, expressed as either the number of residues or a distance in angstroms. Additional input parameters included potential cleavage sites for restriction endonucleases or proteases to avoid such that the selected linkers would be resistant against the restriction enzymes and the specified protease during the DNA cloning and protein purification process, respectively. The users could also include AA composition preferences (e.g., eliminate bulky hydrophobic residues) to further select their linkers of interest. The output of LINKER included a list of peptide sequences with the specified lengths, sequence characteristics and chemical features of every linker sequence shown by hydrophobicity plots [344, 349]. However, although the PDB database has expanded tremendously during the last decade, no further updates or improvements were made to the LINKER website since it was created, and it is no longer accessible.

The web-based program LinkerDB (http://www.ibi.vu.nl/programs/linkerdbwww/) also provides a database containing linker sequences with various confirmations and a search engine. The search algorithm accepts several query types (e.g., PDB code, PDB header, linker length, secondary structure, sequence or solvent accessibility). The program can provide the linker sequences fitting the searching criteria as well as other information, such as the PDB code and a brief description of the source protein, the linker’s position within the source protein, linker length, secondary structure, and solvent accessibility. Users can search for sequences with desired properties and obtain candidate sequences from natural multidomain proteins [329].

Another server website for facilitating linker selection and fusion protein modeling is SynLinker (http://bioinfo.bti.a-star.edu.sg/linkerdb). It contains information regarding 2260 linkers, consisting of natural linkers extracted from multidomain proteins in the latest PDB, as well as artificial and empirical linkers collected from the literature and patents. A user may specify multiple query criteria to search SynLinker, such as the PDB ID of the source proteins, protein names, the number of AA residues in a linker, and/or the end-to-end distance of a linker conformation in Angstroms (Å). Additionally, the user can choose a linker starting residue, ending residue, AA enrichment, AA depletion and/or protease sensitivity as a desired linker property in the recombinant fusion protein. Once a query is submitted, both the natural and artificial/empirical linkers in SynLinker are searched simultaneously, yielding a list of potential linker candidates satisfying the desired selection criteria together with information about the AA composition radar chart and the conformation of the selected linker, as well as the fusion protein structure and hydropathicity plot [350].

As for modeling-based approaches, the conformation and placement of functional units in fusion proteins, of which 3D structures are available from the PDB or homology modeling, can be predicted by computer-aided modeling. A modeling tool known as FPMOD was developed and can generate fusion protein models by connecting functional units with flexible linkers of proper lengths, defining regions of flexible linkers, treating the structures of all functional units as rigid bodies and rotating each of them around their flexible linker to produce random structures. This tool can extensively test the conformational space of fusion proteins and finally generate plausible models [351]. This tool has been applied to designing FRET-based protein biosensors for Ca^2+^ ion by qualitatively predicting their FRET efficiencies, and the predictions strongly agreed with the experimental results [352].

A similar modeling tool was developed for assembling structures of isolated functional units to constitute multidomain fusion proteins. However, this approach of assembling functional units is different from the method of testing conformational space. In this method, an ab initio protein-modeling method is utilized to predict the tertiary structure of fusion proteins, the conformation and placement of functional units and the linker structure. This method samples the degrees of freedom of the linker (in other words, domain assembly as a linker-folding problem) rather than those of the rigid bodies, as adopted in FPMOD. The method consists of an initial low-resolution search, in which the conformational space of the linker is explored using the Rosetta de novo structure prediction method. This is followed by a high-resolution search, in which all atoms are treated explicitly, and backbone and side chain degrees of freedom are simultaneously optimized. The obtained models with the lowest energy are often very close to the correct structures of existing multidomain proteins with very high accuracy [353].

A method called pyDockTET (tethered-docking) uses rigid-body docking to generate domain–domain complexes that are scored by the electrostatic and desolvation energy terms, as well as a pseudo-energy term reflecting restraints from linker end-to-end distances; in this manner, near-native pair-wise domain poses are selected. The optimal linker sequence length (in the number of residues) with the linker ends (defined as the distance between the Cα atoms of the two ends of a linker) is selected from a flexible linker database, which consists of 542 linkers with sequence lengths ranging from 2 to 29 AAs derived from the inter-domain linkers of multidomain structures in the PDB [354].

A fusion protein consisting of a protein called cell-traversal protein for ookinetes and sporozoites (CelTOS) antigen from *Plasmodium falciparum* (the deadliest of malaria species) and human IL-2 as an adjuvant was designed to develop a candidate vaccine against malaria. CelTOS and IL-2 were linked together directly or by using different flexible linkers, including (G)_8_, (G_4_S) and (G_4_S)_3_. Since the N-terminus of IL-2 and the C-terminus of CelTOS are critical to preserve their stability and bioactivity, the fusion protein was designed by linking the C-terminus of IL-2 with the N-terminus of CelTOS. The tertiary structures of the fusion proteins were predicted in silico by the I-TASSER online server (http://zhanglab.ccmb.med.umich.edu/I-TASSER/) [355]. The model with the highest confidence score (C-score: a scoring function based on the relative clustering structural density and the consensus significance score of multiple threading templates) was considered as the best model. The selected structures of the fusion proteins with different linkers were then validated and analyzed using a Ramachandran plot assessment [356]. All the results verified the (G_4_S)_3_ linker as the most suitable for separating these proteins [357].

The important issue to be addressed in structure prediction is the method of searching the large and complex conformational space to rapidly reach at the minimum energy structure, which is presumed to be the native fold. The genetic algorithm combined with an extremely fast technique to search the conformation space exhaustively and build a library of possible low-energy local structures for oligopeptides (i.e., the MOLS method), was applied to the protein structure prediction. At the first step, the protein sequence was divided into short overlapping fragments, and then their structural libraries were built using the MOLS method. At the second step, the genetic algorithm exploited the libraries of fragment structures and predicted the single best structure for the protein sequence. In the application of this combined method to peptides and small proteins, such as the avian pancreatic polypeptide (36 AAs), the villin headpiece (36 AAs), melittin (26 AAs), the transcriptional activator Myb (52 AAs) and the Trp zipper (16 AAs), it could predict their near-native structures [358].

The computer-aided rational design methods for fusion proteins are promising because these methods allow us to easily predict the desired conformation and placement of the functional units and linker structures of fusion proteins, and consequently select suitable candidate linker sequences. However, it is difficult to determine the unique conformation of flexible linkers due to many local minima in free energy. Furthermore, if changes in the conformation or arrangement of functional units are essential to display their activity, the linker conformation should also be changed to allow the movement of functional units, e.g., the N-terminal ATP-binding domain and unfolded substrate protein-binding domain connected with a hydrophobic peptide linker in heat shock protein 70 [359]. This complicated conformational transition issue makes it difficult to design optimum linkers for fusion proteins with multiple conformations. Therefore, the rational design of fusion proteins with desired properties and predictable behavior remains a daunting challenge.

## Conclusion

This review highlighted some of the recent developments in studies related to nanobio/bionanotechnology, including the applications of engineered biological molecules combined with functional nanomaterials in therapy, diagnosis, biosensing, bioanalysis and biocatalysis. Furthermore, this review focused on recent advances in biomolecular engineering for nanobio/bionanotechnology, such as nucleic acid engineering, gene engineering, protein engineering, chemical and enzymatic conjugation technologies, and linker engineering.

Based on creative chemical and biological technologies, manipulation protocols for biomolecules, especially nucleic acids, peptides, enzymes and proteins, were described. We also summarized the main strategies adopted in nucleic acid engineering, gene engineering, protein engineering, chemical and enzymatic conjugation technologies and linker engineering. Nucleic acid engineering based on the base-pairing and self-assembly characteristics of nucleic acids was highlighted as a key technology for DNA/RNA nanotechnologies, such as DNA/RNA origami, aptamers, ribozymes. Gene engineering includes direct manipulation technologies for genes, such as gene mutagenesis, DNA sequence amplification, DNA shuffling and gene fusion, which are powerful tools for generating enzymes, proteins, entire metabolic pathways, or even entire genomes with desired or improved properties. Two general strategies for protein engineering, i.e., rational protein design and directed evolution (i.e., high-throughput library screening- or selection-based approaches) were discussed. Conjugation technologies to site-specifically modify proteins with diverse natural and unnatural functionalities have been developed in the last two decades. These technologies range from classical chemical bioconjugation technologies, bioorthogonal chemical conjugations, protein chemical ligations and enzymatic conjugations, which were overviewed. Linker engineering for controlling the distance, orientation and interaction between functional components crosslinked in conjugates is also an important technology. The design and optimization strategies of chemical and biological linkers, such as oligonucleotides and polypeptides, were overviewed.

A variety of strategies are now available for designing and fabricating novel nanobiomaterials with highly ordered dimension and complexity based on biomolecular self-assembly characteristics governed by molecular interactions among nucleotides, peptides, proteins, lipids and small ligands, each of which focuses on design simplicity, high structural and functional control, or high fabrication accuracy [16–20, 106, 127, 132, 360–365]. Fundamentally, these properties are not mutually exclusive, and the relative weaknesses of each approach will be solved in the near future. Given the rapid recent progress in the biomolecular engineering and nanotechnology fields, the design of completely novel biomaterial-based molecular devices and systems with functions tailored for specific applications seems to be much easier and more feasible than before.
